# General Index K-Z

**Published:** 1834-06-01

**Authors:** 


| | Vol. | Page |
|---|---|---|
| Kidney, aqueous encysted tumor of.. | 20 | 23 |
| Kidney, encephaloid disease of the .. | 20 | 517 |
| Kidneys and bladder, extensive disease of the.................. | 11 | 267 |
| Kidneys, Dr. England on disorder of, | 12 | 481 |
| Kidneys, cases of disease in the .... | 18 | 190 |
| Kiernan, Mr. on anatomy of the liver | 20 | 305 |
| King and Queen's College, transactions of .................... | 1 | 355 |
| King George the Fourth .......... | 13 | 468 |
| King, Dr. on lithotrity and lithotomy | 17 | 24 |
| Kinglake, Dr. on colchicum........ | 1 | 224 |
| Kinglake, Dr. on hernia............ | 2 | 211 |
| Kings of France, post-mortem examinations of the............ | 12 | 259 |
| Kino effectual in a case of dropsy. .. | 7 | 134 |
| Kirby, Mr. his *extirpation* of the parotid. ................... | 4 | 546 |
| "Kirronosis," M. Lobstein on...... | 5 | 517 |
| Knee, neuralgia of the ............ | 10 | 57 |
| Knee, partial dislocation of the. .... | 10 | 582 |
| Knee-joint, wounded, treatment of.. | 4 | 399 |
| Knee-joint, case of excision of the .. | 7 | 303 |
| Knee-joint, extensive disease in .... | 9 | 574 |
| Knee-joint, fatal injury of the...... | 12 | 230 |
| Knee-joint, loose cartilages in the... | 12 | 562 |
| Knife-swallowing, a case of........ | 2 | 476 |
| Knife cut out from the stomach .... | 2 | 477 |
| Knight, Dr. on mental derangement, | 7 | 1 |
| Knives *not* blackened by arsenic.... | 3 | 182 |
| Knox, Dr. his translation of Cloquet's anatomy ............. | 11 | 117 |
| Knox, Dr. on amaurosis .......... | 13 | 206 |
| Koecker, Mr. on the teeth......... | 1 | 218 |
| Koecker, Mr. his dental surgery .... | 6 | 129 |
| Koecker, Mr. on diseases of the jaws, | 8 | 425 |
| Kolley, Dr. on iodine............. | 2 | 229 |
| Kuhn, M. on hydatids ............ | 19 | 201 |
| **L** | | |
| Labia pudendi, infiltration of ...... | 7 | 431 |
| Labia pudendi, elephantiasis of the.. | 12 | 508 |
| Labour, premature, cases of ...... | 1 | 307, 308 |
| Labour, case of cessation of........ | 3 | 414 |
| Labour, varieties of .............. | 10 | 105 |
| Labour, premature, induction of.... | 19 | 202, 420 |
| Labour impeded by malformations of the fœtus ................ | 20 | 489 |
| Labour, premature, induction of. .. | 20 | 503 |
| Labours, difficult, use of instruments in .................... | 15 | 91 |
| La Charité, report from .......... | 9 | 251 |
| Lacerated wound in the arm ...... | 12 | 174 |
| Lacerated arteries, cessation of hæmorrhage from .............. | 18 | 256 |
| Lacerated arteries, experiments on.. | 18 | 257 |
| Lacerated perinæum, cured by suture | 18 | 526 |
| Lacerated perinæum, operation for.. | 20 | 510 |
| Laceration of the womb, case of.... | 2 | 406 |
| Laceration of the uterus, Mr. Birch on | 8 | 335 |
| Laceration of the urethra—sloughing—death ................ | 8 | 453 |
| Laceration of uterus and vagina .... | 14 | 357 |
| Laceration of the urethra, cases of.. | 17 | 533 |
| Lacerations of the uterus, Dr. M'Keever on................ | 4 | 19 |
| Lacerations of the uterus, symptoms of ..................... | 4 | 21 |
| Lacing, tight, injurious effects of.... | 12 | 512 |
| Lactation, remarks on ............ | 16 | 154 |
| Lactation, preternatural, cases of .. | 17 | 201 |
| Lactic acid (free) in the stomach .. | 2 | 145 |
| Lactuca virosa, its effect on the pulse, | 7 | 102 |
| Laennec on auscultation .......... | 2 | 61 |
| Laennec's report on fever ......... | 2 | 240 |
| Laennec, M. his hospital reports.... | 4 | 97, 569 |
| Laennec, M. on mediate auscultation, | 8 | 103 |
| Laennec, review of his chapter on peripneumony ............... | 8 | 289 |
| Laennec, M. on diseases of the chest, | 10 | 420 |
| Laennec, Dr. on hypertrophy of the brain .. ......... ...... | 10 | 565 |
| Laennec on the sounds of the heart . | 20 | 133 |
| Laidlaw, Mr. his surgical report.... | 16 | 594 |
| Lallemand's amputation of the lower jaw .................. | 1 | 213 |
| Lallemand, Dr. on the brain ...... | 1 | 265 |
| Lalor, Miss, her miraculous cure.... | 1 | 176 |
| Lambe, Dr. on Thames water ...... | 9 | 268 |
| Lambert and the medical societies of London ................... | 10 | 493 |
| Lambert, Mr. his accuracy ........ | 10 | 563 |
| Lambert, the late Mr............. | 14 | 478 |
| Lancaster Lunatic Asylum......... | 20 | 546 |
| *Lancet* report; a good specimen.... | 6 | 195 |
| Lancet morality, or, "The Youths of Fifteen," a *farce* ........ . | 6 | 268 |
| Lancet, review of hospital reports in, | 7 | 241 |
| Lancet, falsehoods of the.......... | 8 | 541 |
| Lancet, ratiocination of the........ | 8 | 550 |
| Lancet, character of the........... | 11 | 153 |
| Lancet, candour of the............ | 11 | 181 |
| Lancetted stilettes in stricture...... | 14 | 195 |
| Langford on the Asiatic cholera .... | 20 | 145 |
| Language, loss of; Dr. Jackson's case | 11 | 259 |
| Language, singular affection of organ of ...................... | 18 | 539 |
| Lanza, Professor, his new medical logic ......................... | 5 | 128 |
| Larrey, Baron, his treatment of traumatic erysipelas .......... | 5 | 205 |
| Larrey, B. his treatment of hydrocele | 8 | 501 |
| Larrey, M. on the heart .......... | 13 | 535 |
| Larrey, M. on the testis .......... | 13 | 540 |
| Larrey, M. his clinique chirurgicale, | 15 | 1 |
| Larrey's mode of tapping the pericardium..................... | 15 | 514 |
| Larrey, M. Hippolite, on the wounded of July ................ | 16 | 49 |
| Laryngeal phthisis, herring-milt not *always* a cure for ........... | 4 | 566 |

| | Vol. | Page |
|---|---|---|
| Laryngeal phthisis, case of | 13 | 543 |
| Laryngeal fistula, cured | 18 | 194 |
| Laryngeal spasm, Dr. Walker on | 19 | 363 |
| Laryngitis, Mr. Carmichael on | 2 | 223 |
| Laryngitis, extensive bleedings in | 3 | 207 |
| Laryngitis, Dr. Francis's case of | 3 | 209 |
| Laryngitis, Dr. Johnson's case of | 3 | 210 |
| Laryngitis œdematosa, its seat and symptoms | 6 | 390 |
| Laryngitis œdematosa, treatment of, | 6 | 393 |
| Laryngitis, M. Martinet on | 8 | 168 |
| Laryngitis—bronchotomy successful, | 10 | 263 |
| Laryngitis—tracheotomy successful, | 11 | 467 |
| Laryngitis, cases of | 13 | 173 |
| Laryngitis, chronic; tracheotomy successful | 14 | 176 |
| Laryngitis—tracheotomy | 15 | 464 |
| Laryngitis, M. Cruveilhier on | 16 | 591 |
| Laryngotomy, case of | 19 | 250 |
| Laryngotomy, two cases of | 19 | 540 |
| Larynx, extraordinary affection of its muscles | 3 | 385 |
| Larynx, ulceration of it | 3 | 468 |
| Larynx, phagedenic ulceration of | 3 | 472 |
| Larynx, diseased, tracheotomy for | 3 | 550 |
| Larynx, ulceration of, Mr. Fereday | 7 | 554 |
| Larynx, foreign bodies in the; bronchotomy | 10 | 517 |
| Larynx, foreign body in, extracted | 13 | 465 |
| Larynx, œdema of, tracheotomy | 14 | 500 |
| Larynx, mercury in chronic affections of | 18 | 231 |
| Larynx, Mr. Wood on inflammation of | 18 | 343 |
| Larynx, encysted tumor on the | 20 | 519 |
| Late hours, ill effects of | 3 | 195 |
| Lateral curvature of the spine | 1 | 303 |
| Lateral curvature, Mr. Bampfield on, | 2 | 133 |
| Lateral curvature, Mr. Shaw on | 3 | 366 |
| Lateral depression of the thorax, M. Dupuytren on | 9 | 311 |
| Lateral curvature, instruments in | 15 | 419 |
| Latham, Dr. his work on the Penitentiary endemic | 3 | 1 |
| Latham, Dr. on the duration of fever | 19 | 544 |
| Laudanum, as an application in dissection-wounds | 3 | 250 |
| Laudanum extracted from the stomach | 3 | 276 |
| Laudanum, a case of poisoning by | 4 | 270 |
| Laudanum, mysterious case of poisoning by | 11 | 551 |
| Laurel water, its effects upon dogs | 3 | 186 |
| Lavements, Mr. Scott on | 12 | 499 |
| Law, interpretation of, in cases of pregnancy | 20 | 121 |
| Lawrence and radical reform; universal equality | 6 | 267 |
| Lawrence's lecture on the division of labour | 6 | 569 |
| Lawrence, Mr. on dislocations of vertebræ | 8 | 320 |
| Lawrence, Mr. on erysipelas | 9 | 361 |

| | Vol. | Page |
|---|---|---|
| Lawrence, Mr. his letter to Dr. Johnson | 8 | 525 |
| Lawrence, Mr. his election | 9 | 550 |
| Lawrence, Mr. on dissection-wounds, | 12 | 529 |
| Lawrence, Mr. on corns | 13 | 438 |
| Lawrence, Mr. on the venereal diseases of the eye | 14 | 80 |
| Lawrence, Mr. on the same | 14 | 116 |
| Lawrence, Mr. on inflammation of veins | 14 | 212 |
| Lawrence, Mr. on tumors | 18 | 333 |
| Lawrence, Mr. on diseases of the eye | 20 | 40 |
| Lawrence, Mr. on purulent ophthalmia | 20 | 43 |
| Lawrence, Mr. on strumous ophthalmia | 20 | 44 |
| Lawrie, Dr. on cholera at Sunderland | 16 | 541 |
| Lazaretto in Harley Street | 10 | 246 |
| Leach, Dr. versus Dr. Veitch | 1 | 237 |
| Lead suggested in aneurismal diseases | 4 | 200 |
| Lead, action of air and water on | 12 | 490 |
| Lead, palsy from | 16 | 29 |
| Lead, white, workers in; diseases of, | 18 | 109 |
| Leadenhall-street, medical tyranny | 13 | 121 |
| Leamington waters, analysis of | 10 | 27 |
| Leander, remarkable cases of scurvy in the | 1 | 233 |
| Leaping ague of Angus-shire | 11 | 151 |
| Learned men bad practitioners | 3 | 166 |
| Lectures of Sir A. Cooper on surgery, | 4 | 98 |
| Lectures on anatomy | 16 | 239 |
| Lectures, clinical, M. Dupuytren's | 19 | 68 |
| Lectures, introductory, by Drs. Grant and Watson, and Messrs. Wardrop and Guthrie | 20 | 152 |
| Lecturers, private, bye-law affecting them | 1 | 241 |
| Lecturers, Apothecaries' Company's rules for | 15 | 145 |
| Lee, Dr. on the nutrition of the fœtus | 7 | 504 |
| Lee, Dr. on plegmatia dolens | 11 | 380 |
| Lee, Dr. on uterine phlebitis | 12 | 376 |
| Lee, Dr. on uterine inflammation | 15 | 425 |
| Lee, Dr. on abortion | 16 | 446 |
| Lee, Dr. on the placenta | 17 | 454 |
| Lee, Dr. R. on diseases of women | 19 | 58 |
| Lee, Dr. R. on uterine hæmorrhage | 19 | 67 |
| Lee, Mr. E. on nervous affections | 20 | 148 |
| Leech, Drs. Johnson & Price on the, | 5 | 52 |
| Leech, diseases of the, and their prevention | 5 | 66 |
| Leech-importers, their enormous receipts | 5 | 72 |
| Leech-bites, fatal hæmorrhage from, | 8 | 552 |
| Leech-bites, death from | 19 | 216 |
| Leeches, nostrum for making them *take* | 3 | 587 |
| Leeches, porter a good *whet* for | 4 | 285 |
| Leeches, Dr. Pallas on the management of | 5 | 289 |
| Leeches, fatal application of | 12 | 76 |
| Leeches, preservation of | 19 | 209 |
| Leeds, epidemic cholera at, in 1825 | 16 | 636 |

| | Vol. | Page |
|---|---|---|
| Lefevre, Dr. on cholera | 16 | 213 |
| Leg, report on sloughy abscess of the | 13 | 281 |
| Legacy left by M. Portal | 19 | 506 |
| Legallois, M. on gastro-enteralgia | 9 | 271 |
| Lemon-juice, not a specific in sea-scurvy | 1 | 232 |
| Lens, regeneration of the | 19 | 197 |
| Lentigo, or freckles, M. Biett on | 17 | 361 |
| Lepra Arabum, its symptoms and progress | 4 | 444 |
| Lepra, venereal | 6 | 606 |
| Lepra, M. Biett on | 16 | 402 |
| Lerminier, Dr. his doctrines | 3 | 101 |
| Leslie, Mr. on gangrenous ulcer | 11 | 374 |
| Leuca, Baron Alibert on | 12 | 523 |
| Leucorrhœa | 12 | 196 |
| Leucorrhœa, nitrate of silver in | 12 | 517 |
| Leucorrhœa, &c. Mr. Jewel on | 13 | 416 |
| Leucorrhœa, actual seat of | 13 | 417 |
| Leucorrhœa, ergot of rye in | 15 | 520 |
| Leucorrhœa, Boivin and Duges on | 20 | 222 |
| Leucorrhœa, secale cornutum in | 20 | 451 |
| Leveillé, Dr. on delirium tremens | 9 | 509 |
| Levratt's operation for tympanites | 1 | 220 |
| Lewis Cornaro and Mr. Abernethy's golden rules | 6 | 22 |
| Libel, age of | 10 | 266 |
| Liberality *v.* illiberality | 8 | 480 |
| Liberty of the press—more lawsuits! | 8 | 456 |
| Liberty of the press | 12 | 438 |
| Licentiates of the College of Physicians, their degradation | 5 | 636 |
| Licentiates' petition to Parliament | 19 | 566 |
| Lichen, M. Biett on | 11 | 567 |
| Lichen, M. Biett on | 16 | 402 |
| Lichen vulgaris, a diuretic | 17 | 523 |
| Lichtenstadt, Dr. on cholera | 16 | 164 |
| Liddell's translation of Esquirol's treatise | 19 | 352 |
| Life and mind, Dr. Kennedy on | 13 | 145 |
| Life of Sir Humphry Davy | 14 | 423 |
| Life and organization | 15 | 77 |
| Life, value of, in males and females | 18 | 493 |
| Life continuing after craniotomy | 20 | 204 |
| Ligature and knife, comparison of, for piles | 2 | 292 |
| Ligature on varicose veins, its success in France | 4 | 247 |
| Ligature beyond the aneurismal tumor | 7 | 209 |
| Ligature of the pneumo-gastric nerves | 7 | 410 |
| Ligature of the common iliac artery, | 7 | 416 |
| Ligature of arteries, ultra tumorem | 8 | 552 |
| Ligature *ultra* aneurisma, Mr. Lawrence's opinion | 8 | 557 |
| Ligature of the internal iliac artery | 9 | 232 |
| Ligature of the femoral artery & vein | 10 | 591 |
| Ligature ultrà tumorem, for supposed carotid aneurism | 12 | 165 |
| Ligature for polypus uteri | 12 | 326 |
| Ligature ultrà tumorem | 12 | 501 |
| Ligature for popliteal aneurism | 13 | 286 |
| Ligature instrument, Baron Graefe's, | 20 | 218 |

| | Vol. | Page |
|---|---|---|
| Ligature, amputation of the leg by a, | 20 | 235 |
| Ligatures in abdominal wounds | 3 | 338 |
| Ligatures *beyond* the aneurism; Deschamps and Sir A. Cooper's cases | 4 | 529 |
| Ligatures, flat, in aneurism | 17 | 444 |
| Light, influence of, on animal bodies, | 18 | 26 |
| Lightning, effects of | 2 | 149, 199 |
| Lime, chloruret of, mode of using it as an anti-putrescent | 6 | 352 |
| Limosis dyspepsia, Dr. Good on | 11 | 93 |
| Linea alba, rupture of; a case | 5 | 242 |
| Liniment, iodine, formula for it | 4 | 5 |
| Liniment of St. John Long | 14 | 257 |
| Liniments and lavements | 3 | 353 |
| Lip, ulcers of, cured | 10 | 535 |
| Lip, extensive prolongation of its mucous membrane | 11 | 566 |
| Liquor opii acetatis, formula for | 9 | 208 |
| Liquor cinchonæ cordifoliæ, Mr. Battley's | 11 | 484 |
| Liquor potassæ in ovarian disease | 12 | 431 |
| Lisfranc M. on white swelling | 5 | 546 |
| Lisfranc on fracture of the spine | 5 | 560 |
| Lisfranc, his hospital reports | 5 | 570 |
| Lisfranc, M. his extirpation of the parotid | 6 | 564 |
| Lisfranc, M. on cancer of the rectum | 13 | 148 |
| Lisfranc on amaurosis | 19 | 199 |
| Liston, Mr. operation for tetanus | 1 | 217 |
| Liston, Mr. on surgery | 16 | 242 |
| Liston, Mr. his elements of surgery, | 18 | 328 |
| Liston, Mr. on lithotomy & lithotrity, | 18 | 331 |
| Literary justice, specimen of | 8 | 569 |
| Literary property and reclamations | 12 | 278 |
| Litharge, in wines, test of | 4 | 313 |
| Lithontripteur, or stone-borer | 1 | 472 |
| Lithontripteur, success of, in France, | 5 | 536 |
| Lithontripteur | 10 | 191 |
| Lithontripteur used without success, | 10 | 454 |
| Lithoplatomy, Dr. Buchanan on | 14 | 265 |
| Lithotome, new, by Gattei | 20 | 203 |
| Lithotomists, native, in India | 17 | 526 |
| Lithotomy, Mr. Key on | 1 | 206 |
| Lithotomy, recto-vesical | 2 | 474 |
| Lithotomy, Mr. Liston's remarks on, | 3 | 247 |
| Lithotomy, Mr. Averill's operation | 3 | 539 |
| Lithotomy, hæmorrhage in | 4 | 538 |
| Lithotomy inducing mania, a curious case | 5 | 114 |
| Lithotomy, some hints on | 5 | 118 |
| Lithotomy, high operation in; Dr. Ballingall's case | 6 | 83 |
| Lithotomy, Mr. Lizars' observations on | 6 | 273 |
| Lithotomy, some cases of | 6 | 549 |
| Lithotomy, successful | 7 | 267 |
| Lithotomy in an old person, fatal case | 7 | 526 |
| Lithotomy, fatal, some cases of, with remarks | 7 | 532 |
| Lithotomy, fatal, case of | 7 | 555 |

| | Vol. | Page |
|---|---|---|
| Lithotomy, Panton Square, cases of, | 8 | 194 |
| Lithotomy; vile conduct of a reporter | 9 | 167 |
| Lithotomy and lithotrity | 10 | 69 |
| Lithotomy, mode of operating | 10 | 231 |
| Lithotomy—M. Dupuytren foiled | 10 | 453 |
| Lithotomy, cases of | 10 | 465 |
| Lithotomy, difficult case of | 10 | 538 |
| Lithotomy after fistula ani | 11 | 508 |
| Lithotomy, Mr. Stanley on | 12 | 69 |
| Lithotomy, mode of performing | 12 | 70 |
| Lithotomy, recto-vesical, case of | 14 | 187 |
| Lithotomy, Mr. Brodie on the operation of | 15 | 259 |
| Lithotomy, Mr. Brodie's method | 15 | 261 |
| Lithotomy, after treatment | 15 | 262 |
| Lithotomy, on death from | 15 | 262 |
| Lithotomy, high operation | 15 | 264 |
| Lithotomy in the female | 15 | 265 |
| Lithotomy, cases of failure in | 15 | 365 |
| Lithotomy, immediate death from | 15 | 368 |
| Lithotomy in the 15th century | 15 | 405 |
| Lithotomy in the female, various modes | 15 | 515 |
| Lithotomy, for the extraction of a catheter | 15 | 522 |
| Lithotomy in the perineum | 17 | 29 |
| Lithotomy, cases of | 17 | 31 |
| Lithotomy, hæmorrhage after | 17 | 34 |
| Lithotomy by the rectum | 17 | 35 |
| Lithotomy above the pubes | 17 | 36 |
| Lithotomy, case of, in a female child, | 17 | 233 |
| Lithotomy for removal of piece of catheter | 17 | 528 |
| Lithotomy, Dr. Macfarlane on | 18 | 127 |
| Lithotomy, death from | 18 | 564 |
| Lithotomy, M. Dupuytren on | 19 | 87 |
| Lithotomy, M. Clot Bey on | 19 | 489 |
| Lithotritic instruments, considerations on | 16 | 148 |
| Lithotritist, picture of a | 16 | 153 |
| Lithotrity; Baron Heurteloup's instruments | 11 | 573 |
| Lithotrity in the female | 12 | 209 |
| Lithotrity and lithotomy | 12 | 227 |
| Lithotrity, Mr. Costello on | 13 | 257 |
| Lithotrity—Mr. Costello | 14 | 569 |
| Lithotrity, remarks on | 15 | 266 |
| Lithotrity, Heurteloup on | 16 | 134 |
| Lithotrity and lithotomy, parallel between | 16 | 138 |
| Lithotrity, history of | 16 | 139 |
| Lithotrity, circumstances favourable to, &c. | 16 | 150 |
| Lithotrity & lithotomy, Dr. King on, | 17 | 24 |
| Lithotrity | 17 | 36 |
| Lithotrity, instruments of | 17 | 37 |
| Lithotrity objections against, considered | 17 | 38 |
| Lithotrity, case of | 17 | 186 |
| Lithotrity, report upon | 19 | 503 |
| Lithotrity, M. d'Etioles on | 20 | 232 |
| Liver, state of, in gout | 4 | 45 |
| Liver, functions of the | 9 | 108 |
| Liver, Mr. Abernethy on the functions of the | 9 | 154 |
| Liver, cases of hydatid cyst in | 9 | 499 |
| Liver and lungs, purulent depôts in, | 10 | 169 |
| Liver, acute diseases of the | 10 | 344 |
| Liver, chronic diseases of the | 10 | 347 |
| Liver, Mr. Annesley on abscess in the | 10 | 435 |
| Liver, cysts on its convex surface | 11 | 535 |
| Liver, on morbid affections of the | 13 | 320 |
| Liver, state of, in icterus | 14 | 494 |
| Liver, Andral on diseases of the | 15 | 85 |
| Liver, cancer of the | 15 | 89 |
| Liver, case of rupture of the | 15 | 269 |
| Liver, abscess over the | 15 | 274 |
| Liver, abscess of the | 17 | 206 |
| Liver, cases of abscess of | 17 | 523 |
| Liver, diseases of, by Mr. G. H. Bell, | 19 | 415 |
| Liver, mortification of the | 19 | 443 |
| Liver, aqueous encysted tumors of the | 20 | 11 |
| Liver, Mr. Kiernan's anatomy of the, | 20 | 305 |
| Liver, lobules of the | 20 | 307 |
| Liver, portal canals of the | 20 | 309 |
| Liver—hepatic veins, &c. | 20 | 311 |
| Liver, structure of its lobules | 20 | 312 |
| Liver, red and yellow substances of the | 20 | 313 |
| Liver, Mr. Geddes on abscess of the | 20 | 365 |
| Liver, inflammation of the | 20 | 369 |
| Liver-cough, Dr. Brooke on | 2 | 208 |
| Liverpool Medical Society | 20 | 188 |
| Lives of British physicians | 13 | 478, 488 |
| Lizars, Mr. on extirpation of ovaria, | 2 | 215 |
| Lizars, Mr. on diseased ovaria | 3 | 335 |
| Lizars, Mr. on the division of nerves, | 13 | 271 |
| Lobelia inflata in asthma | 9 | 208 |
| Local injury, its effects on the constitution | 5 | 104 |
| Local affections of the nerves | 6 | 424 |
| Local malignant disease, Mr. Travers on | 11 | 385 |
| Locality, influence of, on cholera | 16 | 205 |
| Lock Hospital, report from | 20 | 269 |
| London University, remarks on the, | 9 | 433 |
| London University, opening of the | 10 | 184 |
| London University, diploma of | 13 | 204 |
| London College of Medicine | 14 | 572 |
| London University, vile proceedings at the | 15 | 330 |
| London physicians, memorial of the, | 19 | 192 |
| Long, Dr. his cures of consumption, | 9 | 533 |
| Longevity of authors, on the | 19 | 318 |
| Longitudinal sinus, varicose condition of | 4 | 514 |
| Loose substances removed from the knee | 10 | 181 |
| Loose cartilage in the knee-joint | 10 | 524 |
| Loose cartilages in the knee-joint | 12 | 562 |
| Loss of blood, effects of | 4 | 352 |
| Loss of blood, Dr. Hall on | 13 | 323 |
| Loss of blood, death from | 14 | 491 |
| Loss of blood, Dr. M. Hall on | 18 | 354 |

| | Vol. | Page |
|---|---|---|
| Loudon, Dr. on the Leamington Spa, | 10 | 24 |
| Louis, M. on a peculiar gastritis.... | 2 | 173 |
| Louis, M. his interesting paper on phthisis | 4 | 492 |
| Louis, M. his case of sub-acute metritis, &c. | 5 | 244 |
| Louis, M. on abscess of the liver.... | 6 | 201 |
| Louis, M. on pericarditis | 13 | 429 |
| Louis, M. on pericarditis | 19 | 49 |
| Louis XIV., birth of | 20 | 159 |
| Lower jaw, amputation of | 1 | 213 |
| Lower jaw, amputation | 7 | 289 |
| Lower jaw, compound fracture of .. | 7 | 529 |
| Lower jaw, amputation of half the.. | 9 | 258 |
| Lower jaw, amputation of the | 10 | 557 |
| Lower lip, cancer of the | 11 | 398 |
| Lower jaw, cancer of the | 11 | 399 |
| Lower lip destroyed—rhino-plastic operation | 12 | 268 |
| Lower jaw, half removed | 12 | 469 |
| Lower jaw, case of cancer of | 13 | 550 |
| Lower jaw, removal of great part of, | 13 | 555 |
| Lower jaw, removal of part of | 15 | 540 |
| Lower extremity, swelling of, post febrem | 10 | 180 |
| Lucid intervals in insanity | 13 | 91 |
| Lues; Mr. Hunter's dicta erroneous, | 4 | 392 |
| Lugol, M. on scrofula | 16 | 109 |
| Luke, Mr. his case of hæmorrhage .. | 13 | 263 |
| Lumbago, with metastasis to testicle, | 20 | 544 |
| Lumbar abscess in a horse | 10 | 243 |
| Lumbar abscess pointing behind the trochanter | 11 | 270 |
| Lumbar abscess, remarks | 17 | 519 |
| Lumbar abscess, cases of | 17 | 520 |
| Lumbricus, escape of, through perforated stomach | 14 | 364 |
| Lunacy—insanity—the speculations of Dr. Haslam | 8 | 243 |
| Lunacy, shameful laxity in granting certificates of | 9 | 30 |
| Lunacy *versus* sanity | 12 | 433 |
| Lunar caustic, Mr. Higginbottom's essay on | 4 | 379 |
| Lunar caustic, Mr. Higginbottom's paper on | 5 | 260 |
| Lunar caustic in urethral stricture.. | 7 | 69 |
| Lunar caustic, in wounds, bruises, &c. | 7 | 153 |
| Lunar caustic, employment of, in plastic inflammation | 9 | 459 |
| Lunar caustic, Dr. Hood on | 11 | 211 |
| Lunar influence | 13 | 485 |
| Lunatic asyla, disgraceful scenes in, | 8 | 246 |
| Lunatic asylums, Sir Andrew Halliday on | 9 | 25 |
| Lunatic asyla, abuses in and of | 9 | 33 |
| Lunatic asylums, regulation of | 9 | 228 |
| Lunatics, number of, in England and Wales | 9 | 27 |
| Lunatics, Dr. Conolly on | 13 | 306 |
| Lung, scirrhus of the | 13 | 520 |
| Lung, Dr. Tweedie on abscess of.... | 16 | 449 |

| | Vol. | Page |
|---|---|---|
| Lung, case of abscess in the | 18 | 182 |
| Lung, encephaloid disease of the.... | 20 | 439 |
| Lung, calcareous incrustation on the, | 20 | 501 |
| Lungs, fœtal | 1 | 314 |
| Lungs, consequences of putrefaction in them | 1 | 345 |
| Lungs, causes of their floating in water, distinguished | 1 | 346, 347 |
| Lungs, weight of, a test | 1 | 348, 349 |
| Lungs, case of inflammation of the.. | 3 | 517 |
| Lungs, deplorable tendency of to disease | 4 | 494 |
| Lungs, wound of the, a severe case.. | 5 | 204 |
| Lungs, a case of sudden congestion of the | 5 | 292 |
| Lungs, morbid anatomy of the | 9 | 272 |
| Lungs and liver, abscess in, after operations | 9 | 490 |
| Lungs, Dr. Mills on diseases of the.. | 11 | 79 |
| Lungs, state of, in phthisis | 12 | 98 |
| Lungs, emphysema of the | 12 | 200 |
| Lungs and liver, medullary sarcoma, | 12 | 274 |
| Lungs, Dr. Philip on the diseases of, | 13 | 318 |
| Lungs, on malignant diseases of the, | 13 | 513 |
| Lungs, action of cold on the | 14 | 147 |
| Lungs, putrefactive disorganization, | 14 | 549 |
| Lungs, on the circulation in the.... | 15 | 252 |
| Lungs, injury and inflammation of.. | 15 | 270 |
| Lungs, case of emphysema of the .. | 17 | 453 |
| Lungs, gangrene of the | 19 | 209 |
| Lungs, paralysis of the | 19 | 210 |
| Lungs, diseases of, Dr. Williams on.. | 20 | 132 |
| Lupus, cases of | 11 | 454 |
| Lupus, M. Biett on | 11 | 504 |
| Lupus treated by the iodine | 12 | 570 |
| Lupus, M. Biett on | 17 | 363 |
| Lupus, varieties of | 17 | 364 |
| Lupus, diagnosis and treatment of .. | 17 | 366 |
| Lupus, Mr. Macfarlane on | 17 | 516 |
| Lupus, cases of | 17 | 517 |
| Lupus, causes of | 17 | 518 |
| Lusus naturæ, two instances of | 5 | 399 |
| Luxation of vertebræ, M. Dupuytren on | 19 | 68 |
| Lying-in women, disorders of the mind in | 11 | 359 |
| Lymphatics, anatomy and use of.... | 18 | 214 |

### M

| | Vol. | Page |
|---|---|---|
| Macauley, Dr. on the effects of lightning | 2 | 199 |
| M'Cleod, Dr. his circular | 6 | 285 |
| M'Cormac, Mr. on stammering | 9 | 193 |
| M'Cormac, Dr. on palm oil | 17 | 207 |
| M'Cormac, Dr. on cholera | 18 | 152 |
| Macculloch, Dr. on remittents and intermittents | 9 | 49, 289 |
| Macculloch, Dr. on neuralgia | 10 | 49, 357 |
| M'Fadzen, Mr. on water-dressing .. | 12 | 551 |
| Macfarlane, Dr. on polypus uteri.... | 10 | 373 |

| | Vol. | Page |
|---|---|---|
| Macfarlane, Dr. on cramp of the stomach | 11 | 489 |
| Macfarlane, Mr. his clinical reports, | 17 | 515 |
| Macfarlane, Dr. his clinical reports.. | 18 | 126 |
| Macfarlane, Mr. on aneurism, &c. .. | 18 | 505 |
| Macfarlane, Dr. clinical reports of .. | 19 | 529 |
| Machinery, spinal, varieties of ..... | 13 | 57 |
| Machines for pressing supposed dislocated vertebræ | 3 | 376 |
| Macilwain, Mr. on strictures | 7 | 69 |
| Macilwain, Mr. on stricture | 13 | 193 |
| Macilwain, Mr. on cure of nævi.... | 20 | 174 |
| Macilwain, Mr. on porrigo | 20 | 348 |
| M'Keever, Dr. on laceration of the uterus | 4 | 19 |
| Mackenzie, Mr. on closure of the pupil in iritis | 4 | 239 |
| Mackenzie, Mr. on croup | 4 | 261 |
| Mackenzie, Mr. & Dr. Bretonneau, their claims | 5 | 436 |
| Mackenzie, Mr. on ophthalmia | 6 | 173 |
| Mackenzie, Mr. on the pathology of croup | 6 | 289 |
| Mackenzie, Mr. on catarrho-rheumatic ophthalmia | 7 | 136 |
| Mackenzie, Mr. on choroiditis | 13 | 253 |
| Mackenzie, Mr. on glaucoma | 13 | 491 |
| Mackenzie, Mr. on diseases of the eye | 14 | 397 |
| Mackenzie, Mr. on iritis | 15 | 53 |
| Mackenzie, Mr. on strabismus | 15 | 523 |
| M'Kinnal, Dr. on a malignant yellow fever | 14 | 38 |
| Mackintosh, Dr. on Cullen's system, | 8 | 273 |
| Mackintosh, Dr. on amenorrhœa.... | 20 | 161 |
| Maclachlan, Dr. his case of aneurism of the temporal artery | 8 | 497 |
| Maclachlan, Dr. his hospital report.. | 10 | 586 |
| Maclean, Dr. on quarantine | 2 | 1 |
| Maclean, Mr. on abscess of kidney.. | 19 | 159 |
| Maclean, Dr. on umbilical hernia. .. | 19 | 453 |
| M'Lellan, Dr. his Parisian formulary, | 12 | 540 |
| Maclelland, Dr. amputation of the lower jaw | 2 | 210 |
| Macleod *v.* Wakley | 8 | 528 |
| M'Lure's case of obliterated urethra.. | 1 | 215 |
| Macrobin, Dr. on insanity | 16 | 538 |
| Maculæ in children, best treatment of | 11 | 173 |
| Maculæ, M. Biett on | 17 | 360 |
| Madame Montmorenci | 13 | 57 |
| Madden, Mr. his travels in Turkey, &c. | 11 | 141 |
| Madden, Mr. on the plague | 11 | 323 |
| Madden, Mr. on the infirmities of genius | 19 | 316 |
| Madeira, its climate, &c | 1 | 486 |
| Madeira, its climate for phthisis .... | 7 | 173 |
| Madeira best adapted for phthisis ... | 12 | 92 |
| Mad-house treatment of madness ... | 13 | 302 |
| Madness, detection and treatment of, | 13 | 301 |
| Madness, the dungeon and jacket for, | 13 | 304 |
| Madras Board, report of, on cholera, | 16 | 497 |
| Madras medical reports | 17 | 408 |
| Mænorrhagia, ipecacuan emetics in, | 9 | 178 |
| Mænorrhagia produced by mercury.. | 10 | 471 |
| Magnetism and electricity, identity of | 18 | 458 |
| Magnetism, animal, effects of | 19 | 184 |
| Majendie, M. strictures on his experiments | 1 | 189 |
| Majendie's, late experiments | 1 | 440 |
| Majendie, M. on gravel | 18 | 49 |
| Majendie, lectures of, on the cholera, | 19 | 245 |
| Majendie on the sounds of the heart, | 20 | 133 |
| Malaria, Dr. Macculloch on | 8 | 1, 370 |
| Malaria, the cause of various affections | 8 | 2 |
| Malaria, its influence on the digestive organs | 9 | 57 |
| Malaria, Dr. Brown on | 10 | 87 |
| Malaria of Italy | 14 | 457 |
| Malaria, Volta's experiments on.... | 16 | 490 |
| Malden, Dr. on inflammation | 10 | 198 |
| Malden, Mr. on the pulmonary circulation | 15 | 252 |
| Male, Dr. on medical jurisprudence.. | 1 | 337 |
| Males and females, value of life in.. | 18 | 493 |
| Males and females, numbers of | 20 | 508 |
| Malformation of the heart, case of .. | 1 | 301 |
| Malformation of the eyes, Dr. Seiler on | 20 | 370 |
| Malformations of the œsophagus.... | 20 | 230 |
| Malformations of fœtus impeding labour | 20 | 489 |
| Malignancy, signification of | 11 | 387 |
| Malignancy of tumors at first innocent | 14 | 521 |
| Malignant tumor growing from the vagina | 9 | 158 |
| Malignant intermittent, case of .... | 11 | 278 |
| Malignant diseases, Mr. Travers on.. | 11 | 385 |
| Malignant tumors | 13 | 47 |
| Malignant diseases of the lungs .... | 13 | 513 |
| Malignant diseases all curable | 17 | 328 |
| Malignant tumor in calf of leg, cases, | 18 | 279 |
| Malingering, Dr. Cheyne on | 8 | 309 |
| Malingering, Mr. Marshall's hints on, | 9 | 90 |
| Malingering, audacious instance of.. | 9 | 95 |
| Malingering | 17 | 262 |
| Malta, Dr. Hennen on the climate of, | 14 | 336 |
| Malta, endemic diseases of | 14 | 338 |
| Malta, plague at | 14 | 339 |
| Malta, pulmonary affections at | 14 | 342 |
| Malvern waters, Mr. Addison on the, | 10 | 24 |
| Mamma, excision of the | 12 | 191 |
| Mamma, cases of hypertrophy of.... | 20 | 464 |
| Mammæ, neuralgia of the | 17 | 461 |
| Mammæ, hypertrophy of | 19 | 493 |
| Mammæ, sympathy between them and the uterus | 20 | 171 |
| Mammary diseases, classification of.. | 7 | 162 |
| Mammary abscess, Dr. Beatty on. .. | 20 | 448 |
| Man's appearance in marshy countries | 3 | 321 |
| Man, natural history of, | 17 | 170 |
| Manchester, numbers of the sick of.. | 16 | 78 |
| Mandate, infamous, of Louis Philippe | 17 | 445 |

| | Vol. | Page |
|---|---|---|
| Manec, M. on ligature of arteries.... | 18 | 67 |
| Mania, curious and fatal case of .... | 5 | 203 |
| Mania from sobriety.............. | 9 | 181 |
| Man-midwife, Sir Anthony's horror of | 13 | 482 |
| Mansfield, Dr. on the antiquity of the Cæsarian section.......... | 4 | 473 |
| Manson, Dr. on the effects of iodine, | 4 | 1 |
| Manual of pharmacy, Mr. Brande's.. | 3 | 141 |
| Manual of pathology, M. Martinet's, | 6 | 407 |
| Manufacturing poor, Mr. Roberton on the .... .............. | 16 | 77 |
| Manwell, Dr. on hydrocephalus .... | 1 | 479 |
| March of intellect................ | 9 | 262 |
| Marley, Mr. on diseases of children.. | 12 | 553 |
| Marrow, spinal, cases of destruction of it ...................... | 4 | 181 |
| Marsh, Dr. on fever.............. | 8 | 55 |
| Marsh-fever, Dr. Macculloch's views of | 9 | 50 |
| Marsh-fever, proximate cause of.... | 9 | 61 |
| Marsh-fever, treatment of ......... | 9 | 62 |
| Marshall, Mr. his evidence on Fenning's trial.. ............... | 3 | 180 |
| Marshall Mr. on vaccination ....... | 12 | 499 |
| Marshall, Dr. his cases of cholera... | 16 | 130 |
| Marshall Hall, Dr. on abstinence ... | 16 | 450 |
| Marshall, Mr. on the mortality of the metropolis .............. | 17 | 243 |
| Marshall, Mr. case of ovarian tumor, | 20 | 177 |
| Marshes communicating with the sea, M. Georgini on .......... | 5 | 262 |
| Marshes and swamps, remarks on .. | 8 | 11 |
| Martin, Jonathan, his trial ........ | 11 | 221 |
| Martinet, Dr. on neuritis.......... | 2 | 165 |
| Martinet, M. on individual idiosyncrasy ...................... | 2 | 433 |
| Martinet, M. on neuralgia ........ | 19 | 197 |
| Martland, Dr. on melæna.......... | 1 | 450 |
| Marx and Omodei, *v.* Dr. Maclean.. | 3 | 578 |
| Masked cerebral affection, case of. .. | 5 | 584 |
| Masked tertian .................. | 11 | 215 |
| Masked ague, curious case of ...... | 11 | 465 |
| Massachusetts', old laws of on anatomy | 16 | 104 |
| Massachusetts' report on anatomy... | 16 | 106 |
| Massachusetts' new act on anatomy, | 16 | 106 |
| Materia indica, by Dr. Ainslie...... | 6 | 397 |
| Materia medica, Dr. Thompson on .. | 18 | 321 |
| Materia medica, Dr. Thompson's.... | 20 | 403 |
| Materialists *versus* immaterialists.... | 12 | 309 |
| Materialists, arguments against the.. | 14 | 292 |
| Matter forming after dissection-wounds ...................... | 3 | 250 |
| Maxilla inferior; partial excision of, | 6 | 258 |
| Maxillary sinus, disease of the ..... | 11 | 505 |
| Maxwell, Mr. his marvellous case .. | 12 | 498 |
| Maxwell, Dr. on uterine hæmorrhage | 20 | 156 |
| Mayer, M. on the reproduction of the lens ...................... | 19 | 197 |
| Mayo, Mr. his curious case ........ | 10 | 234 |
| Mayo, Mr. on varicose veins....... | 10 | 250 |
| Mayo, Mr. his cases of dislocation of the patella................ | 11 | 145 |
| Mayo, Mr. on ulceration of throat .. | 13 | 265 |
| Mayo, Dr. on temperament........ | 15 | 35 |
| Mayo, Mr. on human physiology.... | 19 | 91 |
| Mayo, Mr. on injuries and diseases of the rectum .............. | 19 | 289 |
| Meals, hours of.................. | 6 | 20 |
| Measles and small-pox, Dr. Stoker.. | 11 | 357 |
| Measles, bad effects of blood-letting in | 11 | 504 |
| Measles, treated by tartar-emetic.... | 19 | 244 |
| Meatus, purulent discharge from the, | 3 | 118 |
| Meatus auditorius, foreign bodies in, | 10 | 573 |
| Mechanical apparatus, Mr. Beale's .. | 13 | 55 |
| Mechanism of parturition, Professor Naegelé on ................. | 11 | 88 |
| Median nerve, partial excision of ... | 17 | 489 |
| Medical miracles................. | 1 | 176 |
| Medical remuneration ............ | 1 | 509 |
| Medical remuneration ............ | 2 | 246 |
| Medical witnesses, their perplexities, | 3 | 172 |
| Medical liberality, anecdote of...... | 3 | 178 |
| Medical essays, by Dr. Marshall Hall, | 4 | 341 |
| Medical etymology, curious samples of | 5 | 303 |
| Medical periodical press, retrospections of the ................. | 6 | 300 |
| Medical literature of the Hindoos... | 6 | 405 |
| Medical examinations and education, | 6 | 583 |
| Medical botany .................. | 7 | 95 |
| Medical science, Dr. Shute's principles of .................... | 7 | 358 |
| Medical cases, reports of; by Dr. Bright ..................... | 8 | 90 |
| Medical Botany, Nos. IV. V. VI. of, | 8 | 117 |
| Medical education, debates on at Guy's................. | 8 | 237 |
| Medical education in Edinburgh .... | 8 | 239 |
| Medical education, inquiry into the state of ................... | 8 | 379 |
| Medical practice, modes of ........ | 9 | 151 |
| Medical conversaziones, present taste for................... | 9 | 165 |
| Medical statistics ................ | 9 | 265 |
| Medical education—march of intellect......................... | 9 | 433 |
| Medical remuneration ............ | 9 | 469 |
| Medical doctrines................ | 9 | 482 |
| Medical treatment of insanity. .... | 10 | 19 |
| Medical essays, Dr. Brown's....... | 10 | 85 |
| Medical evidence in courts of justice, | 10 | 213 |
| Medical examinations ............ | 10 | 241 |
| Medical reporting................ | 10 | 249 |
| Medical degradation.............. | 10 | 493 |
| Medical justice and liberality. ..... | 10 | 498 |
| Medical reform .................. | 10 | 542 |
| Medical societies, reporting from.... | 10 | 543 |
| Medical Calendar, or Student's Guide | 11 | 70 |
| Medical profession, plan for improving the ................. | 11 | 189 |
| Medical reporting from societies.... | 11 | 204 |
| Medical transactions of Calcutta... | 11 | 314 |
| Medical statistics, Dr. Hawkins on.. | 11 | 420 |
| Medical Provident Institution...... | 12 | 484 |
| Medical topography of the Mediterranean ...................... | 13 | 49 |

| | Vol. | Page |
|---|---|---|
| Medical ranks, repletion of the | 13 | 112 |
| Medical department of East India Company | 13 | 112 |
| Medical diploma of London University | 13 | 204 |
| Medical Transactions of Paris | 13 | 567 |
| Medical topography of Ionian Islands | 14 | 97 |
| Medical seed-time | 14 | 145 |
| Medical botany | 14 | 286 |
| Medical practitioners, longevity of | 14 | 319 |
| Medical reform | 14 | 573 |
| Medical zoology and mineralogy | 15 | 126 |
| Medical Provident Institution | 15 | 453 |
| Medical cases, Dr. Bright's | 16 | 1 |
| Medical reports, Dr. Bright's | 16 | 321 |
| Medical constitution | 17 | 285 |
| Medical botany, outline of | 17 | 387 |
| Medical Boards, on the | 19 | 180 |
| Medical jurisprudence | 20 | 120 |
| Medical politics—Aldersgate question | 20 | 185 |
| Medical & Physical Journal, death of, | 20 | 190 |
| Medical reform, remarks on | 20 | 278 |
| Medical topography of Gowhattee | 20 | 363 |
| Medical problems, by Dr. Griffin | 20 | 454 |
| Medical reform | 20 | 560 |
| Medical statistics and reform | 20 | 567 |
| Medicine not a trade | 3 | 177 |
| Medicine, its rank and observance in society | 4 | 127 |
| Medicine *uncertain* no longer | 5 | 21 |
| Medicine, state of, at Naples | 6 | 92 |
| Medicine & surgery, division between | 6 | 569 |
| Medicine and surgery, practice of | 8 | 434 |
| Medicine, popular illustrations of | 12 | 289 |
| Medicine, Dr. Billing's principles of, | 14 | 391 |
| Medicine, histories of | 15 | 394 |
| Medicine, how a science of experience | 16 | 38 |
| Medicine, Cyclopædia of, Part I. | 16 | 445 |
| Med.-Chir. Society of Edinburgh. | 1 | 70 |
| Medico-Chir. Transactions, vol. XII., | 4 | 317 |
| Medico-Chirurgus, his complaint | 5 | 209 |
| Medico-Chirurgical Transactions of Edinburgh | 5 | 392 |
| Medico-Chirurgical Transactions | 6 | 340 |
| Medico-Chirurgical Transactions—review of | 8 | 332, 354 |
| Medico-Chirurgical Transactions | 14 | 49 |
| Medico-Chirurgical Notes. By Mr. Fletcher | 15 | 364 |
| Med.-Chir. Transactions, Vol. VII. | 18 | 33 |
| Medulla spinalis, functions and diseases of | 1 | 2 |
| Medulla spinalis, softening of the | 8 | 535 |
| Medulla, inflammation of | 17 | 179 |
| Medullary and cortical parts of the brain in epilepsy and insanity | 5 | 231 |
| Medullary cancer, or fungus hæmatodes | 11 | 392 |
| Medullary sarcoma of the stomach | 12 | 223 |
| Medullary sarcoma in the ham | 12 | 274 |
| Medusa, the anatomy of | 19 | 232 |
| Melæna, Dr. Chisholm on | 1 | 231 |
| Melæna, Dr. Martland on | 1 | 450 |
| Melæna, discussion on | 8 | 512 |
| Melanosis, Messrs. Cullen & Carswell on | 2 | 154 |
| Melanosis of the stomach; M. Andral, jun. | 5 | 244 |
| Melanosis, Mr. Fawdington's case of, | 5 | 335 |
| Melanosis in horses, researches on | 9 | 534 |
| Melanosis, case of | 14 | 363 |
| Melanosis, Dr. Williams on | 19 | 263 |
| Melted wax for ulcers | 12 | 37 |
| Membrana tympani, puncture of | 1 | 45 |
| Membranes, dropsy of the | 17 | 312 |
| Membranous inflammation of cheek, | 13 | 552 |
| Membranous inflammation, cause of death in | 15 | 477 |
| Mémoires sur la physiologie | 15 | 488 |
| Mémoires de l'Academie Royale de Médecine | 18 | 387 |
| Memorial of the London physicians, | 19 | 192 |
| Memory, loss of | 18 | 548 |
| Memory, loss of, after a gun-shot wound | 19 | 214 |
| Meningea media, aneurism of the. | 10 | 203 |
| Meningea media, wound of the. | 11 | 239 |
| Meningeal apoplexy, Dr. Johnson on, | 1 | 204 |
| Meninges of brain, fungous tumors | 13 | 494 |
| Meningitis, Mr. Davies's case of | 3 | 279 |
| Meningitis, chronic, fatal case of | 4 | 146 |
| Meningitis, Dr. Quain on | 16 | 598 |
| Menses, contraction of the limbs on suppression of | 9 | 151 |
| Menses, absence of the | 10 | 585 |
| Menstrual secretion, nature of | 10 | 99 |
| Menstruation, vicarious; case by Dr. Barnes | 6 | 248 |
| Menstruation in different countries | 17 | 560 |
| Menstruation, Dr. Granville on | 19 | 130 |
| Mental derangement, Dr. Willis on | 2 | 360 |
| Mental alienation, M. Bayle's views of | 4 | 185 |
| Mental alienation, prize essays on. | 5 | 226 |
| Mental diseases, Dr. Voisin on the causes of | 7 | 1 |
| Mental diseases, Dr. Morison on | 9 | 25 |
| Mental disorders, Dr. Uwins on | 13 | 93 |
| Mental derangement affected by the moon | 13 | 485 |
| Mental illusions | 14 | 294 |
| Mental derangement with dissection, | 15 | 217 |
| Mental derangement, Dr. Combe on, | 15 | 564 |
| Mental derangement, Dr. Combe on, | 16 | 423 |
| Mental derangement, always from cerebral disease | 16 | 427 |
| Mental irritation and hypochondriasis | 19 | 343 |
| Mental indigestion, Mr. Fletcher on, | 19 | 344 |
| Merchants and manufacturers, longevity of | 14 | 318 |
| Mercurial ointment, fatal result from, | 1 | 360 |
| Mercurial fumigation, Dr Gibson on, | 3 | 568 |
| Mercurial ointment, new mode of preparing | 3 | 588 |
| Mercurial frictions in puerperal peritonitis | 7 | 201 |

| | Vol. | Page |
|---|---|---|
| Mercurial treatment of fever, Dr. Brown on | 10 | 459 |
| Mercurialization in the plague | 9 | 281 |
| Mercury, in large doses, in syphilis | 1 | 172 |
| Mercury, its efficacy at Millbank | 3 | 12 |
| Mercury, its action on the system | 3 | 438 |
| Mercury and its opponents | 3 | 440 |
| Mercury in the papular venereal | 3 | 451 |
| Mercury in the phagedænic disease | 3 | 469 |
| Mercury in syphilis | 3 | 481 |
| Mercury as a *preventive* of hydrophobia | 3 | 536 |
| Mercury in "dry belly-ache" | 4 | 253 |
| Mercury as a *preventive* of hydrophobia | 4 | 266 |
| Mercury, its physiological effects on the system | 4 | 491 |
| Mercury, its powers in chronic and acute diseases | 5 | 348 |
| Mercury in diptheritis | 5 | 430 |
| Mercury, Medicus on | 6 | 260 |
| Mercury, Dr. Ainslie's opinions on its use | 6 | 399 |
| Mercury, disease of the bones after | 7 | 260 |
| Mercury in "sarcocele" | 9 | 238 |
| Mercury, injection of, producing tubercles | 10 | 81 |
| Mercury in fungus testis | 10 | 465 |
| Mercury, good effects of, in hospital gangrene | 11 | 373 |
| Mercury, diseased testicle cured by | 12 | 231 |
| Mercury in ovarian disease | 12 | 429 |
| Mercury and syphilis, compound of | 13 | 166 |
| Mercury, in traumatic inflammation of brain | 13 | 234 |
| Mercury in the Gibraltar fever | 13 | 348 |
| Mercury in pericarditis | 13 | 431 |
| Mercury, supposed poisoning by | 13 | 472 |
| Mercury in Indian diseases | 15 | 196 |
| Mercury in a case of sloughing ulcer, | 15 | 543 |
| Mercury, paralysis from | 16 | 32 |
| Mercury, delicate tests of | 16 | 379 |
| Mercury in chronic affections of the larynx | 18 | 231 |
| Mercury, use of, in syphilis | 19 | 390 |
| Mercury, effects of | 19 | 449 |
| Mesmerism, Mr. Chevenix on | 10 | 560 |
| Metallic tinkling, Dr. Williams on the | 10 | 581 |
| Metastasis of rheumatism to the brain | 2 | 431 |
| Metastasis, simple *rationale* of | 6 | 323 |
| Meteoration, epidemic | 3 | 85 |
| Metritis, with inflamed veins, a case, | 5 | 242 |
| Metropolitan Society of General Practitioners | 13 | 270 |
| Metropolitan Society, address of the, | 13 | 532 |
| Meyer, Dr. his successful case of Cæsarian section | 4 | 481 |
| Miasm from animal decomposition | 3 | 69 |
| Miasm, distinctions of | 3 | 70 |
| Miasm defined | 3 | 319 |
| Miccoli, Dr. on stibiated mercurial ointment | 1 | 230 |
| Michell, Mr. on the ergot of rye | 9 | 358, 514 |
| Microscopic researches on the blood, | 16 | 256 |
| Microscopical researches in anatomy, | 12 | 51 |
| Middlemore, Mr. on the eye | 13 | 459 |
| Middlemore, Mr. on purulent ophthalmia | 18 | 511 |
| Middlemore, Mr. on the diseases of the eye | 19 | 257 |
| Middlemore, Mr. on ulceration of the eyelid | 20 | 461 |
| Midland hospital reporter | 9 | 286 |
| Midland, Medical & Surgical Journal | 9 | 536 |
| Midwifery, when first practised by a surgeon | 3 | 401 |
| Midwifery, is it to be depised? | 3 | 404 |
| Midwifery, Sir A. Carlisle on | 7 | 587 |
| Midwifery, Dr. King's remarks on | 9 | 214 |
| Midwifery, manual of, by Dr. Ryan, | 17 | 114 |
| Midwifery, Dr. Ramsbotham on | 17 | 289 |
| Midwifery, Dr. Froriep's manual | 19 | 418 |
| Miguel, Dr. on the dangers of iodine, | 2 | 233 |
| Milan—the pellagra | 6 | 99 |
| Miliary fever at Auxi-le-Chateau | 18 | 521 |
| Military surgery, principles of | 12 | 361 |
| Military surgery, Dr. Ballingall's lectures on | 18 | 428 |
| Milk of cows before and after parturition | 17 | 485 |
| Milk, a remedy for dropsy | 18 | 541 |
| Milky blood | 4 | 591 |
| Millard, Mr. on the placenta | 19 | 66 |
| Milldams, imminent dangers of | 8 | 16 |
| Milligan, Dr. on the face and forehead | 10 | 506 |
| Milligan, Dr. on the frontal sinus | 19 | 359 |
| Mills, Dr. on fever and inflammation, | 2 | 14 |
| Mills, Dr. on disorders of the brain | 5 | 350 |
| Mills, Dr. on hydrocephalus | 10 | 28 |
| Mills, Dr. his cases of apoplexy | 12 | 204 |
| Milt-like tumor, Dr. Monro on the | 12 | 265 |
| Mind, its influence on the body | 5 | 17 |
| Mind, influence of the stomach on the | 8 | 121 |
| Mind, effects of the digestive organs on the | 9 | 149 |
| Mind, Dr. Gooch on disorders of the, | 11 | 359 |
| Mind, Dr. Kennedy on | 13 | 146 |
| Mind, progress of disease on the | 14 | 301 |
| Mind, influence of disease on | 15 | 197 |
| Mind, influence of, on the body | 19 | 341 |
| Mind, disorders of, Dr. Uwins on | 20 | 49 |
| Mineral waters of Harrogate, Dr. Hunter on | 15 | 354 |
| Mineral springs, Dr. Gairdner on | 19 | 28 |
| Mineral waters, analysis of | 19 | 338 |
| Miracles of Prince Hohenloe | 1 | 176 |
| Miracles, arguments against | 14 | 298 |
| Misplaced colon, case of | 9 | 573 |
| Misrepresentation, disgraceful system | 8 | 480 |
| Mitchell, Mr. his case of wounded lungs | 5 | 204 |

| | Vol. | Page |
|---|---|---|
| Mitral valve, true action of the .... | 12 | 159 |
| Mitral valve, cases of disease of the.. | 12 | 555 |
| Mitral valves, ossification of ...... | 14 | 567 |
| Modern medical ethics and maxims.. | 12 | 145 |
| Modern works, mode of propagating, | 13 | 314 |
| Modus operandi of the yellow fever miasm .................... | 8 | 87 |
| Mogridge, Mr. on fracture of the patella .................. | 1 | 112 |
| Moir, Mr. on cholera at Musselburgh | 16 | 572 |
| Moles, varieties of ................ | 18 | 421 |
| Mollescence of the lungs, Dr. Hastings on ................ ... | 6 | 517 |
| Mollescence of the uterus, Dr. Luroth on .................... | 9 | 282 |
| Mollescence of the brain ........... | 13 | 500 |
| Mollescence of brain, cases of ...... | 16 | 228 |
| Mollities ossium, a melancholy case of | 5 | 405 |
| Monades, Sir G. Gibbes' speculations on .................... | 8 | 486 |
| Moncrief, Dr. on tuberculated peritoneum .................... | 1 | 302 |
| Monfalcon, M. his Histoire des Marais | 3 | 317 |
| Monomania, homicidal & infanticidal, | 9 | 244 |
| Monomania, infanticidal .......... | 9 | 441 |
| Monomania in a medical man...... | 9 | 507 |
| Monomania—phrenology .......... | 15 | 172 |
| Monomaniacal illusions, samples of.. | 13 | 299 |
| Monro, Dr. his elements of anatomy, | 4 | 52 |
| Monro, Dr. on medullary sarcoma .. | 12 | 224 |
| Monstrosity, curious example of.... | 19 | 246 |
| Monstrosity, anencephalous........ | 19 | 499 |
| Monstrosity by diphlogenesis ...... | 20 | 234 |
| Montegre, M. on hæmorrhoids...... | 2 | 273 |
| Montgomery, Mr. his ligature ultrà aneurisma .................. | 12 | 165 |
| Montgomery, Mr. on carotid aneurism | 12 | 502 |
| Montgomery, Dr. on cancer uteri ... | 14 | 266 |
| Montgomery's, Mr. case of carotid aneurism .................. | 19 | 448 |
| Montgomery, Dr. on pregnancy .... | 20 | 92 |
| Montgomery on moles............ | 20 | 124 |
| Montgomery on the corpora lutea .. | 20 | 127 |
| Moon, influence of, on menstruation, | 18 | 491 |
| Moral causes, their effects upon the heart .... ................ | 3 | 242 |
| Moral agents excluded from medicine! | 5 | 227 |
| Morality among the ancient Romans, | 14 | 460 |
| Morbid appearances after death, from dysentery.............. | 3 | 9 |
| Morbid anatomy and symptomatology | 6 | 54 |
| Morbid sensibility of half the body.. | 8 | 166 |
| Morbid anatomy of the brain, Dr. Monro on . ................ | 8 | 385 |
| Morbid growth in the femur, case of, | 8 | 452 |
| Morbid anatomy of the bowels, &c.. | 9 | 347 |
| Morbid anatomy of the intestines, Dr. Bright on................ | 10 | 321 |
| Morbid phenomena, succession of, in one individual. ............ | 10 | 460 |
| Morbid appearances of the trachea, lungs, and heart, Dr. Mills on the | 11 | 75 |
| Morbid anatomy of the bowels, &c. Dr. Armstrong's ........... | 11 | 97 |
| Morbid anatomy, necessary requisites for...................... | 11 | 367 |
| Morbid anatomy of diabetes........ | 13 | 71 |
| Morbid anatomy, vade-mecum of .. | 15 | 422 |
| Morbid anatomy, considerations on.. | 16 | 46 |
| Morbid anatomy, Dr. Hope's ...... | 18 | 284 |
| Morbid anatomy, works on........ | 18 | 366 |
| Morbid anatomy, Dr. Carswell on .. | 18 | 366 |
| Morbid anatomy, Dr. Hope on ..... | 18 | 371 |
| Morbid anatomy, Dr. Hope on ..... | 20 | 66 |
| Morbid anatomy, Dr. Carswell on .. | 20 | 66 |
| Morbid anatomy, Cruveilhier on.... | 20 | 66 |
| Morbus coxarius, Dr. Ballingall on.. | 10 | 179 |
| Morbus cæruleus, curious case of .. | 18 | 516 |
| Morbus coxarius, curious case of... | 19 | 501 |
| Morgan, Mr. on poisons .......... | 11 | 32 |
| Morgan, Mr. on tetanus .......... | 18 | 536 |
| Moriarty, Sir T. on hepatic phthisis.. | 2 | 184 |
| Morphia, Dr. Bardsley on.......... | 13 | 333 |
| Morphine, salts of................ | 1 | 49 |
| Morphine, experiments on its modus operandi.................. | 11 | 257 |
| Morphine prescribed in mistake, poisoning from . ............ | 11 | 551 |
| Morphine, M. Bally on............ | 12 | 545 |
| Mortal dyspnœa, cases of.......... | 2 | 444 |
| Mortality of children, averaged in London .... .............. | 3 | 483 |
| Mortality in pulmonic inflammation, | 3 | 527 |
| Mortality of young recruits ........ | 17 | 261 |
| Mortality of Paris in 1300 ........ | 18 | 181 |
| Mortality in armies .............. | 20 | 234 |
| Mortality, in different countries .... | 20 | 234 |
| Mortification of the great toe, fatal case of.................... | 6 | 480 |
| Mortification, amputation during its progress ................ | 7 | 219 |
| Mortification from burn ...... ... | 13 | 501 |
| Mortification of the liver.......... | 19 | 443 |
| Morton, Dr. on lactation.......... | 16 | 154 |
| Morton on choleric fever in his time, | 16 | 303 |
| Moscow, cholera in .............. | 16 | 171 |
| Moscow, report of the council of, on cholera... .............. | 16 | 172 |
| Motor linguæ nerve, cases of paralysis of.................... | 6 | 417 |
| Mott, Valentine, his excision of the clavicle .................. | 10 | 549 |
| Mouth, a peculiar affection of...... | 7 | 120 |
| Mouth and fauces, medullary tumor of the........... ...... | 11 | 399 |
| Mouth and fauces, ulcers of the .... | 11 | 402 |
| Moxa, Mr. Boyle on .............. | 2 | 377 |
| Moxa in phlegmatia dolens ........ | 6 | 250 |
| Moxa, Mr. Wallace's work on...... | 6 | 448 |
| Moxa, physiological action of ...... | 6 | 449 |
| Moxa, modes of applying it........ | 6 | 451 |
| Moxa, friction as an adjuvant to.... | 6 | 454 |
| Moxa, sciatica cured by.......... | 8 | 501 |
| Moxa, expeditious................ | 14 | 258 |

| | Vol. | Page |
|---|---|---|
| Moxas for diseases of the heart .... | 13 | 536 |
| Mucous membrane, adhesive inflammation of.............. | 4 | 397 |
| Mucous membrane of the stomach, discoloration of............. | 6 | 147 |
| Mucous membrane, morbid softening of the ................ | 6 | 148 |
| Mucous membrane, morbid growths from the ................. | 6 | 152 |
| Mucous membrane of the stomach.. | 7 | 55 |
| Mucous membrane of the stomach, softening of the............. | 7 | 82 |
| Mucous membrane, inflammation of, | 8 | 361 |
| Mucous membrane of the stomach, &c. natural state of .......... | 10 | 124 |
| Mucous intestinal membrane, diseases of the................ | 11 | 22 |
| Mucous membrane of the lip, great prolongation of ............ | 11 | 566 |
| Mucous membrane of bronchi...... | 13 | 25 |
| Mucous membrane of the rectum... | 14 | 213 |
| Mucous discharge from the uterus.. | 20 | 222 |
| Muller's experiments on spinal nerves | 20 | 225 |
| Muller's experiments upon the blood, | 20 | 501 |
| *Mummification*, Dr. Granville's recipe for .................... | 5 | 50 |
| Mummy, what presented itself on opening one ................ | 5 | 49 |
| Mumps, affecting the testicle ...... | 13 | 399 |
| Murder, curious charge of ........ | 11 | 199 |
| Murder and suicide, propensity to .. | 14 | 254 |
| Muriate of lime, in cases of worms.. | 1 | 226 |
| Muriate of iron in softening of the stomach.................... | 8 | 552 |
| Murray, Mr. on hydrophobia ...... | 13 | 433 |
| Mus musculus, comparative anatomy of.................... | 15 | 572 |
| Muscæ volitantes, pathology of..... | 10 | 512 |
| Muscæ volitantes, Mr. Guthrie on .. | 11 | 184 |
| Muscles, can they produce distortion? | 3 | 368 |
| Muscles, nervous contraction of.... | 3 | 384 |
| Muscles, diseases of .............. | 19 | 510 |
| Muscles, apoplexy of ............ | 19 | 512 |
| Muscles, nervous affections of the .. | 20 | 148 |
| Muscular power, curious instance of, | 3 | 380 |
| Muscular fibres, mode of their contraction ...................... | 9 | 105 |
| Muscular contraction, its causes.... | 15 | 77 |
| Muscular contractions, Dr. Edwards on ...................... | 18 | 211 |
| Muscularity of the urethra ........ | 3 | 164 |
| Museum, Hunterian, observations on | 5 | 635 |
| Musgrave, Dr. his case of abortion.. | 2 | 513 |
| Musgrave, Dr. on "dry belly-ache".. | 4 | 248 |
| Musgrave, Dr. on the effects of mercury.................... | 7 | 423 |
| Musselburgh, cholera at ......... | 16 | 585 |
| Myelitis ......................... | 1 | 33 |
| Myers, Dr. on cynanche trachealis .. | 2 | 181 |
| Myology, Dr. Monro's nomenclature, | 4 | 65 |
| Mysterious case of Henry Hyde, Esq. | 4 | 527 |

| N | Vol. | Page |
|---|---|---|
| Naegelé, Prof. on the mechanism of parturition.................. | 11 | 88 |
| Nævi materni, *vaccination* in ...... | 7 | 280 |
| Nævi materni, Messrs. Lawrence and White on ............... | 8 | 332 |
| Nævi, treatment of .............. | 11 | 232 |
| Nævi materni, M. Biett on......... | 17 | 362 |
| Nævi, treatment of, by seton ...... | 20 | 174 |
| Nævi materni, Graefe's treatment of, | 20 | 219 |
| Nævus maternus, treated by ligature, | 7 | 243 |
| Nævus maternus, Mr. Bennett on .. | 8 | 204 |
| Nævus maternus, cured by vaccination .......... .......... | 9 | 170 |
| Nævus, excision of, from a child 7 weeks old .................. | 11 | 172 |
| Nævus maternus, cured by vaccination .................... | 11 | 269 |
| Nævus, spontaneous ulceration of a.. | 11 | 459 |
| Nævus, subcutaneous, seton for.... | 13 | 506 |
| Nævus, new mode of treating...... | 14 | 480 |
| Nævus, cases of ................ | 15 | 176 |
| Nævus, Dr. Marshall Hall's treatment of.................... | 15 | 177 |
| Nails "growing in," Dupuytren's operation for removing ....... | 4 | 225 |
| Nails, Sir A. Cooper on disease of the | 7 | 181 |
| Nails, diseases of the............. | 14 | 216 |
| Napoleon à Sainte Helene ........ | 10 | 424 |
| Napoleon murdered by the English Government ................ | 10 | 425 |
| Napoleon, account of his illness.... | 10 | 427 |
| Napoleon, dissection of .......... | 10 | 432 |
| Narcotics, externally applied ...... | 17 | 462 |
| Narcotine ...................... | 1 | 50 |
| Nares & antrum, cancerous fungus of the...................... | 11 | 401 |
| National cemetery .............. | 13 | 480 |
| Nature, disease, and the doctor at issue ...................... | 5 | 34 |
| Nautilus, pearly, Mr. Owen on the.. | 18 | 123 |
| Naval surgeons—court etiquette.... | 14 | 570 |
| Naval surgeons, rules affecting .... | 20 | 188 |
| Neale, Mr. on cholera ............ | 16 | 181 |
| Neapolitan hospitals.... ...... .. | 6 | 91 |
| Neck, two cases of tumor removed from....... .............. | 1 | 371 |
| Neck of the bladder, inflammation of, | 8 | 222 |
| Neck of the thigh-bone, interstitial absorption of................ | 10 | 414 |
| Neck, diffuse inflammation of the .. | 17 | 417 |
| Necrosis of the thigh, case of ...... | 20 | 250 |
| Needle swallowed, and taken from the bladder ................ | 16 | 251 |
| Needles, great number extracted from the body ............. | 3 | 559 |
| Negroes, inferiority of the ........ | 19 | 151 |
| Neilgherry, climate of the ........ | 13 | 308 |
| Neilson, Mr. on worms ... ...... | 19 | 168 |
| Nephritis, acute, case of ......... | 18 | 190 |
| Nerve of the tooth, exposed, treatment of .................. | 6 | 213 |

| | Vol. | Page |
|---|---|---|
| Nerve, spiral tumor of | 12 | 184 |
| Nerves, of the teeth, denuded | 1 | 218 |
| Nerves, physiology of | 1 | 437 |
| Nerves of the spine, anatomy of | 2 | 151 |
| Nerves of the spine, inflammation of, Martinet on | 2 | 165 |
| Nerves of motion and sensation, their sizes | 3 | 226 |
| Nerves, divisions of | 6 | 428 |
| Nerves, contusions of | 6 | 431 |
| Nerves, ligature of | 6 | 433 |
| Nerves, re-union of, after division | 6 | 437 |
| Nerves, inflammation of | 6 | 439 |
| Nerves of sense and motion | 7 | 200 |
| Nerves, engravings of the, from Scarpa, &c. | 11 | 108 |
| Nerves, excision of | 12 | 470 |
| Nerves, M. Lizars on division of | 13 | 271 |
| Nerves, Mr. Swan's splendid work on the | 18 | 385 |
| Nerves of sense, paralysis of the | 20 | 213 |
| Nerves, spinal, Muller's experiments, | 20 | 225 |
| Nerves, Mr. Swan on the anatomy of | 20 | 334 |
| Nerves, Mr. Swan on the diseases of, | 20 | 423 |
| Nerves, wounds of the | 20 | 423 |
| Nerves, excision of parts of | 20 | 424 |
| Nerves, tumors in | 20 | 431 |
| Nervous pregnancy | 1 | 495 |
| Nervous pain, hæmorrhoidal | 2 | 282 |
| Nervous injury, a cause of idiotcy | 2 | 469 |
| Nervous pains, datura stramonium in | 2 | 492 |
| Nervous disorders combined with dysentery | 3 | 16 |
| Nervous disorders cured by mercury, | 3 | 20 |
| Nervous affections of the heart, M. Laennec on | 8 | 428 |
| Nervous system | 12 | 22 |
| Nervous temperament | 13 | 17 |
| Nervous diseases, treatment of | 13 | 91 |
| Nervous irritation, effects of | 13 | 328 |
| Nervous influence, electricity only | 15 | 18 |
| Nervous system of plants | 17 | 391 |
| Nervous disturbances, on | 19 | 341 |
| Nervous affections of the muscles | 20 | 148 |
| Neuralgia, Dr. Uwins on | 1 | 135 |
| Neuralgia, simulating brain disease | 1 | 186 |
| Neuralgia, Dr. Evans on | 1 | 198 |
| Neuralgia, Dr. Borthwick on | 1 | 199 |
| Neuralgia of the scalp cured by operation | 5 | 295 |
| Neuralgia, Dr. Trevor on | 5 | 535 |
| Neuralgia, a curious case of | 5 | 612 |
| Neuralgia spasmodica, Dr. Scudamore on | 7 | 355 |
| Neuralgia, pathology of | 9 | 185 |
| Neuralgia, stramonium in | 9 | 282 |
| Neuralgia, visceral, M. Jolly on | 9 | 333 |
| Neuralgia, distressing case of | 9 | 435 |
| Neuralgia, remarkable case of | 9 | 481 |
| Neuralgia, Dr. Macculloch on | 10 | 49 |
| Neuralgia from injuries | 10 | 62 |
| Neuralgia, remarks on the medical case of | 10 | 212 |
| Neuralgia, Dr. Macculloch on | 10 | 357 |
| Neuralgia ophthalmica—rheumatism of the eye | 10 | 357 |
| Neuralgia and intermittent, connexion between | 10 | 365 |
| Neuralgia, cure of | 10 | 368 |
| Neuralgia, oil of turpentine for | 10 | 505 |
| Neuralgia, curious case of | 10 | 553 |
| Neuralgia of the genito-urinary organs | 11 | 276 |
| Neuralgia, Mr. Bell on | 11 | 417 |
| Neuralgia, some cases of, by Dr. Passagay | 11 | 538 |
| Neuralgia of the heart | 12 | 122 |
| Neuralgia of the stomach | 12 | 125 |
| Neuralgia, Dr. Palmer on | 12 | 310 |
| Neuralgia, from uterine irritation | 13 | 203 |
| Neuralgia of the diaphragm | 13 | 461 |
| Neuralgia, and tumors of nerves | 14 | 161 |
| Neuralgia of the heart, Dr. Elliotson on | 15 | 212 |
| Neuralgia, Dr. Bright on | 16 | 332 |
| Neuralgia of the mammæ | 17 | 461 |
| Neuralgia, cyanuret of potassium in, | 18 | 525 |
| Neuralgia of the testis | 19 | 174 |
| Neuralgia cured by animal magnetism | 19 | 184 |
| Neuralgia, treatment of, by Martinet, | 19 | 197 |
| Neuralgia cured by quinine and morphia | 19 | 217 |
| Neuralgia, Dr. Halliday on | 19 | 421 |
| Neuralgia treated with belladonna | 19 | 503 |
| Neuralgiæ, cases of, cured by acupuncture | 3 | 258 |
| Neuralgiæ, cases of, treated by ditto, | 3 | 262 |
| Neuralgiæ of various parts | 6 | 442 |
| Neuralgiæ, malarious origin of | 9 | 53 |
| Neuralgiæ, in various parts of the body | 10 | 55 |
| Neuralgic diseases, Mr. Teale on | 12 | 112 |
| Neuralgic rheumatism, case of | 15 | 216 |
| Neuralgic affection of stumps | 20 | 555 |
| Neuritis, in a hydrophobic patient | 3 | 534 |
| Neuroses, do they depend on inflammation? | 6 | 324 |
| Neurosis, M. Barras' theory of the | 8 | 51 |
| New remedies, preparation of | 1 | 47 |
| New Pharmacopœia | 1 | 56 |
| New medical logic, Vincenzo Lanza's | 5 | 128, 470 |
| New-nose-making, death from | 9 | 563 |
| Newburn, cholera at | 17 | 121 |
| Newburn, situation of | 17 | 122 |
| Newburn, proportion of population attacked | 17 | 127 |
| Newnham, Mr. on green tea | 7 | 143 |
| Newstead, Mr. on affections simulating inflammations | 11 | 478 |
| Nice, climate of | 11 | 134 |
| Nicholls, Dr. his case of tetanus | 3 | 283 |
| Nimmo, Mr. on restoration of vision, | 19 | 156 |
| Nisbet *versus* Parsons | 1 | 243 |
| Nitras argenti for aphonia | 17 | 435 |
| Nitrate of potash in dropsies | 2 | 531 |

| | Vol. | Page |
|---|---|---|
| Nitrate of silver, history and properties of | 3 | 160 |
| Nitrate of silver, antidote to ..... | 3 | 161 |
| Nitrate of silver, an application in croup | 4 | 261 |
| Nitrate of silver in epilepsy ........ | 6 | 241 |
| Nitrate of potass in indigestion .... | 7 | 376 |
| Nitrate of silver in affections of the eye | 9 | 182 |
| Nitrate of silver in diseases of the eye, | 10 | 225 |
| Nitrate of silver, Mr. Higginbottom on | 10 | 444 |
| Nitrate of silver, Mr. Ceely on ..... | 11 | 496 |
| Nitrate of silver in leucorrhœa..... | 12 | 517 |
| Nitrate of silver in cynanche laryngea | 20 | 155 |
| Nitre extracted from the stomach .. | 2 | 487 |
| Nitre in menorrhagia ............ | 3 | 298 |
| Nitre in scurvy, Mr. Cameron on ... | 12 | 483 |
| Nitric acid, dilute, injected into the bladder | 15 | 259 |
| Nitric acid in toothache .......... | 16 | 578 |
| Nitric acid for toothache .......... | 17 | 537 |
| Nitric acid, poisoning from........ | 19 | 546 |
| Nitric acid, effects of a large dose of, | 20 | 64 |
| Nitro-muriatic bath, directions for using | 10 | 318 |
| Nitrous acid and opium, in dysentery | 5 | 580 |
| Nitrous oxyde gas, properties of the, | 16 | 377 |
| Nodes, unfrequent in the papular venereal | 3 | 452 |
| Nodes in phagedæna............... | 3 | 469 |
| Nodes, treatment of............. | 3 | 475 |
| Non-jurors .................... | 6 | 270 |
| Non-union of fractures............ | 10 | 568 |
| Non-united fractures, treated by iodine | 10 | 132 |
| North, Mr. on peculiar spasms in children | 2 | 452 |
| North, Mr. on convulsions of infants, | 5 | 149 |
| Norwich Medical School .......... | 14 | 248 |
| Nose, loss of, in Russia........... | 5 | 523 |
| Nose, affections of the septum...... | 19 | 454 |
| Nostalgia, M. Larrey on .......... | 15 | 18 |
| Note takers, rival............... | 12 | 193 |
| Nothing new under the sun........ | 12 | 247 |
| Nothing new under the sun........ | 14 | 467 |
| Noxious exhalations, influence of .. | 7 | 280 |
| Nugæ Canoræ .................. | 7 | 38 |
| Numann's experiments on small-pox, | 20 | 208 |
| Nuttall, Dr. the late.............. | 14 | 286 |
| Nux vomica, resin of............. | 1 | 49 |
| Nux vomica, paraplegia cured by ... | 12 | 203 |
| Nux vomica, for amaurosis........ | 13 | 441 |
| Nux vomica in paraplegia ........ | 19 | 487 |
| Nymphomania and satyriasis ..... | 7 | 16 |
| Nymphomania, Dr. D. Davis on .... | 19 | 465 |
| Nymphomania, Dr. Bright on...... | 19 | 468 |

### O

| | Vol. | Page |
|---|---|---|
| O'Beirne, Dr. on defecation ....... | 19 | 1 |
| O'Beirne, Dr. on dysentery........ | 19 | 22 |
| O'Beirne, Dr. on delirium tremens.. | 19 | 26 |
| Obligation of the practitioner to attend inquests | 3 | 171 |
| Obliteration of the urethra ........ | 1 | 215 |
| Obliteration of the bile-ducts, cases of | 3 | 237 |
| Obliteration of the abdominal aorta, cases of | 9 | 210 |
| Obliteration of inferior cava ...... | 13 | 550 |
| Obliteration of inferior cava ....... | 15 | 569 |
| O'Brien, Dr. on sulphate of quinine, | 2 | 227 |
| O'Brien, Dr. his report on fever .... | 13 | 403 |
| Obscure peritonitis, cases of....... | 12 | 478 |
| Observations on a species of premature labour | 1 | 355 |
| Observations on medicine, by the late Dr. Baillie | 4 | 364 |
| Observations in surgery & pathology, | 17 | 43 |
| Obstetric Society; notice of it...... | 4 | 302 |
| Obstetric Society, proceedings of the | 13 | 241 |
| Obstetric medicine, Dr. Davis on.... | 16 | 235, 579 |
| Obstetric medicine, Dr. Davis's .... | 18 | 491 |
| Obstetric auscultation, Dr. Kennedy, | 20 | 92 |
| Obstetrical bandage, Mr. Gaitskell's, | 9 | 177 |
| Obstinate vomiting, cured by extract of marigold | 5 | 197 |
| Obstructed menses, remedy for..... | 6 | 303 |
| Obstructed and inflamed hernia, Mr. Stephens on | 11 | 109 |
| Obstruction of the colon .......... | 1 | 125 |
| Obstruction of arteries, M. Bally on, | 7 | 218 |
| Obstruction of the cava and iliac veins | 14 | 206 |
| Occipito-frontalis; diffuse inflammation | 14 | 183 |
| Occlusion of intestinal and urinary passages | 1 | 438 |
| Occult disease of chest—Dr. Carter.. | 2 | 161 |
| O'Connor, Dr. on the *bische* of Trinidad | 6 | 470 |
| Ocular illusions, Sir David Brewster on | 17 | 467 |
| Ocular illusions, instances of ...... | 17 | 469 |
| Ocular illusions, other instances of.. | 17 | 478 |
| Odontoid process, caries of the .... | 12 | 489 |
| Odorous principle in the blood of man | 11 | 483 |
| Odour, peculiar, of insane persons .. | 10 | 15 |
| Œdemato-phlegmonous neuritis .... | 2 | 525 |
| Œdematode swelling of the leg, curious case | 8 | 450 |
| Œsophagotomy, cases of......... | 19 | 239 |
| Œsophagotomy, Mr. Arnott's case of, | 20 | 172 |
| Œsophagus, constriction of, in hysteria | 4 | 35 |
| Œsophagus, curious case of stricture of the | 5 | 294 |
| Œsophagus, stricture of the........ | 10 | 119 |
| Œsophagus, Mr. Macilwain on ..... | 13 | 200 |
| Œsophagus and trachea, section of.. | 13 | 246 |
| Œsophagus, foreign bodies in...... | 14 | 565 |
| Œsophagus, Mr. Fletcher on strictures of | 15 | 387 |
| Œsophagus, diseases of the........ | 15 | 423 |
| Œsophagus, malformations of the .. | 20 | 230 |
| Œsophagus, rupture of the ........ | 20 | 230 |
| Œsophagus, dilatation of the ...... | 20 | 230 |

| | Vol. | Page |
|---|---|---|
| Ogden, Dr. on cholera at Sunderland | 16 | 533 |
| O'Halloran, Dr. on dysentery ...... | 2 | 234 |
| O'Halloran, Dr. on ophthalmia..... | 2 | 305 |
| O'Halloran's letter on cholera.. . . | 16 | 608 |
| Oil of bitter almonds, its nature, &c. | 3 | 148 |
| Oil in the blood, Dr. Adam's case of, | 5 | 39 |
| Oil of turpentine in iritis.......... | 11 | 525 |
| Oil of turpentine, Dr. Moran on the, | 12 | 211 |
| Oil of turpentine in iritis.......... | 12 | 232 |
| Oil in the blood, Dr. Babington on.. | 14 | 208 |
| Ointments, mercurial and iodidic... | 3 | 353 |
| Old people, on their diseases....... | 3 | 571 |
| Olecranon, fractures of....... .... | 1 | 65 |
| Olecranon, Mr. Alcock on fractures of the.............. .... | 1 | 117 |
| Olfactories *not* the nerves of smell.. | 4 | 303 |
| Ollivier, Dr. his treatise on spinal diseases ...................... | 1 | 1 |
| Omentum, disease of the.......... | 13 | 225 |
| Omission of care to the new-born child....................... | 1 | 349 |
| Omission of ligature to the funis.... | 1 | 350 |
| Onanism, effects of, on the heart... | 9 | 147 |
| Opacities of the cornea, their treatment ...................... | 3 | 359 |
| Opacities of the cornea, Dupuytren's panacea for............ | 4 | 229 |
| Open foramen ovale, Dr. Crampton's case.................... | 9 | 249 |
| Open foramen ovale, cases of . .... | 11 | 230 |
| Operation of extirpating parotid gland | 1 | 216 |
| Operation, sudden death in........ | 2 | 218 |
| Operation for hepatic abscess ... .. | 10 | 439 |
| Operation for pneumo-thorax...... | 10 | 478 |
| Operation for aural aneurism...... | 15 | 472 |
| Operation, Cæsarian, case of ...... | 20 | 207, 221 |
| Operations, bloody, remarks on .... | 3 | 267 |
| Operations at St. Thomas's hospital, | 6 | 617 |
| Operations on wounded arteries . .. | 13 | 137 |
| Operations for internal strangulations | 13 | 544 |
| Operations, some rules respecting .. | 18 | 396 |
| Operative midwifery, Dr. Davison .. | 4 | 148 |
| Operative surgery, M. Velpeau's.... | 18 | 395 |
| Operatives, miseries and vices of.... | 16 | 80 |
| Ophthalmia, Mr. Ryall on......... | 2 | 220 |
| Ophthalmia, Dr. O'Halloran on .... | 2 | 305 |
| Ophthalmia, Mr. Melin on ........ | 2 | 470 |
| Ophthalmia, Mr. Mackenzie on..... | 6 | 173 |
| Ophthalmia porriginosa, Mr. Christian on.................... | 8 | 531 |
| Ophthalmia in the East .......... | 11 | 327 |
| Ophthalmia, acute purulent........ | 12 | 196 |
| Ophthalmia, intermittent.......... | 12 | 534 |
| Ophthalmia, gonorrhœal, acute .... | 14 | 82 |
| Ophthalmia, gonorrhœal, cases of .. | 14 | 91 |
| Ophthalmia, catarrhal ........... | 14 | 401 |
| Ophthalmia, contagious .. ....... | 14 | 403 |
| Ophthalmia, neonatorum........... | 14 | 407 |
| Ophthalmia, scrofulous ........... | 14 | 409 |
| Ophthalmia, erysipelatous ........ | 14 | 413 |
| Ophthalmia, variolous ............ | 14 | 414 |
| Ophthalmia, morbillous and scarlatinous ......................... | 14 | 416 |
| Ophthalmia, rheumatic ........... | 14 | 417 |
| Ophthalmia, catarrho-rheumatic.... | 14 | 419 |
| Ophthalmia, catarrhal, cases of .... | 17 | 267 |
| Ophthalmia, purulent, cases of .... | 17 | 269 |
| Ophthalmia, strumous, cases of.... | 17 | 273 |
| Ophthalmia, Egyptian, cure of .... | 19 | 221 |
| Ophthalmia, purulent, Mr. Lawrence | 20 | 43 |
| Ophthalmia, strumous............ | 20 | 44 |
| Ophthalmiæ in general............ | 14 | 397 |
| Ophthalmiæ, treatment of the...... | 14 | 399 |
| Ophthalmiæ, traumatic .......... | 15 | 73 |
| Ophthalmiæ, compound........... | 15 | 74 |
| Ophthalmiæ, intermittent ........ | 15 | 74 |
| Ophthalmic surgery in France, quackery of.................. | 4 | 226 |
| Opiates in convulsions, Mr. North's remarks on ................. | 5 | 160 |
| Opium in acute inflammation ...... | 1 | 118 |
| Opium, Mr. Battley's analysis of.... | 1 | 253, 521 |
| Opium, as a poison .............. | 1 | 326 |
| Opium, poisoning by............. | 2 | 235 |
| Opium in puerperal irritability ..... | 2 | 504 |
| Opium, its efficacy in cholera ...... | 3 | 137 |
| Opium, its powers................ | 3 | 154 |
| Opium, cautions respecting its employment.................... | 3 | 156 |
| Opium, too much dreaded in inflammations ................ | 3 | 223 |
| Opium in the delirium traumaticum, | 10 | 525 |
| Opium eaters in Turkey .......... | 11 | 143 |
| Opium in infantile diseases........ | 12 | 553 |
| Opium, immense doses of, in hysteria | 13 | 564 |
| Opium in uterine hæmorrhage...... | 17 | 339 |
| Opium, Dr. Stokes on large doses of, | 18 | 156 |
| Opium, effects of, in enemata...... | 19 | 148 |
| Oppenheim, Dr. on Turkish medicine | 19 | 435 |
| Optic nerves of the simia aygula.... | 3 | 203 |
| Optic nerve, neuralgia of.......... | 10 | 56 |
| Optic nerves, anatomy & physiology of | 17 | 447 |
| Orbit, tumors in the.............. | 9 | 253 |
| Orbit, wound of the.............. | 20 | 253 |
| Orchitis, Mr. Russell on .......... | 19 | 172 |
| Ore, Mr. his case of extraordinary pulmonary abscess ........... | 20 | 192 |
| O'Reardon, Dr. on fever .......... | 10 | 259 |
| O'Reardon, Dr. on fever .......... | 11 | 181 |
| Orfila, M. on the signs of drowning, | 9 | 489 |
| Orfila on poisoning by mercury .... | 13 | 472 |
| Organic disease, what is it?........ | 3 | 92 |
| Organic diseases of the stomach .... | 10 | 336 |
| Orthopedia ..................... | 6 | 258 |
| Orthopedy, treatment of .......... | 19 | 504 |
| Orton, Mr. on cholera............ | 16 | 193 |
| Orton, Mr. his theory of cholera.... | 16 | 486 |
| Os uteri, M. Guilbert on the leeching of the ................ | 5 | 508 |
| Os uteri, imperforate, operation for, | 13 | 553 |
| Os tincæ, ulcers of ............. | 19 | 522 |
| Os tincæ, granular inflammation of.. | 19 | 524 |

| | Vol. | Page |
|---|---|---|
| O'Shaughnessy, Dr. on poisoned confectionery | 15 | 201 |
| O'Shaughnessy, Dr. his translation of Lugol | 16 | 109 |
| O'Shaughnessy's analysis of cholera secretions | 16 | 496 |
| Ossific testis | 13 | 406 |
| Ossification of the coronary arteries, | 9 | 158 |
| Osteo-sarcoma of the jaw, Mr. Fereday | 7 | 565 |
| Osteo-sarcoma, excision of the clavicle for | 10 | 549 |
| Osteo-sarcoma, removal of | 12 | 469 |
| Osteo-sarcoma of the arm | 18 | 166 |
| Osteology, Dr. Witt on | 20 | 149 |
| Otic ganglion, Arnold's | 20 | 498 |
| Otitis, Dr. Kennedy on | 4 | 591 |
| Otitis, external, in the insane | 20 | 201 |
| Otorrhœa purulenta, Dr. Burne on | 11 | 538 |
| Otorrhœa purulenta, treatment of | 11 | 541 |
| Otto's pathological anatomy | 17 | 427 |
| Otto, Dr. on small-pox & vaccination | 19 | 481 |
| Ova in mammiferous animals | 20 | 509 |
| Ovaria, extirpation of | 2 | 215 |
| Ovaria, extraction of, considered | 3 | 344 |
| Ovaria, diseased, Mr. Lizars on | 3 | 335 |
| Ovaria, case of extraction of | 3 | 337 |
| Ovaria, operations on, in Kentucky | 5 | 620 |
| Ovaria, structural diseases of the | 12 | 417 |
| Ovaria, cysts of the | 12 | 419 |
| Ovaria, scirrhus of the | 12 | 421 |
| Ovaria, fungoid disease of the | 12 | 422 |
| Ovaria, cases of extirpation of the | 12 | 440 |
| Ovaria, the seat of the soul | 12 | 462 |
| Ovaria, dropsical, spontaneous cures of | 20 | 206 |
| Ovarian dropsy, old operations for | 3 | 588 |
| Ovarian dropsy, its fatality | 4 | 418 |
| Ovarian disease, rapid development of | 11 | 258 |
| Ovarian disease, treatment of | 12 | 426 |
| Ovarian tumors, etiology of | 13 | 47 |
| Ovarian disease, remarkable case of, | 14 | 552 |
| Ovarian tumor injected | 15 | 501 |
| Ovarian disease complicated with pregnancy | 18 | 46 |
| Ovarian tumor, case of | 20 | 177 |
| Ovarium, extirpated, case of | 3 | 341 |
| Ovarium, attempted extraction of | 3 | 342 |
| Ovary, encysted tumor of, cured | 10 | 467 |
| Ovary, extirpation of | 19 | 242 |
| Ovum, blighted, case of | 1 | 124 |
| Ovum, blighted, Mr. Powell's case | 1 | 131 |
| Owen, Mr. on the placenta | 19 | 65 |
| Oxalic acid, tests for | 12 | 496 |
| Oxalic acid, distinguished from Epsom salts | 16 | 379 |
| Oxford "Traveller-ships;" their *important* results | 6 | 89 |
| Oxford *v.* Cambridge—a college *tale*, | 9 | 523 |
| Oxymurias hydrargyri (test for) | 1 | 324 |

| P | Vol. | Page |
|---|---|---|
| Paganini, organization of | 17 | 177 |
| Paget, Dr. on malformations of heart | 16 | 68 |
| Paillard's account of the siege of Antwerp | 18 | 578 |
| Pain, effect of, on the powers of the heart | 4 | 528 |
| Pain in the side, hysterical | 12 | 292 |
| Pain in the side and spine in hysteria, | 13 | 36 |
| Paine, Dr. on cerebral diseases | 20 | 433 |
| Painful affections of the stomach | 4 | 41 |
| Painful swellings of lower extremities, | 14 | 496 |
| Pains, hæmorrhoidal | 2 | 281 |
| Pains, sympathetic, in uterine irritation | 13 | 41 |
| Pains, spasmodic, treatment of | 19 | 179 |
| Palmer, Dr. his popular medicine | 12 | 289 |
| Palpitatio cordis, digitalis in | 5 | 277 |
| Palpitation of the heart, M. Laennec on | 9 | 20 |
| Palpitations of the heart | 4 | 370 |
| Palsy, facial, use of phosphorus in | 20 | 204 |
| Panacea, universal, for all complaints | 9 | 456 |
| Pancreas, pathology of the | 10 | 353 |
| Pancreas, disease of the | 13 | 227 |
| Pancreas, inflammation of | 15 | 502 |
| Pancreas, diseases of, Dr. Bright on, | 20 | 1 |
| Panic, the cause of fever | 2 | 148 |
| Papillæ of the tongue, Dr. Ravin on, | 5 | 288 |
| Papular venereal disease, account of, | 3 | 447 |
| Papular eruption and mercury | 3 | 458 |
| Par vagum, M. Breschet's memoir. | 3 | 200 |
| Par vagum, neuralgia of the | 10 | 553 |
| Par vagum, Mr. Swan's experiments on | 20 | 425 |
| Par vagum, supposed inflammation of | 20 | 428 |
| Par vagum, pressure on the | 20 | 430 |
| Paracentesis capitis | 1 | 214 |
| Paracentesis thoracis | 2 | 96, 213 |
| Paracentesis capitis, Mr. Money's case of | 2 | 478 |
| Paracentesis thoracis, Morand's case, | 2 | 480 |
| Paracentesis thoracis, successful case, | 3 | 219 |
| Paracentesis thoracis, Dr. Jackson's case | 3 | 542 |
| Paracentesis successful in empyema, | 5 | 267 |
| Paracentesis thoracis, American case | 5 | 582 |
| Paracentesis thoracis, a successful case of | 6 | 78 |
| Paracentesis thoracis, successful case | 7 | 273 |
| Paracentesis abdominis, during pregnancy | 7 | 409 |
| Paracentesis abdominis through the bladder | 9 | 224 |
| Paracentesis thoracis | 12 | 468 |
| Paracentesis thoracis, for empyema | 13 | 449 |
| Paracentesis thoracis for hydrothorax, | 15 | 149 |
| Paracentesis abdominis, case of | 20 | 177 |
| Paralysis, not an usual effect of mercury | 1 | 238 |
| Paralysis on the same side as the injury | 1 | 446 |

| | Vol. | Page |
|---|---|---|
| Paralysis from a fall, case of ....... | 3 | 58 |
| Paralysis of the portio dura ........ | 3 | 203 |
| Paralysis of the portio dura, Dr. Delafield's cases ............. | 3 | 281 |
| Paralysis, iodine in .............. | 4 | 7 |
| Paralysis, some rare cases of ...... | 5 | 602 |
| Paralysis of organs, some curious cases of .................... | 6 | 69 |
| Paralysis attendant on insanity, Dr. Chalmeil on ............... | 6 | 363 |
| Paralysis, pathology of this disease.. | 6 | 365 |
| Paralysis, its progress & termination, | 6 | 370 |
| Paralysis, local, cases of .......... | 6 | 446 |
| Paralysis, from contusion of the spine | 8 | 203 |
| Paralysis of the left side of the body, case of ..................... | 8 | 558 |
| Paralysis with apoplexy .......... | 9 | 79 |
| Paralysis cured by lightning........ | 9 | 456 |
| Paralysis and headache from intestinal worms................. | 11 | 563 |
| Paralysis, strychnia in ............ | 12 | 130 |
| Paralysis, ascending, curious case of, | 12 | 168 |
| Paralysis, dorsal vertebræ removed.. | 12 | 207 |
| Paralysis agitans, remarks on, and case of.................. | 14 | 167 |
| Paralysis agitans, Mr. Parkinson's cases of ................... | 14 | 170 |
| Paralysis, Dr. Darwall on a species of, | 14 | 249 |
| Paralysis of lower extremities, case of | 15 | 519 |
| Paralysis from lead............... | 16 | 29 |
| Paralysis from mercury ........... | 16 | 32 |
| Paralysis aped by hysteria.......... | 16 | 326 |
| Paralysis of portio dura, cured by moxa ...................... | 17 | 437 |
| Paralysis of the nerves of seeing, hearing, and smelling ......... | 20 | 213 |
| Paraphymosis, cases of............ | 15 | 236 |
| Paraplegia spontanea, instance of ... | 8 | 192 |
| Paraplegia, Mr. Earle on .......... | 8 | 354 |
| Paraplegia, Dr. Thomas on.. ....... | 8 | 447 |
| Paraplegia, use of strychnine in .... | 10 | 164 |
| Paraplegia, case of............... | 11 | 162 |
| Paraplegia cured by nux vomica.... | 12 | 203 |
| Paraplegia, cured with nux vomica.. | 19 | 487 |
| Paris, Dr. on poisons ............ | 1 | 316 |
| Paris, Dr. on infanticide .......... | 1 | 337 |
| Paris, Dr. on diet................ | 5 | 636 |
| Paris, Dr. his treatise on diet ... .. | 6 | 1 |
| Paris, lectures, &c. delivered at. .... | 11 | 72 |
| Paris, Dr. his life of Davy ......... | 14 | 423 |
| Paris, bill of mortality in, for 1830.. | 18 | 181 |
| Paris, insalubrity of.............. | 19 | 241 |
| Parisian hospitals, Dr. Ratier on the, | 3 | 94 |
| Parisian epidemic .......... .. .. | 10 | 156 |
| Parisian hospitals, formulary of the, | 12 | 540 |
| Parisian treatment of intermittents.. | 13 | 154 |
| Parisian epidemic, description of the, | 14 | 200 |
| Parkinson, Mr. new treatment of typhus ..... ............. | 1 | 229 |
| Parotid gland, extirpation of ...... | 1 | 216 |
| Parotid gland, case of *extirpation* of, | 4 | 546 |
| Parotid, scirrhous, extirpation of a,. | 5 | 223 |
| Parotid gland, extirpation of ....... | 6 | 564 |
| Parotid duct, fistula of the ........ | 9 | 473 |
| Parotid fistula, M. Roux's mode of treating ...................... | 11 | 443 |
| Parotid tumor, extirpation of a. .... | 16 | 603 |
| Parricide, instructive case of ....... | 6 | 486 |
| Parry, Dr. his elements of pathology, | 3 | 188 |
| Parry, Caleb, his posthumous works, | 4 | 124 |
| Partial fracture of the femur ...... | 5 | 249 |
| Partial ague .................... | 9 | 438 |
| Partial palsy cured by strychnine. .. | 11 | 272 |
| Partial ramollissement of the brain.. | 12 | 215 |
| Parturition, sudden cases of........ | 1 | 237 |
| Parturition, exhaustion in, case of .. | 3 | 411 |
| Parturition, difficult case of........ | 5 | 198 |
| Parturition, Mr. Ashwell on ....... | 10 | 97 |
| Parturition, affections of the joints.. | 12 | 33 |
| Paruria erratica, extraordinary case.. | 8 | 509 |
| Patella, fractures of .............. | 1 | 112 |
| Patella, rupture of its ligament .... | 4 | 239 |
| Patella, fracture of the............ | 6 | 299 |
| Patella, remarkable alteration of the, | 10 | 556 |
| Patella dislocated outwards, curious cases of .................. | 11 | 145 |
| Patella, Sir C. Bell on fracture of ... | 18 | 482 |
| Patent Vituperative Company...... | 6 | 270 |
| Paterson, Dr. on asphyxia ........ | 19 | 265 |
| Paterson, Dr. on amenorrhœa...... | 20 | 171 |
| Pathological action of marsh effluvium | 3 | 322 |
| Pathological propositions.......... | 6 | 321 |
| Pathological observations, by Dr. Horner .................... | 9 | 211 |
| Pathological researches, Dr. Abercrombie's..................... | 11 | 1 |
| Pathological observations, Dr. Stoker's.................. .... | 11 | 337 |
| Pathological anatomy, remarks on .. | 13 | 367 |
| Pathological observations —— Dr. Houston's .................... | 14 | 344 |
| Pathological anatomy, Andral's .... | 14 | 434 |
| Pathological waltz, Dr. Badham's. .. | 15 | 277 |
| Pathological anatomy of Otto ...... | 17 | 427 |
| Pathological anatomy, works on.... | 20 | 289 |
| Pathology of hooping-cough ....... | 1 | 192 |
| Pathology of the peritoneum ...... | 1 | 199 |
| Pathology of the fluids, Dr. Stoker.. | 1 | 144 |
| Pathology of scrofula, Dr. Alison on, | 2 | 99 |
| Pathology of epilepsy, Dr. Reid .... | 2 | 205 |
| Pathology, definition of .......... | 3 | 89 |
| Pathology of delirium tremens..... | 4 | 38 |
| Pathology of Indian cholera........ | 4 | 91 |
| Pathology of "compact œdema".... | 4 | 206 |
| Pathology of fever ............... | 5 | 11 |
| Pathology of fever, Dr. Hewett on the | 7 | 494 |
| Pathology of the blood, Dr. Clanny on | 8 | 510 |
| Pathology of fever ............... | 9 | 130 |
| Pathology of muscæ volitantes...... | 10 | 512 |
| Pathology of the stomach.......... | 13 | 223 |
| Pathology of hooping-cough........ | 14 | 142 |
| Pathology, outlines of, by Dr. Alison | 19 | 306 |
| Patients, their habits to be considered | 4 | 106 |
| Pattison, Prof. & London University, | 15 | 330 |

| | Vol. | Page |
|---|---|---|
| Pau, climate of | 11 | 131 |
| Paul, Dr. case of amputation in an infant | 20 | 191 |
| Payne, Dr. on puerperal fever | 2 | 243 |
| Pearly nautilus, Mr. Owen on the | 18 | 123 |
| Pearson, Dr. Burton, on brain fever, | 13 | 198 |
| Pecchioli, Dr. his lithontripteur | 10 | 191 |
| Pellagra of Lombardy | 14 | 453 |
| Pellagra, notice of | 18 | 176 |
| Pellagra, cases of | 18 | 178 |
| Pellagra, Dr. Chiappa on | 19 | 507 |
| Pelvic tumors during parturition | 3 | 421 |
| Pelvis, deformed, remarks on | 3 | 423 |
| Pelvis, measurement of | 4 | 59 |
| Pelvis, fracture of | 7 | 276 |
| Pelvis, fractured, case of | 17 | 535 |
| Pelvis, fractured, fatal case of | 17 | 539 |
| Pemphigoid affection of the spleen | 8 | 216 |
| Pemphigus, M. Biett on | 16 | 394 |
| Penetrating wounds of the chest, cases of | 3 | 314 |
| Penis, cancer of | 7 | 243 |
| Penis, suicidal amputation of the | 10 | 539 |
| Penis, amputation of the | 12 | 277 |
| Penis, sloughing of | 13 | 436 |
| Penis, injury and amputation of | 18 | 223 |
| Penis, abscess of the | 19 | 559 |
| Penitentiary, Dr. Latham's account of its endemic | 3 | 1 |
| Percussion, Avenbrugger, &c. on | 2 | 65 |
| Percussion and the stethoscope, Dr. Ryland on | 4 | 449 |
| Percussion and pressure in ovarian tumors | 12 | 431 |
| Percussion of the lungs | 20 | 339 |
| Percussion of the heart | 20 | 343 |
| Percussion of the abdomen | 20 | 345 |
| Percussion of the liver | 20 | 346 |
| Pères de la Charité, their curious treatment of colica pictonum | 3 | 364 |
| Perforation of the intestines | 2 | 182 |
| Perforation of the stomach, case of | 3 | 221 |
| Perforation of the stomach, curious case of | 4 | 516 |
| Perforation and softening of the stomach | 6 | 240 |
| Perforation of the stomach and diaphragm | 10 | 221 |
| Perforation of the stomach, case of | 11 | 496 |
| Perforation of the intestine in fever | 13 | 224 |
| Perforations of the stomach, Dr. Gairdner on | 2 | 170 |
| Perforations of the soft palate | 3 | 547 |
| Pericardiac effusion, extraordinary quantity of | 20 | 178 |
| Pericarditis, M. Tacheron on | 2 | 326 |
| Pericarditis, ill treated case of | 3 | 242 |
| Pericarditis and gastritis, case of | 3 | 527 |
| Pericarditis, M. Louis on | 5 | 504 |
| Pericarditis, Dr. Louis's diagnosis of, | 5 | 523 |
| Pericarditis in a child, case of | 8 | 189 |
| Pericarditis, report on | 13 | 183 |
| Pericarditis from pneumo-thorax | 13 | 189 |
| Pericarditis, anatomical characters of, | 13 | 427 |
| Pericarditis, M. Louis on | 13 | 429 |
| Pericarditis, mercury in | 13 | 431 |
| Pericarditis from acute rheumatism | 14 | 166 |
| Pericarditis, Dr. Hope on | 16 | 366 |
| Pericarditis, case of | 18 | 457, 464 |
| Pericarditis, M. Louis on | 19 | 49 |
| Pericarditis, M Andral on | 19 | 49 |
| Pericarditis induced by a needle in the heart | 20 | 235 |
| Pericardium, tympanitic state of | 2 | 460 |
| Pericardium wanting, a case of | 6 | 235 |
| Pericardium, dropsy of | 6 | 623 |
| Pericardium, diseases of the | 9 | 385 |
| Pericardium, diseases of the | 13 | 426 |
| Pericardium, universal adhesions of | 18 | 501 |
| Pericranium, peculiar disease of the, | 8 | 485 |
| Pericranium, peculiar affection of the | 12 | 256 |
| Perineal fistula, new mode of treating | 11 | 265 |
| Perineal fistula, report on | 14 | 501 |
| Perineal fistula, remarks on | 14 | 505 |
| Perineum, severe rupture of | 3 | 288 |
| Perineum, contusion of | 10 | 534 |
| Perineum, extensive laceration of the, | 10 | 538 |
| Perineum, lacerated, operation for | 20 | 510 |
| Periodical bleedings, after amputation | 3 | 267 |
| Periodical medical press, retrospective review of the | 6 | 300 |
| Periodical peritonitis, some cases of, | 6 | 459 |
| Periodical head-ache | 10 | 52 |
| Periodical cerebral affection | 10 | 195 |
| Periodical vino-mania | 10 | 253 |
| Periosteum, diseases of the | 10 | 407 |
| Periostitis, Dr. Weir's report on | 14 | 481 |
| Periostitis, remarks on | 15 | 567 |
| Periostitis, Dr. Graves on | 18 | 481 |
| Peripneumonia of children | 9 | 67 |
| Peripneumonia, treatment of the | 9 | 70 |
| Peripneumony, percussion in | 2 | 69 |
| Peripneumony not *exactly* similar to hydrocephalus | 4 | 443 |
| Peripneumony, eclectic review on | 8 | 289 |
| Peripneumony in children, M. Guersent on | 8 | 493 |
| Periscope | 3 | 198 |
| Periscope, preface to it | 3 | 523 |
| Periscope, preface to the | 5 | 486 |
| Peritoneum, pathological anatomy of | 1 | 199 |
| Peritoneum, tubercles of | 1 | 302 |
| Peritonitis acuta | 1 | 200 |
| Peritonitis chronica | 1 | 202 |
| Peritonitis, Laennec's report on | 2 | 242 |
| Peritonitis, M. Tacheron on | 2 | 332 |
| Peritonitis, M. Andral on | 8 | 66 |
| Peritonitis, acute, cases of | 8 | 68 |
| Peritonitis, varieties of | 11 | 14 |
| Peritonitis, erysipelatous | 11 | 19 |
| Peritonitis, chronic | 11 | 21 |
| Peritonitis, chronic, Dr. Armstrong on | 11 | 106 |
| Peritonitis, obscure, cases of | 12 | 478 |

| | Vol. | Page |
|---|---|---|
| Peritonitis, fatal, from cold drink | 13 | 223 |
| Peritonitis, simulated by irritation | 13 | 331 |
| Peritonitis from ruptured liver | 15 | 269 |
| Peritonitis from lithotomy | 15 | 371 |
| Peritonitis, Dr. Lee on | 15 | 427 |
| Perry, Mr. on burns and scalds | 16 | 257 |
| Perspiration, acid in acute rheumatism | 9 | 176 |
| Perspiration, Dr. Edwards on | 18 | 21 |
| Persulphate of quinine, formulary of, | 2 | 483 |
| Pessary, Dr. Duffin's new | 15 | 281 |
| Petechiæ hæmorrhagicæ, case of | 4 | 139 |
| Petechial fever in Italy | 19 | 508 |
| Petition of Licentiates of London | 19 | 566 |
| Pett, Dr. his melancholy case | 5 | 368 |
| Phagedæna gangrenosa, case of | 6 | 259 |
| Phagedæna gangrenosa | 7 | 529 |
| Phagedæna, Mr. Fereday on | 7 | 551 |
| Phagedænic venereal disease, description of it | 3 | 463 |
| Phagedænic ulcer, its constitutional symptoms | 3 | 466 |
| Phagedænic affections of the joints | 3 | 468 |
| Phagedænic ulcer, Mr. Carmichael's treatment of | 3 | 470 |
| Phagedænic ulcer, Mr. Babington on, | 5 | 591 |
| Phagedænic ulceration | 7 | 245 |
| Phagedænic primary syphilis | 19 | 397 |
| Pharmacopœia, new | 1 | 56 |
| Pharmacopœia, Mr. Phillips on | 2 | 256 |
| Pharmacopœia, introduction to the | 16 | 513 |
| Pharmacy, manual of,—by Mr. Brande | 3 | 141 |
| Pharyngotomy, case of | 19 | 251 |
| Pharynx, food in the, for a year and a half | 11 | 166 |
| Pharynx, false passage through the | 15 | 391 |
| Phelan, Mr. on Irish pharmacy | 19 | 166 |
| Philadelphia, cholera gazette of | 20 | 178 |
| Philalethes on cold lotions | 13 | 153 |
| Philip, Dr. on galvanic experiments, | 3 | 589 |
| Philip, Dr. on protracted indigestion, | 7 | 362 |
| Philip, Dr. on indigestion | 7 | 362 |
| Philip, Dr. W. on functional disorders | 13 | 311 |
| Phillips, Mr. his pharmacopœia | 2 | 356 |
| Phillips, Mr. on diseases of urethra | 19 | 107 |
| Phillips, Mr. reclamation of | 19 | 573 |
| Phillips, Dr. on inflammation | 20 | 324 |
| Philosophical physiology, M. Bland's, | 15 | 510 |
| Phlebectasia, Sir A. Cooper's opinions | 4 | 402 |
| Phlebectasia; fatal case | 4 | 507 |
| Phlebitis, Messrs. Bouillaud and Ribes on | 4 | 504 |
| Phlebitis, some fatal cases of | 4 | 513 |
| Phlebitis, Mr. Guthrie on | 5 | 563 |
| Phlebitis encephalica, case of | 6 | 330 |
| Phlebitis, fatal cases of | 7 | 241, 245 |
| Phlebitis, Mr. Fereday on | 7 | 537 |
| Phlebitis, Dr. Forbes on | 8 | 230 |
| Phlebitis, cases of | 9 | 473 |
| Phlebitis, curious instance of | 10 | 468 |
| Phlebitis, Mr. Chinnock's case of | 11 | 155 |

| | Vol. | Page |
|---|---|---|
| Phlebitis, Mr. Arnott on | 12 | 1 |
| Phlebitis, fatal cases of | 12 | 5 |
| Phlebitis, secondary affection in | 12 | 19 |
| Phlebitis, uterine, Dr. Lee on | 12 | 376 |
| Phlebitis, idiopathic, case of | 15 | 240 |
| Phlebitis, cases of | 17 | 541 |
| Phlebitis, operation for | 17 | 543 |
| Phlebitis, Dr. R. Lee on | 19 | 60 |
| Phlebitis treated with tartar-emetic | 19 | 486 |
| Phlebitis, case of idiopathic | 20 | 253 |
| Phlegmasiæ, antimony in | 1 | 221 |
| Phlegmasiæ, obscure forms of | 17 | 442 |
| Phlegmatia dolens, essays on | 1 | 378 |
| Phlegmatia dolens, observations on, by Drs. Francis and Hosack | 1 | 378 |
| Phlegmatia dolens, Dr. Hosack on | 2 | 57 |
| Phlegmatia dolens, M. Velpeau on | 3 | 233 |
| Phlegmatia dolens, remarks on | 3 | 237 |
| Phlegmatia dolens, Mr. Davies's case, | 3 | 540 |
| Phlegmatia dolens, Dr. Davis's case of | 3 | 541 |
| Phlegmatia dolens, moxa in | 6 | 250 |
| Phlegmatia dolens, Mr. Fraser's case, | 6 | 464 |
| Phlegmatia dolens in a male | 10 | 526 |
| Phlegmatia dolens, Dr. Lee on | 11 | 380 |
| Phlegmatia dolens, cases of | 12 | 377 |
| Phlegmatia dolens, critical remarks | 12 | 381 |
| Phlegmatia dolens—inflamed veins | 14 | 212 |
| Phlegmatia dolens, Dr. R. Lee on | 19 | 61 |
| Phlegmonous erysipelas, tight bandage in | 5 | 554 |
| Phlegmonous erysipelas, Mr. Daniel on | 8 | 444 |
| Phlegmonous tumors in the right iliac fossa | 10 | 217 |
| Phlegmonous tumors in right iliac fossa | 16 | 225 |
| Phlegmonous erysipelas, remarks on, | 20 | 323 |
| Phosphate of soda in diabetes | 2 | 237 |
| Phosphate of iron, in diabetes | 13 | 74 |
| Phosphorescence of the retina | 17 | 470 |
| Phosphorus used extensively in palsy, | 20 | 204 |
| Phrenic neuralgiæ, cases of | 9 | 334 |
| Phrenitis, remarkable case of, by Mr. Rhind | 1 | 299 |
| Phrenitis, otorrhœa purulenta mistaken for | 11 | 542 |
| Phrenological character of Burke | 11 | 193 |
| Phrenological estimate of the negroes, | 19 | 151 |
| Phrenological development of Dr. Spurzheim | 19 | 152 |
| Phrenological character of the Esquimaux | 19 | 474 |
| Phrenology, Mr. Coombe on | 2 | 251 |
| Phrenology, Mr. Coombe's system of | 5 | 437 |
| Phrenology and physiognomy, Dr. Spurzheim on | 5 | 468 |
| Phrenology, remarks on | 12 | 291 |
| Phrenology, Mr. Combe on | 14 | 321 |
| Phrenology, Dr. Elliotson's belief in, | 15 | 173 |
| Phrenology of satyriasis | 19 | 238 |
| Phthisical persons, West Indies for | 13 | 81 |
| Phthisis, on the tonic treatment of | 1 | 256 |
| Phthisis, hepatic | 2 | 184 |

| | Vol. | Page |
|---|---|---|
| Phthisis pulmonalis and bronchitis.. | 3 | 496 |
| Phthisis, state of the stomach in.... | 3 | 530 |
| Phthisis pulmonalis cured ........ | 4 | 195 |
| Phthisis, the exhalations of marine plants in .................... | 4 | 199 |
| Phthisis, state of the expectoration in | 4 | 469 |
| Phthisis cured by mercury ........ | 6 | 271 |
| Phthisis pulmonalis, objections to the term .................... | 6 | 331 |
| Phthisis pulmonalis in India ....... | 10 | 401 |
| Phthisis, recovery from.. ........ | 11 | 85 |
| Phthisis, Dr. Clarke on climate in .. | 12 | 90 |
| Phthisis, causes of................ | 12 | 100 |
| Phthisis, symptomatology of....... | 12 | 102 |
| Phthisis, percussion and auscultation in ...................... | 12 | 104 |
| Phthisis, complications of.......... | 12 | 106 |
| Phthisis, course of................ | 12 | 107 |
| Phthisis, treatment of ............ | 12 | 108 |
| Phthisis, climate in .............. | 12 | 109 |
| Phthisis, is it contagious? ......... | 12 | 111 |
| Phthisis pulmonalis, Dr. Parrish on.. | 12 | 241 |
| Phthisis, extraordinary case of...... | 13 | 529 |
| Phthisis laryngea, case of.......... | 13 | 543 |
| Phthisis, inhalation of chlorine in... | 14 | 103 |
| Phthisis, inhalation of chlorine in... | 15 | 208 |
| Phthisis cured by turtle........... | 15 | 284 |
| Phthisis of spinners, Dr. Kay on.... | 15 | 477 |
| Phthisis, Dr. Blackmore's notions on, | 17 | 402 |
| Phthisis, state of fingers in ........ | 18 | 189 |
| Phthisis, pulmonary, use of whey in, | 20 | 197 |
| Phthisis, Dr. Stokes on ........... | 20 | 257 |
| Phymosis, cases of................ | 15 | 233 |
| Physic, Drs. Uwins and Dawson on.. | 2 | 382 |
| Physic, elements of the practice of, by Dr. Gregory.............. | 5 | 408 |
| Physic and surgery—fever ........ | 8 | 571 |
| Physic, Dr. Gregory's elements of .. | 10 | 115 |
| Physic and surgery, their distinctions | 18 | 225 |
| Physic and surgery, intimate union of | 20 | 282 |
| Physical signs of diseases of the lungs, Dr. Williams on ....... | 10 | 450 |
| Physical agents, Dr. Edwards on ... | 18 | 1 |
| Physician, answer to a letter from .. | 5 | 635 |
| Physician, a portion of his duty .... | 15 | 199 |
| Physician, the qualifications of a.... | 19 | 227 |
| Physicians, opening of their new college ...................... | 3 | 586 |
| Physicians, dissensions among...... | 5 | 636 |
| Physicians, charter of the College of, | 7 | 505 |
| Physicians, soirées at the College of, | 12 | 520 |
| Physicians, lives of British ........ | 13 | 488 |
| Physicians, London, memorial of .. | 19 | 192 |
| Physicians in Turkey ............ | 19 | 433 |
| Physics, Dr. Arnott's elements of .. | 7 | 35 |
| Physics, Dr. Arnott's elements of. .. | 12 | 334 |
| Physiological metamorphoses ...... | 1 | 436 |
| Physiological propositions ......... | 6 | 314 |
| Physiological studies............... | 13 | 498 |
| Physiological doctrine, Broussais'; sketch of ...................... | 18 | 469 |
| Physiology, therapeutical.......... | 1 | 176 |
| Physiology of the spleen, &c. ....... | 15 | 47 |
| Physiology, Alison's outlines of..... | 15 | 75 |
| Physiology, outlines of, by Mr. Mayo | 19 | 91 |
| Physiology of the fœtal heart ...... | 20 | 163 |
| Pigeaux, M. on the cardiac sounds.. | 19 | 216 |
| Pigeaux on the sounds of the heart.. | 20 | 133 |
| Piles, new operation for .......... | 6 | 271 |
| Piles, Mr. Mayo on ............... | 19 | 296 |
| Pilgrim in pursuit of health........ | 19 | 183 |
| Pilimiction, cases of..... ........ | 8 | 535 |
| Pills, a rather odd mercurial one.... | 3 | 354 |
| Pinch of snuff, pathology of........ | 2 | 250 |
| Pin-prick, followed by extensive suppuration ................. | 10 | 552 |
| Piorry sur la physiologie .......... | 15 | 488 |
| Piorry, M. on syncope............. | 16 | 233 |
| Piorry on hemicrania.............. | 16 | 236 |
| Piorry, M. on mediate auscultation.. | 20 | 337 |
| Pitié, La, clinical report from ...... | 9 | 255 |
| Placenta, inflammation of.......... | 9 | 194 |
| Placenta, new mode of hastening its separation................ | 9 | 439 |
| Placenta, morbid conditions of the .. | 17 | 344 |
| Placenta, rupture of the. ........... | 17 | 349 |
| Placenta, adhesion of the.......... | 17 | 354 |
| Placenta, Dr. Lee on the .......... | 17 | 454 |
| Placenta, structure of, Mr. Owen on, | 19 | 65 |
| Placenta, structure of, Mr. Millard on | 19 | 66 |
| Placenta, adhesion of the........... | 19 | 498 |
| Placental presentation, a fatal case.. | 5 | 614 |
| Placentary bruit, Dr. Kennedy on .. | 20 | 101 |
| Plague, symptoms of, from animal effluvia ............... .... | 2 | 202 |
| Plague of Athens, its probable nature | 3 | 74 |
| Plague and yellow fever, differences between .................. | 3 | 82 |
| Plague, isolation in, its effect ...... | 5 | 235 |
| Plague, the Chevalier de Butel on .. | 5 | 253 |
| Plague, Mr. Madden's experience in, | 11 | 144 |
| Plague, Mr. Madden on the......... | 11 | 323 |
| Plague, etiology of the............. | 11 | 326 |
| Plague at Malta................... | 14 | 339 |
| Plantar nerve, curious disease of.... | 9 | 554 |
| Plants, the physiology of.......... | 19 | 232 |
| Plaster of Paris casts for fractures .. | 16 | 247 |
| Plaster-moulds for fractured limb .. | 20 | 492 |
| Plastic inflammations, lunar caustic in | 9 | 439 |
| Plethora and cause of convulsions .. | 5 | 156 |
| Plethora, observations on........... | 13 | 315 |
| Plethora from suppressed evacuation, | 14 | 487 |
| Pleurisy, Laennec's report on ...... | 2 | 241 |
| Pleurisy & abscesses after operations, | 6 | 521 |
| Pleurisy, &c. simulated by affections not inflammation ............ | 11 | 478 |
| Pleuritis, instructive case of ...... | 4 | 139 |
| Pleuritis hæmorrhagica—operation.. | 11 | 462 |
| Pleuritis and pneumo-thorax, case of | 15 | 212 |
| Pleuritis, Dr. Stokes on .......... | 20 | 257 |
| Pleuro-pneumonia, cases of........ | 19 | 225 |
| Pleximetre, M. Piorry's .......... | 6 | 496 |
| Pleximeter, M. Piorry's .......... | 10 | 421 |
| Plica polonica on the ............. | 19 | 214 |

| | Vol. | Page |
|---|---|---|
| Plica polonica, M. Boismont on .... | 20 | 215 |
| Ploucquet, the Clutterbuck of yore, | 5 | 15 |
| Plymouth, yellow fever at ........ | 9 | 515 |
| Pneumo-gastric nerves, section of the | 8 | 215 |
| Pneumo-gastric neuralgiæ, cases of, | 9 | 335 |
| Pneumo-phthisis cyanotica, M. Schonlein on ................ | 8 | 193 |
| Pneumo-thorax, curious cases of.... | 9 | 566 |
| Pneumo-thorax, case of .......... | 10 | 456 |
| Pneumo-thorax in a medical gentleman .................... | 10 | 478 |
| Pneumo-thorax, observations on ... | 10 | 482 |
| Pneumo-thorax, interesting case of.. | 12 | 466 |
| Pneumo-thorax, case of. .......... | 13 | 453 |
| Pneumo-thorax, history of ........ | 13 | 455 |
| Pneumo-thorax, Mr. Spittal on .... | 14 | 507 |
| Pneumonia, large doses of antimony in | 1 | 221 |
| Pneumonia, on the treatment of.... | 2 | 236 |
| Pneumonia ..................... | 3 | 508 |
| Pneumonia, its site .............. | 3 | 512 |
| Pneumonia, auscultation in........ | 3 | 513 |
| Pneumonia ending in gangrene, case, | 3 | 521 |
| Pneumonia without pain .......... | 7 | 458 |
| Pneumonia mistaken for hydrothorax | 7 | 482 |
| Pneumonia, Laennec's treatment of.. | 8 | 107 |
| Pneumonia, M. Broussais treatment of | 8 | 144 |
| Pneumonia carditis, case of........ | 9 | 251 |
| Pneumonia, treated by antimony.... | 13 | 567 |
| Pneumonia biliosa, Mr. Allen on.... | 14 | 262 |
| Pneumonia, tartar-emetic in ...... | 18 | 175, 479 |
| Pneumonia hypostatica, on........ | 19 | 212 |
| Poets, are they mad?............. | 13 | 300 |
| Poison preserves a human stomach.. | 2 | 250 |
| Poison of hydrophobia............ | 13 | 435 |
| Poisoned wounds, the cupping-glass in | 5 | 322 |
| Poisoned wounds, Mr. Travers on .. | 5 | 363 |
| Poisoned wounds, effects of compression in.................. | 6 | 232 |
| Poisoned confectionary............ | 15 | 201 |
| Poisoning by opium .............. | 2 | 235 |
| Poisoning, case of, by euphorbia peplus ..................... | 3 | 275 |
| Poisoning by arsenic, Orfila's case . | 3 | 289 |
| Poisoning by arsenic, curious instance of.................. | 3 | 291 |
| Poisoning, by arsenic and sublimate, | 3 | 565 |
| Poisoning by laudanum ; instance of, | 4 | 270 |
| Poisoning by cantharides, a case of.. | 4 | 272 |
| Poisoning by belladonna .......... | 7 | 233 |
| Poisoning by arsenic, Dr. Christison, | 7 | 379 |
| Poisoning by sulphuric acid........ | 9 | 222 |
| Poisoning from morphine, prescribed by mistake ............... | 11 | 551 |
| Poisoning from laudanum, mysterious case.................. | 11 | 551 |
| Poisoning, general theory of........ | 12 | 365 |
| Poisoning, symptoms of........... | 12 | 369 |
| Poisoning, imaginary or pretended .. | 12 | 374 |
| Poisoning, supposed, by mercury ... | 13 | 472 |
| Poisoning by opium and belladonna, | 13 | 563 |
| Poisoning, case of, by Foderé .... . | 18 | 183 |

| | Vol. | Page |
|---|---|---|
| Poisoning by sulphuric acid ....... | 18 | 530 |
| Poisoning from the fumes of arsenic, | 19 | 504 |
| Poisoning from hyosciamus seeds.... | 20 | 205 |
| Poisonous matter in offal.......... | 7 | 166 |
| Poisons, Dr. Paris on ............ | 1 | 316 |
| Poisons, Dr. Addison and Mr. Morgan on ..................... | 11 | 32 |
| Poisons, mode of action of ........ | 11 | 35 |
| Poisons, experiments on .......... | 11 | 42 |
| Poisons, Dr. Christison's treatise on, | 12 | 362 |
| Poisons, Dr. Roupell on .......... | 20 | 62 |
| Polish carbuncle, history of the. .... | 11 | 204 |
| Politics, medical ................. | 20 | 561 |
| Polyphagia, M. Beaudè's case of.... | 5 | 596 |
| POLYPHARMACY, beauties of ...... | 9 | 64 |
| Polypi, nasal, case of ............. | 7 | 265 |
| Polypi, vesicular and fleshy ........ | 11 | 403 |
| Polypi of the heart as a disease...... | 15 | 96 |
| Polypi, cardiac, purulent matter in.. | 19 | 214 |
| Polypi of the heart .............. | 19 | 518 |
| Polypi, treatment of by Graefe ..... | 20 | 219 |
| Polypus uteri, Mr. Stone on ....... | 6 | 210 |
| Polypus, malignant, bleeding, case of, | 8 | 478 |
| Polypus uteri, case of ............. | 10 | 227 |
| Polypus of the uterus, Dr. Macfarlane on .............. ...... | 10 | 373 |
| Polypus of the uterus, with pregnancy | 10 | 375 |
| Polypus, malignant, of the uterus .. | 10 | 380 |
| Polypus uteri and pregnancy....... | 11 | 571 |
| Polypus of the uterus, Dr. Gooch on, | 12 | 321 |
| Polypus uteri, remarkable case of. .. | 12 | 324 |
| Polypus uteri, diagnosis of ........ | 12 | 325 |
| Polypus uteri, ligature for. ........ | 12 | 326 |
| Polypus uteri, cases of, &c. ........ | 17 | 317 |
| Pomegranate root, for the expulsion of tape-worm ........... | 3 | 574 |
| Pompeii, "Weiss's dilator" found in, | 12 | 247 |
| Pompeii, reflections on............ | 14 | 460 |
| Pomponius Atticus, cases of........ | 15 | 572 |
| Pontine fens, passage of........... | 14 | 459 |
| Pope, Mr. on sarsaparilla, &c....... | 1 | 47 |
| Pope's character, by Mr. Madden ... | 19 | 319 |
| Popliteal aneurism, Mr. Allan...... | 5 | 257 |
| Popliteal aneurism in both legs, operation .................. | 8 | 476 |
| Popliteal aneurism—Mr. Brodie's case | 8 | 541 |
| Popliteal aneurism, secondary hæmorrhage ................. | 8 | 564 |
| Popliteal aneurism, case of, and remarks on ................. | 10 | 228 |
| Popliteal aneurism—operation—gangrene ................. | 11 | 263 |
| Popliteal aneurism, cases of........ | 13 | 283 |
| Popliteal aneurism ............... | 13 | 365 |
| Population of Naples.............. | 15 | 520 |
| Population, statistical researches on, | 17 | 182 |
| Porrigo, ointments for the cure of .. | 1 | 224 |
| Porrigo and psoriasis, cases of...... | 11 | 455 |
| Porrigo, M. Biett on.............. | 16 | 398 |
| Porrigo, Dr. Roots on ............ | 19 | 539 |
| Porrigo, Mr. Macilwain on ........ | 20 | 348 |
| Porrigo, characters and origin of.... | 20 | 350 |

| | Vol. | Page |
|---|---|---|
| Porrigo, contagiousness of ........ | 20 | 351 |
| Porrigo, dietetic management of.... | 20 | 354 |
| Porrigo, medicinal treatment of .... | 20 | 358 |
| Porrigo decalvans treated by tartar emetic .................... | 20 | 453 |
| Port Glasgow, cholera at. ........ | 16 | 130 |
| Portal, M. death of ............... | 18 | 475 |
| Portal, legacy left by ............. | 19 | 506 |
| Portending symptoms of rupture of the uterus................ | 4 | 28 |
| Porter, Mr. on the surgical pathology of the larynx, &c......... | 6 | 375 |
| Porter, dangerous symptoms from drinking.................. | 10 | 530 |
| Porter, Mr. on aneurism of the aorta, | 20 | 261 |
| Porter, Mr. on amputation in spreading gangrene .......... | 20 | 265 |
| Portio dura and fifth pair, their functions considered.......... | 6 | 67 |
| Portio dura, neuralgia of.......... | 19 | 425 |
| Portrait of Joshua Brookes ........ | 20 | 189 |
| Position of the limb during sawing of the bone ................. | 3 | 50 |
| Posterior tibial artery, puncture of.. | 6 | 247 |
| Posterior tibial artery, aneurism of the | 10 | 469 |
| Posterior tibial wounded .......... | 13 | 138 |
| Post-mortem appearances, in cholera | 3 | 131 |
| Post-mortem changes, experiments on | 6 | 295 |
| Post-mortem examination of Talma, | 6 | 465 |
| Post-mortem changes in mucous membranes .................. | 6 | 474 |
| Post-mortem examinations of the kings of France.............. | 12 | 259 |
| Post-mortem appearances in insanity | 18 | 502 |
| Pouillet, M. on atmospheric electricity | 4 | 485 |
| Poultry, epidemic disease among.... | 17 | 439 |
| Powell, Mr. on twin-conception.... | 1 | 130 |
| Practical men, their errors......... | 15 | 36 |
| Practice, mode of getting into...... | 13 | 479 |
| Pratt, Mr. his prediction .......... | 3 | 38 |
| Precautions in opening bodies...... | 3 | 255 |
| Preceptor, the .................... | 20 | 151 |
| Predisposition, a want of power in arteries ...................... | 3 | 197 |
| Predisposition to convulsions ...... | 5 | 151 |
| Pregnancy, fatal constipation in .... | 1 | 233 |
| Pregnancy with ascites............ | 1 | 492 |
| Pregnancy, nervous............... | 1 | 495 |
| Pregnancy and ascites, cases of .... | 2 | 505 |
| Pregnancy, application of the stethoscope in .................... | 5 | 607 |
| Pregnancy complicated with hydatids | 6 | 270 |
| Pregnancy, fatal sickness from...... | 8 | 149 |
| Pregnancy, Dr. Gooch on.......... | 12 | 42 |
| Pregnancy, fallacious symptoms of.. | 12 | 46 |
| Pregnancy, auscultation evidence of, | 14 | 465 |
| Pregnancy, auscultation in ........ | 14 | 495 |
| Pregnancy, extra-uterine, probable cause of .................. | 17 | 173 |
| Pregnancy, extra-uterine, Dr. Ramsbotham on..................... | 17 | 310 |
| Pregnancy, extra-uterine.......... | 19 | 506 |
| Pregnancy, doubtful, Dr. Ramsbotham on.................. | 17 | 314 |
| Pregnancy, visible signs of ........ | 20 | 96 |
| Pregnancy, tangible signs of ...... | 20 | 98 |
| Pregnancy, audible signs of........ | 20 | 100 |
| Pregnancy, simulated ............. | 20 | 119 |
| Pregnancy, compound ............. | 20 | 112 |
| Pregnancy, complicated .......... | 20 | 113 |
| Pregnancy, peritoneal, extra-uterine, | 20 | 239 |
| Premature labour, observations on.. | 1 | 355 |
| Premature labour, operation for inducing ....................... | 4 | 158 |
| Prepuce, removal of, followed by pneumonia .................. | 10 | 577 |
| Prepuce, contraction of ........... | 19 | 282 |
| Preputii herpes.................. | 3 | 556 |
| Preservation of dead subjects ...... | 2 | 248 |
| Preservation of the teeth.......... | 6 | 136 |
| Press, radical, liberty of the........ | 12 | 438 |
| Press, periodical ................. | 19 | 284 |
| Pressure on the perineum in labour.. | 2 | 530 |
| Pressure, its effects on bone........ | 4 | 58 |
| Pressure for ascites ............... | 10 | 224 |
| Preternatural presentation, case of.. | 2 | 507 |
| Prevention of gout .............. | 4 | 49 |
| Priapism, curious case of.......... | 1 | 214 |
| Prichard, Dr. on the vital principle.. | 12 | 316 |
| Primary and secondary amputation, cases of ...................... | 11 | 279 |
| Principles of dental surgery, by Mr. Koecker ................ | 6 | 129 |
| Principles of medicine, by M. Broussais | 6 | 313 |
| Prison-discipline ................ | 1 | 245 |
| Prize hospital report, Mr. Smith's .. | 6 | 601 |
| Prize hospital report, Mr. Bury .... | 7 | 249 |
| Prize hospital reports, Mr. Fereday.. | 7 | 537 |
| Prize hospital report, Mr. Turner ... | 7 | 569 |
| Prize hospital report, Mr. Wickham's | 8 | 249 |
| Prize essay on temperament........ | 13 | 1 |
| Prizes of the Institute of France.... | 18 | 542 |
| Proceedings of the Obstetric Society, | 13 | 241 |
| Procidentia uteri, case of....... .. | 12 | 162 |
| Profession, tax on those entering.... | 13 | 440 |
| Professional or unprofessional? .... | 10 | 499 |
| Professional omnibuses............ | 14 | 145 |
| Professional morality in 1831 ...... | 14 | 467 |
| Professor Delpech's case of wounded carotid .................. | 3 | 244 |
| Professor Bennett's last illness. .... | 15 | 215 |
| Progress of physiological science.... | 20 | 493 |
| Prolapsed uterus extirpated........ | 4 | 580 |
| Prolapsus recti .................. | 2 | 284 |
| Prolapsus ani.................... | 12 | 154 |
| Prolapsus of the uvula............ | 13 | 540 |
| Prolapsus uteri, Baron Larrey on. .. | 14 | 259 |
| Prolapsus ani in grown persons..... | 15 | 483 |
| Prolapsus ani, cases of............ | 15 | 484 |
| Prolapsus ani, operation for........ | 15 | 485 |
| Prolapsus ani, with stricture of rectum | 15 | 486 |
| Prolapsus of rectum, Mr. Salmon on, | 16 | 369 |
| Prolapsus of rectum, treatment of .. | 16 | 371 |
| Prolapsus of rectum, M. Dupuytren on | 18 | 365 |

| | Vol. | Page |
|---|---|---|
| Prolapsus ani, Dr. Macfarlane on .. | 18 | 447 |
| Proofs of Sir A. Cooper's early opinions of non-union, in fracture of the neck of the femur .. | 3 | 53 |
| Propagation of plants | 17 | 394 |
| Properties and history of the euphorbia lathyrus | 3 | 274 |
| Prospectus of the Crown Company.. | 2 | 557 |
| Prospectus of a new medical journal, | 9 | 286 |
| Prostate gland, section of, in lithotomy | 1 | 210 |
| Prostate gland & vesiculæ, diseases of the | 11 | 459 |
| Prostate, incision of, in lithotomy .. | 12 | 73 |
| Prostate gland, abscess of | 18 | 445 |
| Prostration of the system after injuries .. | 5 | 389 |
| *Prostration*, treatment of this state.. | 6 | 108 |
| *Prostration*, individual symptoms of, | 6 | 111 |
| Proteian malady, M. Sarrut's own case | 7 | 149 |
| Proteian malady | 7 | 412 |
| Protracted lactation, Dr. Morton on, | 7 | 488 |
| Provincial Medical Gazette, remarks on the | 11 | 152 |
| Provincial Medical and Surgical Association | 17 | 574 |
| Provincial Association, transactions of | 19 | 358 |
| Provincial schools of medicine | 20 | 180 |
| Prurigo, cured by colchicum | 7 | 529 |
| Pruritus of the genitals, Dr. Davis on | 16 | 579 |
| Prussian armies, revaccination of ... | 20 | 195 |
| Prussian divisions of medical men .. | 20 | 563 |
| Prussic acid, poisoning by | 1 | 506 |
| Prussic acid in catarrh and phthisis, | 6 | 532 |
| Prussic acid, case of poisoning by .. | 9 | 461 |
| Pseudo-pregnancy | 20 | 114 |
| Pseudo-syphilitic affections of the throat | 20 | 211 |
| Psoas abscess, injection of its cavity, | 10 | 153 |
| Psoas muscle, curious tumor attached to | 15 | 157 |
| Psoas abscess, connected with hydrocephalus | 18 | 160 |
| Psoriasis, successful treatment of ... | 7 | 479 |
| Psoriasis diffusa, case of | 12 | 276 |
| Psoriasis treated by depletion | 14 | 285 |
| Psoriasis, M. Biett on | 16 | 405 |
| Psoris papulosa, M. Alibert on | 7 | 189 |
| Psoris crustacea, M. Alibert on | 7 | 191 |
| Ptosis, new operation for | 14 | 243 |
| Ptyalism, an antidote for | 1 | 231 |
| Ptyalism, Mr. Arnott on | 2 | 153 |
| Ptyalism, chlorate of soda in | 4 | 574 |
| Ptyalism, Dr. Graves on | 17 | 458 |
| Pubes, separation of, cases of | 2 | 526 |
| Puerperal fever, Mr. Hey on | 1 | 414 |
| Puerperal fever, observations on | 2 | 243 |
| Puerperal peritonitis, Mr. Davis on.. | 2 | 503 |
| Puerperal irritability, Mr. Waller on, | 2 | 503 |
| Puerperal fever of Vienna, report of, | 2 | 509 |
| Puerperal neuritis | 2 | 523 |
| Puerperal neuritis, its varieties | 2 | 524 |
| Puerperal peritonitis | 7 | 201 |
| Puerperal convulsions, ergot of rye in | 9 | 484 |
| Puerperal peritonitis, Mr. Ashwell on | 10 | 113 |
| Puerperal peritonitis, rapidly fatal .. | 10 | 201 |
| Puerperal diseases, Dr. Hall on | 11 | 289 |
| Puerperal inflammation, Dr. Hall's treatment of | 11 | 295 |
| Puerperal peritonitis, Dr. Gooch on.. | 11 | 299 |
| Puerperal disease *not* peritonitis | 11 | 303 |
| Puerperal affections, treatment of. .. | 11 | 312 |
| Puerperal mania, Dr. Marshall Hall on | 11 | 359 |
| Puerperal mania, danger of | 11 | 361 |
| Puerperal mania, etiology of | 11 | 362 |
| Puerperal mania, proximate cause of, | 11 | 364 |
| Puerperal mania, treatment of | 11 | 368 |
| Puerperal fever, pathology of | 13 | 286 |
| Puerperal fever, suppuration of the veins in | 13 | 288 |
| Puerperal convulsions | 15 | 22 |
| Puerperal fever, insidious form | 15 | 518 |
| Puerperal fever, Dr. Ryan on | 17 | 115 |
| Puerperal fever, symptoms of | 17 | 116 |
| Puerperal fever, treatment of | 17 | 118 |
| Puerperal convulsions, Dr. Ramsbotham on | 17 | 304 |
| Puerperal phlebitis, on | 19 | 199 |
| Puerperal fever, pathology of | 19 | 200 |
| Puerperal pathology | 19 | 330 |
| Puff, professional | 12 | 146 |
| Puffy tumor of scalp from effusion.. | 13 | 558 |
| Pulmonary exhalation, experiments on | 5 | 612 |
| Pulmonary apoplexy | 7 | 466 |
| Pulmonary abscess, Dr. Chambers on | 8 | 138 |
| Pulmonary abscess, true, case of | 9 | 453 |
| Pulmonary diseases, breathing cool air in | 9 | 484 |
| Pulmonary tubercles, signs of | 10 | 501 |
| Pulmonary apoplexy, case of | 10 | 514 |
| Pulmonary affections, stethoscope in, | 11 | 497 |
| Pulmonary apoplexy, cases of | 12 | 87 |
| Pulmonary phthisis, eclectic article.. | 12 | 97 |
| Pulmonary apoplexy, curious cases of | 12 | 555 |
| Pulmonary affections at Malta | 14 | 342 |
| Pulmonary apoplexy, two cases of .. | 14 | 360 |
| Pulmonary abscess over the clavicle, | 15 | 566 |
| Pulmonary apoplexy, Dr. Townsend on | 16 | 470 |
| Pulmonary and cutaneous functions in frogs | 18 | 6 |
| Pulmonary abscess, extraordinary case | 20 | 192 |
| Pulmonary disease, acetas plumbi in, | 20 | 193 |
| Pulmonic diseases, Dr. Cobb on | 3 | 217 |
| Pulsating tumors of the scalp, cases of | 8 | 497 |
| Pulsating tumors of the scalp, cases of, with remarks | 10 | 507 |
| Pulsation in the veins, Dr. Davis's case of | 7 | 118 |
| Pulse, observations on the | 2 | 422 |
| Pulse, remarkable state of, in a case of epilepsy | 4 | 502 |
| Pulse, intermissions of the, M. Laennec on | 9 | 22 |

| | Vol. | Page |
|---|---|---|
| Pulse *not* synchronous with the impulse of the heart | 13 | 128 |
| Pulse, effects of posture on the | 15 | 152 |
| Pulse, Dr. Burne on the | 15 | 474 |
| Pulse, cessation of, in both arms | 19 | 285 |
| Pulse, state of, in hydrocephalus | 19 | 445 |
| Pulse, fœtal, frequency of the | 20 | 105 |
| Pulvis antimonialis, experiments on, | 1 | 227 |
| Puncture of the rectum | 9 | 497 |
| Punctured wounds treated by nitrate of silver | 10 | 447 |
| Pupil, state of the, in deep intoxication | 8 | 449 |
| Purgation, its frequent bad consequences | 5 | 31 |
| Purgation, its uses and abuses | 6 | 504 |
| Purgation, its powers in disease | 6 | 604 |
| Purgatives, objections to, in chorea, | 4 | 10 |
| Purgatives in irritability of the brain, | 4 | 438 |
| Purification of Thames' water | 12 | 443 |
| Purification of dissecting theatres | 20 | 516 |
| Purpura, Dr. Stoker on | 1 | 144 |
| Purpura hæmorrhagica | 1 | 482 |
| Purpura hæmorrhagica | 2 | 193 |
| Purpura hæmorrhagica, Dr. Belcher's case of | 3 | 284 |
| Purpura hæmorrhagica, Dr. Fairbairn's case of | 6 | 61 |
| Purpura hæmorrhagica, Drs. Hawkins and Chambers on | 6 | 200 |
| Purpura hæmorrhagica, cases of | 7 | 468 |
| Purpura hæmorrhagica, Mr. Kingley's case | 8 | 207 |
| Purpura hæmorrhagica, venesection for | 9 | 213 |
| Purpura hæmorrhagica, case of | 11 | 237 |
| Purpura, M. Biett on | 17 | 379 |
| Purpura, pathology of | 19 | 153 |
| Purpura hæmorrhagica, fatal case of, | 19 | 210 |
| Purpura hæmorrhagica | 19 | 444 |
| Purulent ophthalmia of infants | 2 | 220 |
| Purulent depôts, after wounds, &c | 8 | 470 |
| Purulent ophthalmia following gonorrhœa | 10 | 528 |
| Purulent depôts, Mr. Arnott's theory | 12 | 21 |
| Purulent depôts, curious case of | 12 | 382 |
| Purulent depôts in puerperal fever | 13 | 287 |
| Purulent discharge from the ear; cases | 15 | 303 |
| Purulent deposites and phlebitis | 18 | 398 |
| Purulent deposites, theories of | 18 | 402 |
| Purulent ophthalmia, Mr. Middlemore on | 18 | 511 |
| Purulent cachexy, on the | 19 | 213 |
| Pus in the cavities of the heart | 11 | 475 |
| Pus discharged from ear, after injury of the head | 17 | 538 |
| Pustular venereal disease, character of the | 3 | 461 |
| Pustules of variola punctured by a needle | 11 | 148 |
| Putrefaction of the lungs | 1 | 345 |
| Puzzling point for the bench of bishops | 6 | 268 |
| Pylorus, bony tumor of the | 8 | 508 |
| Pylorus, scirrhus of the | 1 | 191, 462 |
| Pylorus, stricture about the | 10 | 120 |
| Pylorus, scirrhus of the | 12 | 164 |
| Pylorus, disease of, cured by narcotics | 17 | 168 |
| Pym, Sir W. his letter on cholera | 16 | 177 |
| Pyramus frigate, fever in | 2 | 11 |
| Pyretologists, various sects of the | 9 | 4 |
| Pyrmont, vapour cave at | 20 | 201 |
| Pyroligneous acid, its action on the ear | 3 | 116 |
| Pythagoras redivivus, in the person of Sir G. Gibbs | 8 | 213 |
| **Q** | | |
| Quack medicines—French regulations on | 10 | 254 |
| Quackery & the regulars, in France, | 3 | 344 |
| Qnackery, Democritus, jun. on | 14 | 193 |
| Quain, Mr. his translation of Martinet's pathology | 6 | 407 |
| Quain, Mr. his elements of anatomy, | 10 | 67 |
| Quain, Mr. his introductory lectures, | 14 | 260 |
| Quain, Mr. his elements of anatomy, | 18 | 242 |
| Quarantine, Dr. Maclean on | 2 | 18 |
| Quarantine, late regulations for | 3 | 292 |
| Quarantine establishments, inutility of | 20 | 234 |
| Quickening, what is it? | 13 | 109 |
| Quicksilver, metallic, effects of | 19 | 233 |
| Quinina and sulphate of quinina | 1 | 321 |
| Quinine, sulphate of | 1 | 51 |
| Quinine, sulphate of, in typhus | 2 | 237 |
| Quinine, sulphate of, its powers | 2 | 488 |
| Quinine, employed in friction | 6 | 243 |
| Quinine in intermittents | 6 | 606 |
| Quinine in acute rheumatism | 13 | 437 |
| Quinine, report on the use of | 17 | 409 |
| Quinine, affections of head produced by | 17 | 522 |
| Quinine, hydrocyanate of | 19 | 219 |
| Quinine in strumous ophthalmia | 19 | 258 |
| **R** | | |
| Rachitome, double | 13 | 570 |
| Radcliffe, Dr. memoir of | 7 | 39 |
| Radial arteries, unequal pulse in | 7 | 279 |
| Radius, dislocation of, forwards and upwards | 10 | 240 |
| Radius, head of, dislocated backwards | 12 | 178 |
| Radix caincæ, as a diuretic | 18 | 501 |
| Raikem, Dr. on inflammation of the spleen | 12 | 252 |
| Rainey, Mr. on arsenic | 17 | 198 |
| *Râles*, varieties of, with their causes, | 4 | 456 |
| Ramadge, Dr. and St. John Long | 15 | 178 |
| Rambling notes of Sir A.B. Faulkner, | 8 | 27 |
| Ramollissement, partial, of the brain, | 12 | 215 |
| Ramollissement of the spinal cord | 12 | 487 |
| Ramollissement of the brain | 15 | 322 |
| Ramollissement of spinal marrow | 17 | 23 |
| Ramsbotham, Dr. on midwifery | 17 | 289 |

| | Vol. | Page |
|---|---|---|
| Ranula, M. Breschet on | 9 | 225 |
| Ranula, treated by the seton | 10 | 578 |
| Ranula treated by the seton | 11 | 263 |
| Rare cases in surgery, by Scarpa | 6 | 221 |
| Rassori on antimony in large doses | 1 | 221 |
| Ratier, Dr. on the Parisian hospitals, | 3 | 95, 344 |
| Ratiocination of Dr. Stevens | 17 | 323 |
| Reaction afterdepletion, description of | 4 | 353 |
| Reaction, excessive, after prostration, | 6 | 113 |
| Reaction, Dr. Hall on | 18 | 355 |
| Reason, dawn of, in the child | 13 | 291 |
| Recamier, M. his hospital reports | 3 | 217 |
| Recamier, M. on cold affusion | 13 | 462 |
| Receipt for an opening aloetic pill | 3 | 147 |
| Recollection and memory; distinctions | 18 | 549 |
| Recto-vesical lithotomy | 2 | 473 |
| Recto-vesical operation, case of | 6 | 552 |
| Recto-vaginal abscess, mode of laying open | 11 | 175 |
| Recto-urethral fistula cured by the cautery | 11 | 282 |
| Recto-vesical lithotomy, Mr. Brodie's | 14 | 187 |
| Recto-vaginal fistula, cured | 17 | 486 |
| Rectum, Mr. Salmon on stricture of, | 8 | 325 |
| Rectum, neuralgia of the | 10 | 59 |
| Rectum, scirrho-contracted, curious case of | 10 | 208 |
| Rectum, operation for cancer of the | 10 | 581 |
| Rectum, imperforate, treatment of | 11 | 261 |
| Rectum, extirpation of the lower part | 13 | 148 |
| Rectum, Mr. Macilwain on | 13 | 193 |
| Rectum, treatment of stricture of | 13 | 194 |
| Rectum, organic stricture of the | 14 | 218 |
| Rectum, cancer of the | 14 | 220 |
| Rectum, vascular tumors of the | 14 | 222 |
| Rectum, ulcer of the | 14 | 223 |
| Rectum, scirrhous, excision of | 15 | 159 |
| Rectum, case of ulcer of the | 15 | 207 |
| Rectum, scirrhus of the, extirpated | 15 | 210 |
| Rectum, ulcer of, cured by bougie | 16 | 256 |
| Rectum, Salmon on prolapsus of | 16 | 369 |
| Rectum, lithotomy by | 17 | 35 |
| Rectum, on inflammation of the | 18 | 329 |
| Rectum, prolapsus of | 19 | 217 |
| Rectum, carcinoma of | 19 | 252 |
| Rectum, on disease of, by Mr. Mayo, | 19 | 289 |
| Rectum, laceration of | 19 | 280 |
| Rectum, ulcer of | 19 | 290 |
| Rectum, protrusion of | 19 | 292 |
| Rectum, bleeding from | 19 | 294 |
| Rectum, strictures of | 19 | 299 |
| Rectum, cancer of | 19 | 303 |
| Red and grey hepatization of the lungs, case | 3 | 519 |
| Red colour for confectionery analyzed | 15 | 204 |
| Reduction of dislocations, accidents following the | 7 | 452 |
| Rees', Mr. translation of Berzelius | 19 | 337 |
| Reflection, ideas derived from | 14 | 296 |
| Reform, medical | 14 | 573 |
| Reform, College of Physicians | 18 | 582 |
| Reform, medical | 19 | 566 |
| Reform, medical, remarks on | 20 | 278 |
| Reform, medical | 20 | 560 |
| Reform meeting in Ireland | 20 | 571 |
| Regulation of lunatic asylums | 9 | 228 |
| Regulations of the College of Surgeons | 5 | 632 |
| Regulations of the College of Surgeons of Edinburgh | 11 | 552 |
| Regulations of the Apothecaries' Company | 12 | 169 |
| Regulations, new, of Navy Med. Board | 20 | 188 |
| Reid, Dr. on epilepsy | 2 | 205 |
| Reid, Dr. on the chlorurets of lime | 8 | 38 |
| Reid, Dr. on hypochondriasis | 13 | 83 |
| Reid, Dr. his elements of chemistry, | 16 | 374 |
| Reid's medical botany | 17 | 387 |
| Remarkable case, by Mr. Muller | 6 | 239 |
| Remittent fever of Africa | 16 | 411 |
| Remittents and intermittents, Dr. Macculloch on | 9 | 49, 289 |
| Remuneration, medical, remarks on, | 2 | 246 |
| Remuneration of general practitioners | 12 | 486 |
| Renal calculi, Mr. Brodie on | 15 | 136 |
| Renal calculi, descent of | 15 | 137 |
| Renton, Dr. on the dysentery of Madeira | 7 | 407 |
| Report of the Small-pox Hospital, by Dr. Gregory | 2 | 551 |
| Report, Mr. Scot's, of the Madras cholera | 3 | 121 |
| Reporting, medical, remarks on | 10 | 249 |
| Reporting from medical societies | 10 | 543 |
| Reporting in Glasgow | 13 | 268 |
| Reports of Indian diseases, Mr. Annesley's | 5 | 342 |
| Reports of medical cases, Dr. Bright's | 15 | 289 |
| Reports on the cholera in Hindostan, | 17 | 57 |
| Reports of the Madras Board | 17 | 408 |
| Republican freedom | 15 | 31 |
| Researches in physiology, by Dr. Blundell | 2 | 400 |
| Researches into the diseases of India, Mr. Annesley's | 8 | 409 |
| Respiration and pulse of children | 3 | 487 |
| Respiration, natural and morbid states of | 4 | 451 |
| Respiration in the new-born child, Mr. Jowett on | 5 | 610 |
| Respiration of cold air in pulmonary diseases | 10 | 559 |
| Respiration, experiments on | 17 | 190 |
| Respiration of aquatic insects | 19 | 230 |
| Respiration of fœtus in utero | 20 | 163 |
| Respiratory organs, affection of, in fever | 12 | 393 |
| Respiratory organs, Mr. Alcock on | 13 | 25 |
| Resuscitation, Dr. Roesler on | 2 | 440 |
| Rete mucosum, anatomy of | 2 | 32 |
| Rete mucosum, blackened by nitrate of silver | 3 | 161 |
| Rete mucosum, description of the | 14 | 141 |

| | Vol. | Page |
|---|---|---|
| Rete mucosum, remarks on the .... | 18 | 352 |
| Retention of urine, cured by puncture | 2 | 477 |
| Retention of urine in pregnancy, case of .................... | 2 | 523 |
| Retention of urine, tinct. ferri. mur. in .................... | 3 | 165 |
| Retention and suppression, difference between.............. | 3 | 165 |
| Retention of urine, *ingenious* mode of treating by *boring* the pubes!! | 5 | 285 |
| Retention of urine—odd false passage | 6 | 576 |
| Retention of urine in fever ........ | 9 | 127 |
| Retention of urine in pregnancy .... | 10 | 103 |
| Retention of urine ............... | 13 | 160 |
| Retention of urine, cases of......... | 14 | 159 |
| Retention of urine, fatal .......... | 14 | 471 |
| Retention of urine, with enlarged prostate .................... | 15 | 191 |
| Retention of urine, Dr. Ducamp on.. | 17 | 49 |
| Retention of urine ............... | 19 | 190 |
| Reticular texture, inflammation of.. | 1 | 210 |
| Retina, phlogosis of the ........... | 6 | 272 |
| Retina, ossification of the ........ | 19 | 215 |
| Retinitis, Mr. Mackenzie on........ | 15 | 66 |
| Retroversio uteri, case of.......... | 6 | 259 |
| Retroversio uteri, cases of ........ | 9 | 539 |
| Retroversio uteri, remarks on ...... | 17 | 315 |
| Retroversio uteri, case of........... | 20 | 156 |
| Reunion of fractured bones ........ | 15 | 193 |
| Revaccination of the Prussian armies, | 20 | 195 |
| Revolution of July, M. Larrey on the, | 16 | 49 |
| Revulsion, Sabatier on the laws of.. | 18 | 87 |
| Revulsion, cases of .............. | 18 | 89 |
| Revulsion, applicability of ........ | 18 | 91 |
| Revulsive bleedings, advantages of.. | 17 | 188 |
| Revulsives, two kinds of .......... | 18 | 88 |
| Rheumatic ophthalmia, Mr. Mackenzie on .................... | 6 | 558 |
| Rheumatic inflammation of the thigh, | 8 | 502 |
| Rheumatic inflammation of knee-joint | 9 | 44 |
| Rheumatic white-swelling, remarks on | 9 | 45 |
| Rheumatic affection of the heart, cases of the ................ | 11 | 559 |
| Rheumatic pericarditis, fatal cases of, | 13 | 183 |
| Rheumatic metastasis to the bladder, | 13 | 226 |
| Rheumatic pericarditis............. | 13 | 428 |
| Rheumatic ophthalmia............. | 14 | 417 |
| Rheumatic affection of the chest.... | 14 | 489 |
| Rheumatism, acute, Dr. Gosse on .. | 1 | 482 |
| Rheumatism treated by a boiling bath | 2 | 302 |
| Rheumatism, observations on its metastases, by Dr. Cox ....... | 2 | 428 |
| Rheumatism, chronic and acute, acupuncture in .............. | 3 | 261 |
| Rheumatism, acute, bark in ....... | 4 | 140 |
| Rheumatism and gout identified! .. | 4 | 141 |
| Rheumatism of the joints, Dr. Chambers on................ | 5 | 566 |
| Rheumatism of the heart cured by acupuncture ................ | 5 | 619 |
| Rheumatism of the temporal muscles........................ | 7 | 113 |
| Rheumatism, Dr. Scudamore on.... | 7 | 337 |
| Rheumatism; Dr. Cazenave on its treatment .................. | 8 | 351 |
| Rheumatism, acute, acid perspiration in .................... | 9 | 176 |
| Rheumatism, metastases of........ | 10 | 92 |
| Rheumatism of the eye, Dr. Macculloch on .................. | 10 | 357 |
| Rheumatism of India, Mr. Henderson on .................... | 10 | 403 |
| Rheumatism, acute, quinine in .... | 13 | 437 |
| Rheumatism, treated by morph. acet. | 17 | 436 |
| Rheumatism of the testis.......... | 19 | 174 |
| Rheumatism, M. Bouillaud on...... | 20 | 254 |
| Rhind, Mr. case of phrenitis ...... | 1 | 299 |
| Rhinoplastic art ................. | 9 | 563 |
| Rhinoplastic operation, case of..... | 12 | 268 |
| Rhinoplastic operation ........... | 17 | 172 |
| Rhinoplastic operation, case of..... | 18 | 188 |
| Rhinoplastic operation ........... | 19 | 489 |
| Rhone and Rhine, the waters of .... | 2 | 443 |
| Rib, carious, excision of a ......... | 13 | 554 |
| Ribes, M. on inflammation of veins, | 4 | 510 |
| Rice, the cause of cholera!!........ | 20 | 180 |
| Richard, Dr. case of hypertrophy of the heart ................ | 7 | 465 |
| Richerand, M. and his *anti-gallican* liberality ................... | 5 | 253 |
| Richmond, Mr. on sea-water in ophthalmia ................. | 5 | 223 |
| Richmond, Mr. on cataract in India, | 5 | 263 |
| Ricord, M. on the venereal ........ | 18 | 388 |
| Ricord, M. on inoculation of syphilis | 19 | 506 |
| Ridicule in hypochondriasis ....... | 13 | 85 |
| Rigby, Dr. his translation of the "Mechanism of Parturition" .. | 11 | 88 |
| Rigidity of the passage during labour | 3 | 419 |
| Rigidity of paralysed members, cause of.................... | 8 | 183 |
| Rival note-takers ............... | 12 | 193 |
| Rivers, salubrity of the banks of.... | 8 | 14 |
| Robert, the Bruce, account of his remains .................... | 4 | 57 |
| Robert, M. on anatomical varieties.. | 9 | 199 |
| Roberton, Mr. on the health of manufacturers ................ | 16 | 77 |
| Robertson, Dr. on dry-cupping .... | 19 | 178 |
| Robertson, Mr. on cynanche laryngea | 20 | 155 |
| Roche, M. on alterations of the blood | 20 | 381 |
| Roger Bacon, historic notice of .... | 15 | 404 |
| Rogerson, Mr. on inflammation .... | 20 | 320 |
| Rolando, M. on the functions of the encephalon.............. | 18 | 522 |
| Roman air, curious effects of ...... | 15 | 282 |
| Rome, hospitals and state of medicine at.................. | 6 | 93 |
| Rome, its past and present climate.. | 8 | 21 |
| Roots of plants, functions of the.... | 17 | 392 |
| Roots, Dr. on fever and rheumatism, | 19 | 539 |
| Roots, Dr. on porrigo ........... | 19 | 539 |
| Roots, Dr. on hepatic abscess ...... | 19 | 540 |
| Rose, Mr. on depositions of pus .... | 9 | 457 |

| | Vol. | Page |
|---|---|---|
| Roseola, caused by cubebs & copaiba | 17 | 185 |
| Rostan, M. on diagnosis and prognosis | 6 | 51 |
| Rot of sheep communicated to man, | 20 | 484 |
| Rouanet on the sounds of the heart, | 20 | 134 |
| Roupell, Dr. on poisons | 20 | 62 |
| Roux, M. on staphyloraphia | 4 | 533 |
| Roux, M. his surgical reports | 5 | 593 |
| Roux, M. his celebrated operation for artificial anus | 9 | 184 |
| Roux, M. on fatal injuries | 19 | 195 |
| Royal exchange of ideas | 9 | 215 |
| Royal College of Surgeons, lectures at | 11 | 225 |
| Rules of diet—Mr. Abernethy's | 9 | 218 |
| Rupia, M. Biett on | 16 | 395 |
| Rupture of the fallopian tube | 1 | 234 |
| Rupture of dropsical ovary, cases of, | 2 | 404 |
| Rupture of the perineum, case of | 3 | 287 |
| Rupture of the uterus, long forceps in | 4 | 150 |
| Rupture of the uterus, Dr. Smith on, | 8 | 359 |
| Rupture of the ileum, cases of | 9 | 498 |
| Rupture of the stomach, produced by vomiting | 9 | 505 |
| Rupture of an aortic aneurism | 13 | 178 |
| Rupture of the spleen, case of | 13 | 247 |
| Rupture of the jejunum | 20 | 142 |
| Rupture of the spleen | 20 | 144 |
| Ruptured uterus, case of recovery after | 4 | 29 |
| Ruptured urethra | 13 | 562 |
| Ruptures, spontaneous, of heart | 18 | 455 |
| Ruspini's styptic, composition of | 14 | 480 |
| Russell, Professor, on affection of bone | 1 | 87 |
| Russell and Barry, Drs. on cholera | 16 | 277 |
| Russell, Mr. on the testicle | 19 | 169 |
| Russian campaign; melancholy picture of it | 5 | 81 |
| Russian rubles, for medical essayists, | 14 | 192 |
| Russian embassy on cholera | 16 | 489 |
| Ruth, Mr. his curious case of Cæsarean section | 3 | 285 |
| Rutter, Dr. his reclamation | 4 | 300 |
| Ryall, Mr. Isaac, on purulent ophthalmia | 2 | 220 |
| Ryall, Mr. on the nitrate of silver in affections of the eye | 9 | 182 |
| Ryan, Dr. on angina pectoris | 1 | 203 |
| Ryan, Dr. and his pupils | 15 | 563 |
| Ryan, Dr. on midwifery | 17 | 114 |
| Rynd, Dr. on affections of the fingers and toes | 14 | 214 |

### S

| | Vol. | Page |
|---|---|---|
| Sabatier on the laws of revulsion | 18 | 87 |
| Sac, thick, in femoral hernia | 20 | 204 |
| Sacchi on the goitre | 19 | 498 |
| St. George's Hospital, inquest at | 3 | 583 |
| St. John Long's quackery | 10 | 245 |
| Saint Helena, Napoleon at, by M. Hereau | 10 | 425 |
| Saint Helena, topography of | 10 | 425 |
| St. John Long's cures and casualties, | 11 | 287 |
| St. John Long, inquest on | 13 | 520 |
| St. John Long, remarks on | 13 | 572 |
| St. John Long again upon the scene, | 14 | 241 |
| St. John Long, his liniment | 14 | 257 |
| St. John Long and Dr. Ramadge | 15 | 178 |
| St. Petersburgh, cholera at | 16 | 214 |
| St. Vitus' dance, treatment of, in France | 18 | 466 |
| St. George's Hospital report | 18 | 564 |
| Salicine for ague | 20 | 482 |
| Saline impregnation of the blood | 13 | 219 |
| Saline treatment of Dr. Stevens | 17 | 331 |
| Saline treatment, results of | 17 | 333 |
| Saline treatment, analysis of those results | 17 | 334 |
| Salines, powers of, in fever | 17 | 329 |
| Saliva, state of in mercurialization | 4 | 490 |
| Salivary fistula, treatment of | 10 | 226 |
| Salivary calculus, Mr. Bell on | 11 | 409 |
| Salivation fatal, a case | 7 | 409 |
| Salivation from *bread* pills | 11 | 167 |
| Salivation arrested by iodine | 20 | 205 |
| Salmon, Mr. on prolapsus recti | 16 | 369 |
| Salt the grand digestive! | 11 | 254 |
| Salt, adulteration of | 19 | 232 |
| Salubrity of the Neilgherries | 13 | 309 |
| Salutarium at Oban | 19 | 160 |
| Sand, red in the urine | 15 | 131 |
| Sand, white, in the urine | 15 | 134 |
| Sand-rock spring, salts of the | 2 | 551 |
| Sanders, Dr. on phthisis | 1 | 256 |
| Sanders, Mr. H. on puerperal fever, | 2 | 244 |
| Sanders, Dr. his letter to Sir H. Halford | 16 | 297 |
| Sane or insane | 12 | 509 |
| Sanguineous apoplexy, a most marvellous case of | 4 | 267 |
| Sanguineous tumor, some anomalous cases of | 7 | 159 |
| Sanguineous tumor, ligature of the artery for | 7 | 161 |
| Sangrado practice revived | 3 | 299 |
| Sanity and insanity, affinities of | 13 | 298 |
| Sanson, M. on amputation | 8 | 461 |
| Saphena vein, division and excision of, | 10 | 162 |
| Sarcocele extirpated—tetanus | 8 | 537 |
| Sarcocele, case of—castration | 9 | 277 |
| Sarsaparilla, Mr. Pope on | 1 | 47 |
| Sarsaparilla, effects of | 19 | 478 |
| Satyriasis, case of | 19 | 238 |
| Saxe-Weimar, medical regulations in | 19 | 513 |
| Scabies, M. Biett on | 16 | 392 |
| Scald of mouth, Bouillaud on | 19 | 195 |
| Scalds, Dr. M'Farlane on | 19 | 529 |
| Scalp, encysted tumors of the | 10 | 173 |
| Scalp, diffuse inflammation of the | 12 | 172 |
| Scalp, deceptive depression in the | 13 | 558 |
| Scalp, diffuse inflammation of the | 14 | 183 |
| Scalp, gun-shot wound of the | 15 | 154 |
| Scalp, on wounds of the | 15 | 532 |
| Scalp, on contusions of the | 15 | 533 |
| Scalp, on erysipelas of the | 15 | 533 |
| Scalp-wound, case of | 14 | 280 |

| | Vol. | Page |
|---|---|---|
| Scalp-wound, erysipelas of the head and face | 10 | 579 |
| Scalp-wounds, cases of, with remarks | 6 | 191 |
| Scalp-wounds, and their consequences | 14 | 178, 235 |
| Scalp-wounds, cases of | 14 | 179 |
| Scalp-wounds, followed by erysipelas, | 14 | 180 |
| Scalvanti, Dr. on abscess of cerebellum | 19 | 509 |
| Scaly eruptions, mercury in | 3 | 473 |
| Scarifications, value of | 13 | 546 |
| Scarlatina, followed by empyema | 13 | 447 |
| Scarlet fever, Mr. Alcock on | 13 | 29 |
| Scarlet fever, hints on the | 17 | 176 |
| Scarlett's, Sir Jas. admirable speech, | 10 | 280 |
| Scarpa's rare cases in surgery | 6 | 221 |
| Scarpa's opinions on aneurism | 13 | 358 |
| Scents in Rome | 15 | 282 |
| Schenk's case of Cæsarian section | 4 | 478 |
| Schloss, Mr. his anatomical models | 17 | 564 |
| School of medicine, complete, at Cambridge | 9 | 522 |
| Sciatica, cured by turpentine | 6 | 577 |
| Sciatica, Dr. Scudamore on | 7 | 354 |
| Sciatica, Dr. Macculloch on | 10 | 60 |
| Sciatica, cured by acupuncture | 14 | 477 |
| Sciatica treated by acupuncture | 15 | 468 |
| Sciatica, cured by bleeding | 17 | 436 |
| Sciatica, treatment of | 19 | 196 |
| Scientific pursuits, moderation in | 13 | 88 |
| Scirrhoma, Dr. Carswell on | 19 | 187 |
| Scirrhous uterus, unhappy case of | 3 | 414 |
| Scirrhous testicle in the groin | 7 | 239 |
| Scirrhous pancreas, Mr. King's case, | 7 | 493 |
| Scirrhous pylorus, case of | 12 | 164 |
| Scirrhous disease of the testis | 14 | 9 |
| Scirrhous rectum, excision of | 15 | 159 |
| Scirrhus of the pylorus | 1 | 191 |
| Scirrhus of stomach, symptoms of | 5 | 208 |
| Scirrhus, M. Lisfranc's report on | 5 | 598 |
| Scirrhus, constitutional predisposition to | 6 | 552 |
| Scirrhus of the stomach | 7 | 73 |
| Scirrhus of the male breast | 10 | 472 |
| Scirrhus, structures affected by the | 11 | 388 |
| Scirrhus, structure of | 11 | 389 |
| Scirrhus of the ovarium | 12 | 421 |
| Scirrhus of rectum, M. Lisfranc on | 13 | 148 |
| Scirrhus of the lung, case of | 13 | 520 |
| Scot, Mr. his report of cholera | 3 | 121 |
| Scotland, Medical Provident Institution of | 12 | 484 |
| Scotland Medical Institution | 15 | 453 |
| Scott, Dr. his case of cystitis hepatica | 3 | 222 |
| Scott, Dr. on puncturing the brain in hydrocephalus | 3 | 306 |
| Scott, Mr. on diseases of the joints | 9 | 307 |
| Scott, Mr. on lavements | 12 | 499 |
| Scott, Mr. on extirpation of the upper jaw | 19 | 281 |
| Scott, Sir Walter, his character | 19 | 328 |
| Scouler, Dr. on production of worms, | 13 | 221 |

| | Vol. | Page |
|---|---|---|
| Scout sloop of war, fever in | 2 | 12 |
| Scoutteten on the peritoneum | 1 | 199 |
| Scrofula, Dr. Alison on | 2 | 98 |
| Scrofula, M. Dupuytren's practice in, | 3 | 358 |
| Scrofula, state of digestive organs in, | 3 | 483 |
| Scrofula, Mr. Rennie on | 3 | 565 |
| Scrofula, cases of, treated by iodine, | 4 | 11 |
| Scrofula, M. Dupuytren's treatment of | 12 | 554 |
| Scrofula, M. Lugol on iodine in | 16 | 109 |
| Scrofula treated by iodine | 16 | 117 |
| Scrofula, treatment of, by iodine | 17 | 169 |
| Scrofula of bones, cases of | 17 | 439 |
| Scrofula, treatment of | 19 | 203 |
| Scrofulous inflammation of testis | 13 | 403 |
| Scrofulous ophthalmia | 14 | 409 |
| Scrofulous tubercles | 16 | 119 |
| Scrofulous ophthalmia, iodine in | 16 | 120 |
| Scrofulous eruptions | 16 | 121 |
| Scrotal tumor, curious instance of | 3 | 227 |
| Scrotal hernia, fatal case of | 7 | 246 |
| Scrotum, sloughing of the, curious cases of | 11 | 270 |
| Scrotum, morbid itching of the | 12 | 493 |
| Scrotum, *anomalous* affection of the, | 12 | 498 |
| Scrotum, elephantiasis of the | 12 | 505, 535 |
| Scrotum, cancer of the | 14 | 29 |
| Scrotum, enlargement of | 19 | 172 |
| Scudamore, Dr. on the blood | 1 | 137 |
| Scudamore, Dr. on colchicum in gout | 4 | 44 |
| Scudamore, Dr. on rheumatism | 7 | 337 |
| Scudamore, Sir C. on phthisis | 14 | 352 |
| Scudamore, Sir Charles, on iodine | 20 | 420 |
| Scurvy, its appearance & symptoms among the Millbank prisoners | 3 | 6 |
| Scurvy, nitrate of potass in | 12 | 483 |
| Scurvy, Stevens' discoveries in | 17 | 330 |
| Sea-scurvy not always cured by lemon-juice | 1 | 231 |
| Sea-water-baths to be established | 3 | 601 |
| Sea-sickness, as a *methodus medendi*, | 4 | 36 |
| Sea-water in ophthalmia, Mr. Richmond on | 5 | 222 |
| Sea-sickness, some hints on | 5 | 251 |
| Searle, Mr. on compression | 1 | 516 |
| Searle, Mr. on cholera | 13 | 375 |
| Searle, Mr. on cholera | 16 | 211 |
| Seasons, effects of, on animals | 18 | 17 |
| Sebaceous follicles, disease of the | 17 | 381 |
| Secale cornutum, Dr. Hosack on | 2 | 59 |
| Secale cornutum, Mr. Haslam on | 6 | 517 |
| Secale cornutum, Dr. Dewees on the use of | 9 | 209 |
| Secale cornutum, Dr. Beatty on the, | 20 | 438 |
| Secale cornutum, Dr. Negri on | 20 | 450 |
| Secondary hæmorrhage, cases of | 8 | 477 |
| Secondary hæmorrhage, Mr. Bell on, | 8 | 564 |
| Secondary hæmorrhage, case of, with remarks | 9 | 260 |
| Secondary hæmorrhage, another instance of | 9 | 261 |

| | Vol. | Page |
|---|---|---|
| Secondary hæmorrhage, remarkable kind of | 10 | 470 |
| Secondary hæmorrhage, amputation, | 13 | 277 |
| Secondary hæmorrhage, case of | 20 | 250 |
| Secondary symptoms, cases of | 20 | 537 |
| Secrecy, in the treatment of female complaints | 3 | 176 |
| Secret, grand, for a successful and honorable career | 12 | 147 |
| Secret remedies | 17 | 161 |
| Section and ligature of the pneumogastric nerves | 7 | 410 |
| Section, Cæsarian, on a dead woman, | 12 | 521 |
| Section of trachea and œsophagus | 13 | 246 |
| Sedatives in puerperal mania | 11 | 369 |
| Seeds, Dr. his remedy for ophthalmia | 16 | 607 |
| Seiler, Dr. on the uterus and ovum | 18 | 356 |
| Seiler, Dr. on malformation of the eyes | 20 | 370 |
| Semi-decussation of the optic nerves, | 3 | 201 |
| Semilunar valves, diseases of the | 9 | 397 |
| Semilunar aortic valves ruptured | 14 | 197 |
| Seminal emissions, on | 19 | 171 |
| Senna leaves, account of | 3 | 157 |
| Sensation and volition, M. Magendie, | 9 | 231 |
| Sense, nerves of, paralysis of | 20 | 213 |
| Senses, gradual abolition of all of the, | 9 | 440 |
| Senses, fallacious evidence of the | 14 | 298 |
| Sensibility, various kinds of | 6 | 71 |
| Separation without dissension | 15 | 526 |
| Septum narium, diseases of | 19 | 454 |
| Sero-enteritis, symptoms of | 11 | 101 |
| Sero-enteritis, sub-acute | 11 | 102 |
| Serous effusion in old persons | 13 | 171 |
| Serous cysts, Mr. Lawrence on | 18 | 342 |
| Serum, in dropsical uterine; Dr. Wells' statement | 4 | 407 |
| Seton in a case of bronchocele | 7 | 263 |
| Seton in cases of ruptured tendon | 7 | 451 |
| Seton successful in ununited fracture, | 11 | 468 |
| Seton for subcutaneous nævus | 13 | 506 |
| Seton, use of, in nævi | 20 | 174 |
| Sewel, Mr. on contagious glanders | 2 | 249 |
| Sexual desire, is it seated in the cerebellum? | 9 | 117 |
| Sexual diseases, Mr. Thorn on | 15 | 412 |
| Seymour, Dr. on abdominal tumors, | 9 | 399 |
| Seymour, Dr. on insanity | 18 | 139 |
| Shampooing and rubbing considered, | 3 | 382 |
| Shaw, Mr. on spinal diseases | 1 | 303 |
| Shaw, Mr. on stricture of the urethra | 1 | 475 |
| Shaw, Mr. on dissection-wounds | 3 | 249 |
| Shaw, Mr. on lateral curvature, &c. | 3 | 366 |
| Shaw, Mr. on lithotomy | 4 | 540 |
| Shaw, Mr. obituary of | 7 | 581 |
| Shearman, Dr. on debility | 1 | 391 |
| Shearman, Dr. on water on the brain | 4 | 432 |
| Sheet-lead for ulcers | 9 | 451 |
| Sheldrake on dancing | 10 | 489 |
| Sheldrake, Mr. on personal appearance | 10 | 554 |
| Shoulder-joint, excision and amputation at | 18 | 274 |

| | Vol. | Page |
|---|---|---|
| Shoulder-joint, amputation of | 15 | 513 |
| Shoulder-presentations, Dr. Ramsbotham on | 17 | 292 |
| Shoulder-presentations, cases of | 17 | 294 |
| Shoulders, in incipient curvature | 3 | 367 |
| Shute, Dr., principles of medical science | 7 | 358 |
| Sickness, fatal, from pregnancy | 8 | 149 |
| Siebold, Dr. his case of extirpated uterus | 3 | 264 |
| Sierra Leone, Mr. Boyle on | 16 | 409 |
| Sigmoid flexure of the colon, stricture of | 10 | 95 |
| Sigmoid flexure of colon, stricture of, | 13 | 150, 228 |
| Signs of the times, or epidemics of the day | 8 | 227 |
| Signs of the times—" decency and dulness" | 9 | 240 |
| Signs of pregnancy | 20 | 96 |
| Silk-worm gut for ligatures | 6 | 249 |
| Silk-worm gut ligature | 7 | 253 |
| Silver, nitrate of, in cynanche laryngea | 20 | 155 |
| Simple and compound fractures | 12 | 566 |
| Simplon, night on the | 14 | 453 |
| Simpton, Mr. on clots in the bladder, | 20 | 183 |
| Simulated diseases | 1 | 508 |
| Simulated pregnancy | 20 | 119 |
| Sinapisms to the mammæ, in amenorrhœa | 20 | 171 |
| Singleton's golden ointment | 14 | 285 |
| Sir Matthew Tierney's powder | 9 | 456 |
| Sir Astley Cooper on the testis | 13 | 385 |
| Sizy blood, on the nature of | 1 | 139 |
| Skeletons, best mode of preparing | 20 | 150 |
| Skin, Mr. Chevalier on | 2 | 29 |
| Skin, Mr. Plumbe on | 2 | 29 |
| Skin, M. Alibert on | 2 | 29 |
| Skin, anatomy of the | 12 | 60 |
| Skin, Cazenave on diseases of the | 16 | 382 |
| Skin, Biett on diseases of the | 17 | 360 |
| Skin, Dr. Wood on the | 18 | 350 |
| Skull, compound fracture of the | 12 | 276 |
| Sloughing of the bladder, post partum | 1 | 235 |
| Sloughing ulcer, its progress & origin, | 3 | 465 |
| Sloughing ulcer and chancre, their distinctions | 3 | 478 |
| Sloughing ulceration, Mr. Traver's cases of | 5 | 552 |
| Sloughing ulceration, Mess^rs Green and Rose on | 5 | 553 |
| Sloughing chancre | 7 | 531 |
| Sloughing ulcers, Mr. Langstaff on | 10 | 404 |
| Sloughing of an aneurismal sac | 13 | 362 |
| Sloughing of penis | 13 | 436 |
| Sloughing chancres, cases of | 15 | 229 |
| Sloughing ulcer of the nose, case of | 15 | 543 |
| Sloughy abscess of the thigh and knee-joint, cases of | 11 | 456 |
| Sloughy abscess of the leg, report on, | 13 | 281 |
| Small-pox, observations on the treatment of | 11 | 148 |

| | Vol. | Page |
|---|---|---|
| Small-pox, Mr. Alcock on | 13 | 27 |
| Small-pox, Dr. Otto on | 19 | 481 |
| Small-pox, Mr. George on | 20 | 59 |
| Smell and sight in birds of prey | 9 | 538 |
| Smilax aspera, use of | 19 | 477 |
| Smith, Dr. on infanticide | 1 | 337 |
| Smith, Dr. his arrangement of miasms | 3 | 71 |
| Smith, Dr. his work on medical evidence | 3 | 169 |
| Smith, Dr. Gordon, his reclamation, | 3 | 596 |
| Smith, Mr. his Prize Hospital Report | 6 | 601 |
| Smith, Mr. on aneurism of the brachial artery | 11 | 157 |
| Smith, Dr. on fever | 12 | 337, 385 |
| Smith, Dr. his letter to the editor | 13 | 230 |
| Smoking, is it pernicious? | 18 | 489 |
| Smyrna, remarkable fever there | 5 | 637 |
| Smyrna, algid fever of | 16 | 607 |
| Snell, Mr. on obturateurs | 4 | 598 |
| Snuff-taking, Dr. Kinglake on | 2 | 250 |
| Soap cerate, Mr. Fergusson on | 16 | 590 |
| Society, Obstetric, proceedings of | 13 | 241 |
| Softening of the stomach | 2 | 173 |
| Softening of the lung, grey, case of, | 3 | 520 |
| Softening of brain, Dr. Abercrombie on | 9 | 80 |
| Softening of the stomach, Prof. Autenrieth on | 9 | 464 |
| Softening of the stomach, memoir on, | 11 | 530 |
| Soils productive of malaria | 8 | 7 |
| Solar heat, influence of | 12 | 335 |
| Soldiers, Austrian, strictness in discharge of | 17 | 260 |
| Somascetics, use of | 19 | 504 |
| Somerville, Dr. his circulars on the Anatomy Act | 17 | 570 |
| Sonderland's experiments on cow-pox repeated | 20 | 208 |
| Sore throat peculiar to children, Dr. Hamilton on | 6 | 223 |
| Soul, seat of the | 12 | 461 |
| Sounding for stone, death from | 15 | 370 |
| Sounding for stone, inflamed bladder from | 15 | 375 |
| Sounds of heart variously explained, | 20 | 133 |
| Sour bread, a supposed cause of goitre | 2 | 442 |
| South America, medical science in | 3 | 313 |
| Southern Coast of England, Dr. Harwood on | 10 | 24 |
| Spa, Beulah, account of | 16 | 554 |
| Spasm of muscular tubes, Dr. Monro on | 5 | 500 |
| Spasm of the bladder in lithotomy | 6 | 265 |
| Spasm of the stomach, Dr. Macfarlane on | 11 | 489 |
| Spasm of the glottis, Dr. Marsh on | 14 | 366 |
| Spasm of the colon, Mr. Lyon on | 15 | 207 |
| Spasm of the glottis, Mr. Fletcher on, | 15 | 378 |
| Spasm of the glottis, cases of | 15 | 379 |
| Spasm of the glottis, treatment of | 15 | 384 |
| Spasmodic stricture of the anus | 2 | 300 |
| Spasmodic pains in the joints | 3 | 386 |
| Spasmodic "crooping noise" in children; remarks on it | 5 | 165 |
| Spasmodic dyspnœa cured by purging, | 14 | 163 |
| Spasmodic cholera, Dr. Smith on | 14 | 347 |
| Spasmodic cholera, prevention of | 15 | 463 |
| Spasms in children, a peculiar kind of | 2 | 452 |
| Specific diseases and medicines, Mr. Allan on | 5 | 541 |
| Specks on cornea, M. Dupuytren's treatment | 12 | 539 |
| Spectral illusion, remarkable case of | 9 | 535 |
| Spectral illusions of a medical gentleman | 15 | 205 |
| Spectral illusions, Sir D. Brewster on | 17 | 473 |
| Spectral illusions, his theory of | 17 | 474 |
| Spectral illusions, remarks on the theory | 17 | 477 |
| Speculations on the spleen | 9 | 219 |
| Speculum, facts relating to the use of | 18 | 389 |
| Speech, sudden loss of | 1 | 180 |
| Spermatic cord, hydrocele of, with peritonitis | 11 | 178 |
| Spermatic artery, ligature of, for varicocele | 11 | 256 |
| Spermatic cord, anatomy of the | 14 | 383 |
| Sphacelated urethra, supposed regeneration of | 6 | 220 |
| Spina ventosa, Mr. Bell on | 10 | 416 |
| Spina bifida, Mr. Stafford on | 17 | 2 |
| Spina bifida, cases of | 17 | 4 |
| Spina bifida, treatment of | 17 | 5 |
| Spina ventosa | 20 | 523 |
| Spinal arachnitis | 1 | 32 |
| Spinal diseases | 1 | 303 |
| Spinal consumption | 1 | 456 |
| Spinal disease, fatal case of | 7 | 247 |
| Spinal disease, Mr. Auchincloss on | 8 | 553 |
| Spinal diseases, Dr. Harrison on | 9 | 87 |
| Spinal disease, extensive, case of | 9 | 152 |
| Spinal irritation, Dr. Brown on | 9 | 219 |
| Spinal neuralgia, instructive case of, | 10 | 244 |
| Spinal disease, unsuspected | 10 | 462 |
| Spinal cord, ramollissement of the | 12 | 487 |
| Spinal irritation, Mr. Whatton on | 15 | 458 |
| Spinal irritation, symptoms of | 15 | 460 |
| Spinal irritation, treatment of | 15 | 462 |
| Spinal irritation, case of | 15 | 565 |
| Spinal irritation, Dr. Corrigan on | 15 | 182 |
| Spinal nerves, Muller's experiments on | 20 | 225 |
| Spinal-marrow, on diseases of | 1 | 1 |
| Spinal-marrow, on functions of | 1 | 2 |
| Spinal-marrow, on compression of | 1 | 13 |
| Spinal-marrow, construction of | 2 | 151 |
| Spinal-marrow, case of great destruction of | 4 | 180 |
| Spinal-marrow, inflammation of the, | 7 | 464 |
| Spinal-marrow, division of, in the back | 9 | 442 |
| Spinal-marrrow, apoplexy of the | 12 | 88 |
| Spinal-marrow, irritation of the | 12 | 113 |
| Spine, Dr. Dods on the | 1 | 92 |
| Spine, Mr. Bampfield on | 2 | 112 |

| | Vol. | Page |
|---|---|---|
| Spine, fracture of, trepanned ...... | 6 | 601 |
| Spine, injuries of, at St. Thomas's .. | 6 | 601 |
| Spine, case of fracture of the ...... | 6 | 605 |
| Spine, fractures of the ............ | 9 | 473 |
| Spine tinker, the ............... | 10 | 256 |
| Spine, pain of the, in hysteria ...... | 13 | 35 |
| Spine, new mode of opening the .... | 13 | 570 |
| Spine, cases of injury of the ...... | 14 | 226 |
| Spine, disease of the.............. | 14 | 518 |
| Spine, fractured, paralysis from . .. | 14 | 519 |
| Spine, Mr. Beale on distortions of .. | 15 | 418 |
| Spine, injuries of the.............. | 17 | 8 |
| Spine, fractures of the ............ | 17 | 14 |
| Spine, dislocations of the .......... | 17 | 15 |
| Spine, cancellous structure of...... | 17 | 17 |
| Spine, ulceration, &c. of .......... | 17 | 19 |
| Spinitis, Dr. Potain's case of ...... | 8 | 185 |
| Spinner's phthisis, Dr. Kay on .... | 15 | 477 |
| Spirits, effects of, upon animals .... | 3 | 184 |
| Spleen, Dr. Vetch on .............. | 1 | 460 |
| Spleen, two cases of loss of...... .. | 2 | 403 |
| Spleen, rupture of, in fevers ....... | 5 | 283 |
| Spleen, fatal disease of............ | 9 | 435 |
| Spleen, pathology of the .......... | 10 | 351 |
| Spleen, diseases of the ............ | 11 | 314 |
| Spleen, diseased, treatment of the .. | 11 | 317 |
| Spleen reduced into a pulpy mass .. | 12 | 85 |
| Spleen, Dr. Raikem on inflammation of the.................. | 12 | 252 |
| Spleen, fatal hæmorrhage from the.. | 13 | 227 |
| Spleen, apoplexy of the .......... | 13 | 247 |
| Spleen, Sir Anthony Carlisle on the, | 13 | 486 |
| Spleen, a warming-pan for stomach, | 13 | 487 |
| Spleen, Mr. Dobson on the ........ | 13 | 531 |
| Spleen, a reservoir for the stomach.. | 13 | 531 |
| Spleen, experiments on the .... ... | 13 | 531 |
| Spleen, physiology of the .......... | 15 | 48 |
| Spleen, softening of, Hackman on .. | 17 | 257 |
| Spleen, rupture of................ | 20 | 144 |
| Splenitis, case of ................ | 12 | 83 |
| Sponge-tent, as a dilator of the female urethra....... ........ | 7 | 567 |
| Spontaneous salivation in syphilis .. | 3 | 480 |
| Spontaneous combustion, M. Hellis case of .................. | 5 | 543 |
| Spontaneous cure of femoral aneurism, | 11 | 164 |
| Sprague, Mr. formula for porrigo .. | 1 | 224 |
| Springs, mineral, Dr. Gairdner on .. | 19 | 28 |
| Spurgin's, Dr. case of poisoned wound | 5 | 370 |
| *Spurious Cæsarian section*, remarks on the ..... ................ | 4 | 25 |
| Spurzheim, Dr. his view of insanity, | 3 | 232 |
| Spurzheim, Dr. on phrenology and physiognomy................ | 5 | 468 |
| Spurzheim, Dr. death of .......... | 18 | 532 |
| Spurzheim, Dr. phrenological developement of................. | 19 | 152 |
| Sputa of bronchitis .............. | 3 | 506 |
| Squinting, modes of obviating...... | 5 | 161 |
| Stafford, Mr. on strictures of urethra, | 11 | 216 |
| Stafford, Mr. on ulcers.... ....... | 12 | 37 |
| Stafford, Mr. on injuries of the spine, | 17 | 1 |

| | Vol. | Page |
|---|---|---|
| Stammering, nature and causes of .. | 9 | 193 |
| Stammering, Dr. Palmer on........ | 12 | 304 |
| Stammering cured by purgatives.... | 14 | 209 |
| Staining of blood-vessels, M. Laennec on ..................... | 8 | 488 |
| Stanley, Mr. his prosecution ...... | 8 | 527 |
| Stanley, Mr. on lithotomy ........ | 12 | 69 |
| Stanley, Mr. on osseous union of a fractured cervix femoris ...... | 20 | 170 |
| Staphyloma cured by tapping ...... | 2 | 475 |
| Staphyloma, treatment of ......... | 19 | 156 |
| Staphyloraphia .................. | 4 | 533 |
| Staphyloraphy, Dr. Dieffenbach's improvements ................ | 5 | 188 |
| State of the blood in cholera ....... | 3 | 129 |
| Statistics, medical, Dr. Hawkins on.. | 11 | 420 |
| Statistics, moral, of France........ | 19 | 247 |
| Statistics, medical................. | 20 | 234 |
| Stays injurious, and yet useful, in distortion ................... | 3 | 377 |
| Stedman, Dr. on the dandy fever at Santa Cruz ................ | 10 | 205 |
| Steevens, Dr. on the blood ........ | 13 | 217 |
| Steinmetz, clinical observations . .. | 19 | 501 |
| Steno-cardia, or angina pectoris .... | 7 | 430 |
| Stephens, Mr. on hernia .......... | 11 | 109 |
| Stephens, Mr. his views of hernia .. | 11 | 546 |
| Stephens, Dr. his ligature of the internal iliac......... ...... | 14 | 57 |
| Stephenson and Churchill's Medical Botany................ | 8 | 117 |
| Stephenson, Dr. his medical zoology, | 15 | 126 |
| Sterility, Dr. Harrison on ...... . | 14 | 470 |
| Sterility, cure of .................. | 19 | 202 |
| Sterility, curious cases of.......... | 20 | 159 |
| Sternum, case of fracture of ...... | 17 | 536 |
| Stethoscope, in diagnosis of aneurisms ...................... | 1 | 183 |
| Stethoscope, testimony to its value, | 3 | 495 |
| Stethoscope, diagnostic powers of... | 4 | 571 |
| Stethoscope, Dr. Scudamore on its use, &c...................... | 5 | 73 |
| Stethoscope; prejudiced opposers of, | 8 | 114 |
| Stethoscope, remarks on its opposers, | 9 | 212 |
| Stethoscope, illustrations of its use, | 9 | 241 |
| Stethoscope in supposed hydrothorax, | 9 | 513 |
| Stethoscope, Dr. Hastings on the .. | 11 | 497 |
| Stethoscope, new .. ............. | 13 | 563 |
| Stevens, Dr. criticised by Dr. Hackett | 16 | 289 |
| Stevens, Dr. on the blood ......... | 17 | 321 |
| Stevens, Dr. his modesty .......... | 17 | 327 |
| Stevenson, Mr. on cataract ........ | 1 | 396 |
| Stevenson, Mr. on the absorbent practice in cataract. ......... | 4 | 234 |
| Stevenson, Mr. on deafness ....... | 10 | 45 |
| Stewart, Dr. on phthisis .......... | 1 | 256 |
| Stewart, Dr. on education of children, | 9 | 146 |
| Stibiated mercurial ointment ...... | 1 | 230 |
| Stiff knee, recovery from, in fever .. | 18 | 468 |
| Still-born children, auscultation applied to ................ | 20 | 164 |
| Stimulants in irritative fever....... | 4 | 87 |

| | Vol. | Page |
|---|---|---|
| Stimulants, external, Dr. Hancock on | 18 | 145 |
| Stimuli in concussion | 13 | 561 |
| Stirling, Mr. surgical cases by | 19 | 249 |
| Stoker, Dr. on pathology of the fluids | 1 | 144 |
| Stoker, Dr. on fever | 2 | 1 |
| Stoker, Dr. his observations on fever, &c. | 11 | 337 |
| Stokes, Dr. on the use of the stethoscope | 4 | 449 |
| Stokes, Dr. on large doses of opium, | 18 | 156 |
| Stokes, Dr. on gastritis | 19 | 175 |
| Stokes, Dr. on dyspepsia | 19 | 175 |
| Stokes, Dr. on phthisis | 20 | 257 |
| Stokes, Dr. on pleuritis biliosa | 20 | 257 |
| Stokes, Dr. on dysphagia | 20 | 257 |
| Stokes, Dr. on diptherite | 20 | 258 |
| Stokes, Dr. on laryngeal phthisis | 20 | 258 |
| Stokes, Dr. on dropsy | 20 | 259 |
| Stokes, Dr. on sympathy | 20 | 259 |
| Stokes, Dr. on delirium tremens, &c. | 20 | 457 |
| Stomach, curious disease of | 2 | 170 |
| Stomach, tumors in | 2 | 174 |
| Stomach—prevalent affections of this organ | 4 | 372 |
| Stomach, perforation of, by a bullet, a case | 5 | 38 |
| Stomach, case of scirrhus of | 5 | 206 |
| Stomach of a Broussaian, its marvellous properties | 5 | 500 |
| Stomach ; morbid affections of its follicles | 6 | 153 |
| Stomach; diseases of its cellular coats | 6 | 156 |
| Stomach; cases of enormous distention of the | 6 | 159 |
| Stomach, softening and perforation of | 6 | 240 |
| Stomach, case of chronic ulcer in the | 6 | 493 |
| Stomach, effects of improper stimuli, | 7 | 50 |
| Stomach affection, remarkable case of | 7 | 84 |
| Stomach, hypertrophy of the | 7 | 221 |
| Stomach, influence of, upon the mind | 8 | 121 |
| Stomach and bowels, inflammation of its mucous membrane | 8 | 361 |
| Stomach ; its influence on the brain, | 8 | 572 |
| Stomach, inflammation of the. | 9 | 353 |
| Stomach, perforation of the | 9 | 465 |
| Stomach, scirrhus of the | 10 | 121 |
| Stomach and duodenum, severe affection of the | 10 | 211 |
| Stomach, Dr. Abercrombie on the diseases of the | 10 | 328 |
| Stomach, inflammation and ulceration of the | 10 | 330 |
| Stomach, organic diseases of the | 10 | 336 |
| Stomach, functional diseases of the | 10 | 338 |
| Stomach and intestines, Dr. Abercrombie on the | 11 | 1 |
| Stomach, &c. inflammation of, producing hypochondriasis | 11 | 150 |
| Stomach, irritation of the, simulating apoplexy | 12 | 251 |
| Stomach and liver, fungus hæmatodes of | 12 | 563 |

| | Vol. | Page |
|---|---|---|
| Stomach, medullary sarcoma of the | 12 | 223 |
| Stomach and intestines, pathology of, | 13 | 223 |
| Stomach, on organic disease of the | 13 | 319 |
| Stomach, hypertrophy of the | 13 | 491 |
| Stomach, case of perforation of the, | 14 | 364 |
| Stomach, cyst, communicating with, | 15 | 568 |
| Stomach, cancer of, mistaken | 16 | 231 |
| Stomach, organic disease of | 18 | 469 |
| Stomach, ulceration of the | 18 | 477 |
| Stomach-pump, Mr Bryce's new one | 4 | 264 |
| Stomach-pump, new application of | 20 | 183 |
| Stone, Mr. on polypus uteri | 6 | 210 |
| Stone in bladder, high operation for, | 10 | 569 |
| Stone, Dr. King on treatment of | 17 | 24 |
| Stoop, erroneous modes of treating it | 3 | 379 |
| Stopping of carious teeth, remarks on | 6 | 143 |
| Strabismus, Mr. Mackenzie on | 15 | 523 |
| Strabismus, causes of | 15 | 523 |
| Strabismus, treatment of | 15 | 524 |
| Stramonium, Drs. Wendt and Elliotson on | 9 | 281 |
| Strangulated hernia, case of | 6 | 609 |
| Strangulated intestine, gastrotomy for | 7 | 224 |
| Strangulated hernia | 7 | 472 |
| Strangulated hernia, cases of | 7 | 523 |
| Strangulated hernia, Mr. Brodie's cases of | 8 | 449 |
| Strangulated hernia, case of | 8 | 451<br>452 |
| Strangulated femoral hernia, fatal | 9 | 558 |
| Strangulated inguinal hernia | 10 | 234 |
| Strangulated hernia, cases of | 11 | 176 |
| Strangulated hernia rapidly fatal | 15 | 153 |
| Strangulated hernia, case of | 17 | 156 |
| Strangulated hernia, Mr. Key on | 20 | 80 |
| Strangulation, internal, remarkable case of | 11 | 247 |
| Strangulations, operation for | 13 | 544 |
| Strangulations, internal | 13 | 568 |
| Stratten, Dr. his notes on Hahnemann | 19 | 429 |
| Strength, remarkable feats of | 17 | 481 |
| Stricture of the urethra—Mr. Arnott | 1 | 474 |
| Stricture of the urethra, Mr. Shaw | 1 | 475 |
| Stricture of the ileum, Dr. Moleson's case of | 6 | 83 |
| Stricture of the urethra, Mr. Macilwain on | 7 | 69 |
| Stricture of the œsophagus; hospital of surgery | 8 | 169 |
| Stricture of the rectum, Mr. Salmon's essay on | 8 | 325 |
| Stricture of the lacrymal duct, Mr. Teale on | 8 | 460 |
| Stricture, death from | 10 | 204 |
| Stricture, fatal, of the colon | 12 | 504 |
| Stricture of the rectum | 13 | 193 |
| Stricture of the œsophagus | 13 | 200 |
| Stricture, treatment of by caustic | 17 | 50 |
| Stricture, instruments used in | 17 | 51 |
| Stricture, Mr. Guthrie on | 20 | 548 |
| Strictures of the rectum, Mr. Bell on | 7 | 427 |
| Strictures, lancetted stilettes in | 14 | 195 |

| | Vol. | Page |
|---|---|---|
| Strictures of the œsophagus, cases of, | 15 | 387 |
| Strictures, Mr. Phillips on ........ | 19 | 114 |
| Striking cures .................... | 2 | 234 |
| Structure sliced in search of function | 6 | 262 |
| Strychnia in paralysis ............ | 12 | 130 |
| Strychnia in chronic diarrhœa...... | 12 | 136 |
| Strychnia in amenorrhœa ........ | 12 | 137 |
| Strychnine ...................... | 1 | 49 |
| Strychnine, use of, in paraplegia.... | 10 | 164 |
| Strychnine, partial palsy cured by .. | 11 | 272 |
| Strychnine, amaurosis treated by .. | 13 | 441 |
| Strychnine in amaurosis .......... | 13 | 460 |
| Study of Medicine; new edition .... | 4 | 387 |
| Study of Medicine, 3d edition ...... | 11 | 91 |
| Stuffing for dental gangrene........ | 11 | 333 |
| Stump, French mode of dressing.... | 18 | 176 |
| Stumps, neuralgic affection of...... | 20 | 555 |
| Styptic, Dr. Scudamore's.......... | 1 | 142 |
| Styptics, Mr. Hawkins on ......... | 18 | 38 |
| Subcarbonate of iron, M. Duparque on | 6 | 252 |
| Subcarbonate of iron in gastralgia .. | 18 | 530 |
| Subclavian *tied*, Mr. Key's successful case .................. | 4 | 321 |
| Subclavian, M. Dupuytren's ligature of the.................. | 6 | 163 |
| Subclavian artery, ligature of the .. | 8 | 164 |
| Subclavian tied *beyond* the tumor for aneurism ................ | 11 | 469 |
| Subclavian tied for hæmorrhage .... | 13 | 500 |
| Subclavian, successful ligature of .. | 15 | 482 |
| Subclavian tied with success ...... | 15 | 507 |
| Subclavian artery, ligature of the. .. | 18 | 247 |
| Subjects, on the preservation of.... | 2 | 248 |
| "Subjects," preservation of........ | 9 | 468 |
| Subluxation of the vertebræ, characters of .................. | 3 | 44 |
| Submaxillary gland, fungus hæmatodes of the ................ | 12 | 237 |
| Submaxillary nerves, division of the, | 13 | 271 |
| Suckling infants, duties of the mother | 3 | 488 |
| Suction, employment of, in paracentesis thoracis ............. | 5 | 273 |
| Sudden and mysterious death ..... | 6 | 251 |
| Sudden death, from palsy of the lungs | 19 | 210 |
| Suffocation of children............ | 1 | 351 |
| Suffocation, sudden sense of........ | 9 | 437 |
| Suffocation from ulcer of the throat, | 15 | 381 |
| Suffocative catarrh, on............ | 19 | 441 |
| Sugar an antidote to copper........ | 18 | 528 |
| Suicidal and infanticidal mania .... | 10 | 474 |
| Suicidal wound of throat, fatal .... | 16 | 596 |
| Suicide, proportion of, in different countries .................. | 11 | 429 |
| Sulphate of quinine in erysipelas.... | 1 | 224 |
| Sulphate of quinine in hemicrania .. | 1 | 226 |
| Sulphate of copper, as a test for arsenic | 1 | 322 |
| Sulphate of quinine, remarks on.... | 1 | 487 |
| Sulphate of quinine, M. Bally on.... | 2 | 530 |
| Sulphate of zinc, its powers........ | 3 | 169 |
| Sulphate of quinine, from residuous bark .................... | 3 | 574 |
| Sulphate of copper in chronic diarrhœa | 7 | 156 |
| Sulphate of copper, Dr. Elliotson on, | 8 | 403 |
| Sulphate of quinine, purity of...... | 9 | 207 |
| Sulphate of magnesia made agreeable! | 20 | 438 |
| Sulphuric acid, poisoning by ........ | 9 | 222 |
| Sulphurous acid for anatomical preparations .................. | 9 | 404 |
| Sun's rays, effects of, on the body .. | 9 | 466 |
| Sunderland, cholera in............ | 16 | 280 |
| Sunderland, Dr. Ogden on cholera at, | 16 | 533 |
| Superficial sores on the genitals .... | 15 | 235 |
| Superfœtation, remarkable case of .. | 1 | 181 |
| *Supplantation*, art of; the most approved method .............. | 5 | 211 |
| Supposed union of fractured *cervix femoris* .................... | 3 | 61 |
| Suppressed evacuations, plethora from | 14 | 487 |
| Suppression of urine.............. | 2 | 183 |
| Suppression of urine, on healing an old ulcer ................ | 3 | 240 |
| Suppression and retention of urine .. | 9 | 267 |
| Suppression of habitual evacuations, | 13 | 467 |
| Suppression of urine for seven weeks, | 14 | 468 |
| Suppuration beneath the fascia of the fore-arm ................ | 9 | 527 |
| Suppuration, extensive, after the prick of a pin .............. | 10 | 552 |
| Surgeons, College of, their late bye-law | 1 | 241 |
| Surgeons of hospitals, hints to ..... | 3 | 524 |
| Surgeons; Dr. Armstrong's letter to the College ............... | 4 | 273 |
| Surgeons, Irish College of, curriculum of the ................ | 12 | 446 |
| Surgeons, unpopularity of the College of .................... | 15 | 164 |
| Surgeons, Royal College of, London, | 19 | 565 |
| Surgeons, shades of, in Prussia .... | 20 | 564 |
| Surgery, parallel of French & German | 4 | 213 |
| Surgery, Mr. Liston on ........... | 16 | 242 |
| Surgery, Liston's elements of ..... | 18 | 328 |
| Surgery and physic, intimate union of | 20 | 282 |
| Surgical report of M. M. Roux, Breschet, and Dupuytren...... | 6 | 548 |
| Surgical clinique of St. Louis ...... | 6 | 560 |
| Surgical reform................... | 10 | 500 |
| Surgical and pharmaceutical practice ...................... | 14 | 570 |
| Surgical practitioners, their claims.. | 15 | 166 |
| Surgical anatomy, Mr. Burt's plates of, | 15 | 471 |
| Surgical anatomy, M. Velpeau's.... | 16 | 478 |
| Surgical Essays by Mr. B. Cooper .. | 20 | 137 |
| Sutleffe, Mr. his book of experience, | 2 | 25 |
| Suture, employment of, in vesico-vaginal fistula .............. | 12 | 437 |
| Swan, Mr. on tetanus ............ | 3 | 387 |
| Swan Alley sore ................ | 13 | 167 |
| Swan, Mr. his plates of the nerves .. | 20 | 334 |
| Swan, Mr. on diseases of the nerves, | 20 | 423 |
| Swan, Mr. on the cerebral nerves .. | 18 | 385 |
| Sweating fever, epidemic in France, | 17 | 446 |
| Sweating fever at Auxi-le-Chateau.. | 18 | 521 |
| Switzerland, climate of ..... .... | 11 | 140 |
| Sybille, malignant yellow fever in the, | 14 | 38 |

| | Vol. | Page |
|---|---|---|
| Sycosis menti, M. Biett on | 11 | 502 |
| Sym, Dr. on tetanus | 15 | 497 |
| Syme, Mr. his case of aneurismal dilatation | 10 | 507 |
| Syme, Mr. his treatment ol ulcers | 12 | 474 |
| Syme, Mr. on the excision of joints | 13 | 273 |
| Syme, Mr. on excision of diseased joints | 15 | 113 |
| Symes, Mr. his treatment of incised wounds | 3 | 545 |
| Sympathetic affections of the chest. | 2 | 72 |
| Sympathetic apoplexy, remarkable case | 8 | 455 |
| Sympathetic ganglia, irritation of the, | 12 | 119 |
| Sympathy, Dr. Alison's observations, | 6 | 63 |
| Symphysis pubis, division of, in labour | 4 | 159 |
| Symphysis pubis, division of | 19 | 502 |
| Symptomatic affections of the heart, | 20 | 494 |
| Symptomatology, Dr. Buchan on | 2 | 413 |
| Syncope, its effects on inflammation, | 1 | 141 |
| Syncope anginosa | 6 | 574 |
| Syncope, an effect of loss of blood | 13 | 60 |
| Syncope from hæmorrhage | 13 | 463 |
| Syncope and cerebral congestion | 16 | 253 |
| Syncope, Dr. Hall on | 18 | 355 |
| Synochus gravior, description of the, | 12 | 349 |
| Synovitis, Dr. Couper on | 10 | 457 |
| Syphilis, Mr. Boyle's treatise on | 1 | 172 |
| Syphilis treated by Van Swieten's liquor | 3 | 104 |
| Syphilis, mercury in | 3 | 360 |
| Syphilis and its relatives | 3 | 437 |
| Syphilis, communicated to a fœtus | 7 | 244 |
| Syphilis, Dr. Dzondi's mode of treating | 8 | 206 |
| Syphilis treated without mercury | 9 | 157 |
| Syphilis, new mode of treating | 9 | 157 |
| Syphilis, Mr. Bacot on | 12 | 548 |
| Syphilis, Mr. Travers on | 13 | 165 |
| Syphilis and mercury, compound of, | 13 | 166 |
| Syphilis, primary, cases of | 15 | 225 |
| Syphilis, its first appearance in England | 15 | 405 |
| Syphilis, treated by mercury externally | 18 | 194 |
| Syphilis, cyanuret of mercury in | 18 | 527 |
| Syphilis, primary, Mr. Wallace on | 19 | 374 |
| Syphilis, treatment of | 19 | 381 |
| Syphilis, Dr. Weatherill on | 19 | 449 |
| Syphilis, inoculation of | 19 | 506 |
| Syphilis and syphiloid diseases | 20 | 242 |
| Syphilitic virus inoculated | 1 | 199 |
| Syphilitic ophthalmia, Mr. Hewson on | 2 | 305 |
| Syphilitic and syphiloid pains | 3 | 481 |
| Syphilitic sore from suckling | 11 | 465 |
| Syphilitic iritis, Mr. Lawrence on | 14 | 116 |
| Syphilitic ulceration of the eyelids | 14 | 135 |
| Syphilitic eruptions, M. Biett on | 17 | 369 |
| Syphilitic eruptions, exanthematous, | 17 | 370 |
| Syphilitic eruptions, vesicular | 17 | 370 |
| Syphilitic eruptions, pustular | 17 | 371 |
| Syphilitic eruptions, tubercular | 17 | 372 |
| Syphilitic eruptions, papular | 17 | 373 |

| | Vol. | Page |
|---|---|---|
| Syphilitic eruptions, scaly | 17 | 373 |
| Syphilitic eruptions, diagnosis of | 17 | 374 |
| Syphilitic eruptions, treatment of | 17 | 376 |

### T

| | Vol. | Page |
|---|---|---|
| Tacheron, pathological researches | 2 | 326 |
| Tadpoles, Dr. Edwards on the development of | 18 | 7 |
| Taliacotian operation, Mr. Green's case | 10 | 571 |
| Taliacotian operation | 10 | 588 |
| Taliacotian operation, by M. Velpeau, | 19 | 246 |
| Taliacotian operation, by M. Labat | 19 | 489 |
| Taliacotian operation, by M. Dupuytren | 19 | 502 |
| Talma, post-mortem examination of, | 6 | 465 |
| Tangible signs of pregnancy | 20 | 98 |
| Tape-worm, expelled by the pomegranate root | 3 | 574 |
| Tapping for ovarian dropsy | 12 | 427 |
| Tapping the pericardium, M. Larrey's mode | 15 | 514 |
| Taraxacum and stramonium, their properties | 7 | 100 |
| Taraxacum, good formula for | 10 | 320 |
| Tarentism, account of | 20 | 239 |
| Tarsus and metatarsus, caries of | 15 | 124 |
| Tartar-emetic ointment in epilepsy | 2 | 486 |
| Tartar-emetic, large doses of, in pneumonia | 3 | 573 |
| Tartar-emetic, bark as an antidote for, | 5 | 285 |
| Tartar-emetic plaster in colica pictonum | 7 | 475 |
| Tartar-emetic in large doses employed in 1790 | 11 | 253 |
| Tartar-emetic plasters in chorea | 11 | 483 |
| Tartar-emetic in pneumonia | 18 | 479 |
| Tartar-emetic plaster, death from | 19 | 215 |
| Tartar-emetic in croup | 20 | 212 |
| Tartar-emetic solution for porrigo | 20 | 453 |
| Tartarized antim ny in cholera | 20 | 145 |
| Tartrite of antimony in large doses | 1 | 483 |
| Tartrite of antimony externally applied | 11 | 495 |
| Tartrite of iron and ammonia | 15 | 454 |
| Taste, horrible depravation of | 4 | 282 |
| Tate, Mr. on hysteria | 13 | 30 |
| Tax on those entering the profession, | 13 | 440 |
| Teaching, Dr. Todd's method of | 16 | 45 |
| Teale, Mr. on neuralgic diseases | 12 | 112 |
| Teallier, M. on iliac abscesses and tumors | 12 | 160 |
| Teeling, Dr. on suppression of urine, | 2 | 183 |
| Teeth, nerves of, denuded | 1 | 218 |
| Teeth, effect of general health on the, | 6 | 132 |
| Teeth, necrosis of the | 11 | 335 |
| Teeth, Mr. Bell on the diseases of the | 11 | 408 |
| Teeth as the nuclei of urinary calculi, | 20 | 475 |
| Temperament, prize essay on | 13 | 1 |
| Temperament, Dr. Mayo on | 15 | 35 |
| Temperament, the bilious | 15 | 37 |
| Temperament, the nervous | 15 | 38 |

| | Vol. | Page |
|---|---|---|
| Temperament, the sanguine | 15 | 39 |
| Temperament, the phlegmatic | 15 | 40 |
| Temperature of different localities, table of the | 12 | 94 |
| Temperature, influence of, on decomposition | 12 | 335 |
| Temperature of young animals | 18 | 10 |
| Temperature of adult, warm-blooded animals | 18 | 12 |
| Temperature, high, effects of | 18 | 110 |
| Temporal artery, curious disease of, | 8 | 499 |
| Temporal artery, aneurism of | 9 | 227 |
| Temporal aneurism from arteriotomy | 11 | 523 |
| Tendo Achillis, rupture of the | 7 | 451 |
| Tepid baths, with cold affusions | 18 | 205 |
| Terrene exhalations, as causes of cholera | 16 | 166 |
| Test for contagiousness of cholera | 15 | 500 |
| Testicle, hydatid, Mr. Brodie's case of, | 5 | 595 |
| Testicle, diseases of the | 9 | 277 |
| Testicle, diseased, cured by mercury, | 12 | 231 |
| Testicle, Mr. Russell on | 19 | 169 |
| Testicle, hydatidic, disease of | 20 | 245 |
| Testicles, mode of their descent | 9 | 112 |
| Testis, Sir Astley Cooper on | 13 | 385 |
| Testis, diseased, case of | 13 | 457 |
| Testis, M. Larrey on diseases of the, | 13 | 540 |
| Testis, Sir A. Cooper on the | 14 | 1 |
| Testis, fungoid disease of the | 14 | 1 |
| Testis, scirrhous disease of the | 14 | 9 |
| Testis, venereal inflammation of the, | 14 | 194 |
| Testis, hydatid disease of the | 14 | 200 |
| Testis, fungoid disease of the | 14 | 263 |
| Testis, Sir A. Cooper on the | 14 | 375 |
| Testis, description of the | 14 | 378 |
| Testis, descent of the | 14 | 386 |
| Testis, on chronic inflammation of | 14 | 554 |
| Testis, inflammation of the | 15 | 223 |
| Testis, M. Dupuytren on enlargements of | 18 | 300 |
| Testis, neuralgia of the | 19 | 174 |
| Testis, rheumatism of the | 19 | 174 |
| Testis, gout of the | 19 | 174 |
| Testis, diseased, cases of | 20 | 247 |
| Tests for oxalic acid | 12 | 496 |
| Tetanus, on the use of tobacco in | 1 | 90 |
| Tetanus traumaticus, amputation | 1 | 217 |
| Tetanus, Mr. Carmichael on | 2 | 193 |
| Tetanus, stimulants in | 3 | 283 |
| Tetanus, Mr. Swan on | 3 | 387 |
| Tetanus, a successful case | 4 | 284, 285 |
| Tetanus, some cases of | 6 | 211 |
| Tetanus, tobacco in | 6 | 298 |
| Tetanus, mercurial inunction in | 6 | 304 |
| Tetanus, case of, and lecture on | 6 | 614 |
| Tetanus, M. Broussais on | 7 | 175 |
| Tetanus, acute, a fatal case of | 7 | 232 |
| Tetanus, vapour-bath in | 8 | 520 |
| Tetanus after extirpation of sarcocele, | 8 | 537 |
| Tetanus, carb. of potash & opium in, | 8 | 542 |
| Tetanus cured by tobacco | 9 | 469 |
| Tetanus cured by cupping-glasses | 9 | 507 |
| Tetanus following a burn | 9 | 553 |
| Tetanus from wound of the foot | 9 | 554 |
| Tetanus, subcarbonate of iron in | 11 | 440 |
| Tetanus, Dr. Adams on | 13 | 476 |
| Tetanus, remarks on | 15 | 497 |
| Tetanus, spontaneous | 15 | 569 |
| Tetanus, intermittent | 15 | 570 |
| Tetanus, Dr. Bright on | 16 | 343 |
| Tetanus, cold affusion in | 18 | 526 |
| Tetanus, Mr. Morgan's lecture on | 18 | 536 |
| Tetanus, traumatic, case of | 19 | 528 |
| Tetters (phagedenic) new treatment of, | 3 | 360 |
| Texture, cellular, inflammation of | 2 | 335 |
| Thackrah, on the effects of Trades, &c. on health | 14 | 312 |
| Thackrah, Mr. on cholera | 16 | 441 |
| Thackrah, Mr. on arts, trades, &c. | 18 | 101 |
| Thames water, *purity* of | 8 | 455 |
| Thames water, report on | 9 | 233 |
| Thames & river water, Dr. Lambe on, | 9 | 268 |
| Thames water, spontaneous purification of | 12 | 443 |
| The dead lion and living ass | 15 | 563 |
| Theory, new, of the circulation | 9 | 504 |
| Therapeutical physiology | 1 | 176 |
| Thermal waters of the Andes | 19 | 234 |
| Thickening of bronchial membrane | 3 | 499 |
| Thigh, elongation of; a case | 7 | 255 |
| Thigh, shortening of; cases of | 7 | 257 |
| Thigh and knee-joint, cases of abscess of the | 11 | 456 |
| Thigh-bone, necrosis of | 3 | 48 |
| Thigh-bone, supposed fracture of its neck | 3 | 389 |
| Thigh-bone, Mr. Guthrie on fractured neck of | 4 | 540 |
| Thigh-bone, neck of, fractures | 19 | 83 |
| Thomas, Dr. his physiologie des temperamens | 13 | 1 |
| Thompson, Dr. his new theory of digestion | 11 | 254 |
| Thompson, Dr. on anatomy | 12 | 332 |
| Thompson, Dr. on clouds | 13 | 475 |
| Thompson, Dr. on materia medica | 18 | 321 |
| Thompson, Dr. his classification | 18 | 327 |
| Thompson, Dr. his elements of materia medica | 20 | 403 |
| Thomson, Dr. his case of dissection wound | 3 | 253 |
| Thomson, Dr. his reclamation | 14 | 153 |
| Thoracic disease, severe, case of | 5 | 290 |
| Thoracic diseases, auscultation | 8 | 481 |
| Thoracic diseases, Dr. Bright on | 9 | 452 |
| Thoracis paracentesis | 2 | 479 |
| Thorax, varieties in its anatomy | 9 | 203 |
| Thorax, morbid appearances of, in fever | 12 | 357 |
| Thorax, paracentesis of | 13 | 449 |
| Thorax, fungous tumor of the | 13 | 517 |
| Thorn, Mr. his extract of bals. copaib. | 6 | 499 |
| Thorn, Mr. on sexual diseases | 15 | 412 |

| | Vol. | Page |
|---|---|---|
| Thorpe's, Mr. ligature of subclavian, | 8 | 164 |
| Throat, phagedenic ulcers of this part, | 3 | 473 |
| Throat, leeches in the ............ | 5 | 54 |
| Throat, hæmorrhage from ........ | 13 | 260 |
| Throat, wounds of the............ | 14 | 473 |
| Throat, Velpeau's description of the, | 16 | 481 |
| Throat, fatal suicidal wound of .... | 16 | 596 |
| Throat, Mr. Wood on wounds of.... | 18 | 347 |
| Throat, diseases of, iodine in ...... | 20 | 211 |
| Thwaites, Dr. his case of fungus hæmatodes ................ | 12 | 563 |
| Thymus gland, Mr. Wood on disease of the................. | 6 | 477 |
| Thymus gland, anatomy of the .... | 17 | 139 |
| Thymus gland in the human subject, | 17 | 140 |
| Thymus gland, physiology of the ... | 17 | 142 |
| Thymus gland, disease of.......... | 17 | 144 |
| Thymus gland, development of the .. | 20 | 487 |
| Thyroid arteries tied for bronchocele, | 5 | 585 |
| Tibia, aneurismal tumor in its interior, | 7 | 156 |
| Tibia, case of ununited fracture of .. | 7 | 315 |
| Tibia and fibula, compound fracture, | 10 | 154 |
| Tibia, cases of abscess of the ...... | 18 | 33 |
| Tibial nerve, inflammation of the ... | 13 | 281 |
| Tic-douloureux, cured by caustic .. | 5 | 528 |
| Tic-douloureux, severe case of..... | 7 | 356 |
| Tic-douloureux, Dr. Macculloch on.. | 10 | 49 |
| Tic-douloureux, description of...... | 10 | 50 |
| Tic-douloureux, remarks on ....... | 11 | 357 |
| Tic-douloureux, excision of nerves in | 12 | 470 |
| Tic-douloureux, Sir H. Halford on.. | 15 | 358 |
| Tic-douloureux, case of .......... | 17 | 196 |
| Tic-douloureux of a stump, case of.. | 17 | 489 |
| Tight-lacing, injurious effects of.... | 12 | 512 |
| Tight-lacing, dreadful effects of .... | 15 | 421 |
| Tinct. ferri muriatis .............. | 2 | 359 |
| Tinct. of benzoin in sloughing stump, | 9 | 493 |
| Tinctura colchici, Mr. Bushell on .. | 2 | 531 |
| Tinctura ferri muriatis, as a tonic .. | 3 | 163 |
| Tinctura ferri muriatis, in retention of urine ................ | 3 | 164 |
| Tinea, Dr. Crampton on .......... | 1 | 359 |
| Tinea, treated by warm bath, &c.... | 1 | 360 |
| Tisane de Feltz, its composition .... | 3 | 357 |
| Tissues, various, of the human body, | 12 | 57 |
| Titley, Dr. on elephantiasis of the scrotum.................... | 12 | 505 |
| Toad-fish of Van Dieman's Land.... | 18 | 273 |
| Tobacco in tetanus .............. | 1 | 90 |
| Tobacco in dysentery, Dr. O'Beirne on, | 2 | 495 |
| Tobacco, smoked, in hernia........ | 9 | 525 |
| Tobacco, use of.................. | 14 | 315 |
| Tobacco, as an application in gout.. | 15 | 488 |
| Tobacco, bad effects of smoking .... | 18 | 489 |
| Tod, Mr. on the ear ............. | 17 | 431 |
| Todd, Dr. his book of analysis...... | 16 | 36 |
| Todd, Dr. his analytical scheme .... | 16 | 42 |
| Tommasini on English medicine .... | 1 | 160 |
| Tongue, remarks on the .......... | 1 | 250 |
| Tongue, its different appearances.... | 2 | 424 |
| Tongue, state of, in fever......... | 9 | 126 |
| Tongue, chronic ulcerations of the.. | 9 | 197 |
| Tongue, cancer of the ............ | 11 | 399 |
| Tongue, globular tumor of the..... | 11 | 402 |
| Tongue, appearance of, in disease .. | 15 | 243 |
| Tongue, case of cancer of the ...... | 18 | 194 |
| Tonic treatment of dissection-wounds, | 3 | 251 |
| Tonics, their good effects in amaurosis ................... | 4 | 572 |
| Tonnelé, M. on puerperal fever .... | 13 | 286 |
| Tonospasmia, curious case of ...... | 19 | 509 |
| Tonsil, extirpation of the.......... | 11 | 208 |
| Tonsils, enlargement of—Dr. Belcher, | 2 | 217 |
| Tonsils, removal of the............ | 9 | 284 |
| Tonsils, calomel in inflammation of.. | 13 | 484 |
| Toogood, Mr. on dislocations ...... | 19 | 265 |
| Tooth lodged in the bronchus...... | 20 | 471 |
| Tooth-ache, Dr. Macculloch on .... | 10 | 63 |
| Tooth-ache, applications for ....... | 11 | 334 |
| Tooth-ache, certain remedy for .... | 15 | 457 |
| Tooth-ache, nitric acid in.......... | 16 | 578 |
| Tooth-ache, cured by quinine ..... | 19 | 209 |
| Topographical facts on cholera .... | 16 | 489 |
| Topography of yellow fever........ | 8 | 82 |
| Topography, medical, of Arracan... | 11 | 318 |
| Torsion of the arteries, performed by Clot Bey.... ............. | 20 | 491 |
| Torti on cholera fever in 1700...... | 16 | 303 |
| Touch, examination by, in pregnancy, | 12 | 44 |
| Townsend, Dr. on the stethoscope .. | 9 | 241 |
| Townsend, Dr. on pulmonary apoplexy ..................... | 16 | 470 |
| Toxicological considerations on blood-letting .................. | 10 | 248 |
| Toxicology, Dr. Christison's work on, | 12 | 362 |
| Trachea, great compression of, by a bronchocele............... | 5 | 191 |
| Trachea, lungs, and heart, Dr. Mills on the ..................... | 11 | 75 |
| Trachea and œsophagus, section of.. | 13 | 246 |
| Trachea, wound of the....... .... | 15 | 238 |
| Trachea, foreign body in, 5 months .. | 18 | 535 |
| Tracheotomy in cynanche trachealis, | 2 | 215 |
| Tracheotomy, Mr. Carmichael .... | 2 | 223 |
| Tracheotomy in laryngeal ulceration, | 3 | 475 |
| Tracheotomy, Mr. Goodeve's case of, | 3 | 549 |
| Tracheotomy, some successful cases, | 5 | 433 |
| Tracheotomy, successfully performed, | 7 | 122 |
| Tracheotomy, new mode of performing | 8 | 511 |
| Tracheotomy for angina œdematosa, | 11 | 175 |
| Tracheotomy...................... | 13 | 464 |
| Tracheotomy successful for chronic laryngitis .................. | 14 | 176 |
| Tracheotomy for laryngitis ........ | 15 | 464 |
| Tracheotomy, successful .......... | 15 | 573 |
| Tracheotomy, Mr. Carmichael on .. | 18 | 227 |
| Tracheotomy, case of ............ | 19 | 249 |
| Tracheotomy, successful case of.... | 20 | 473 |
| Trades, &c. their effects on health .. | 14 | 312 |
| Traité des maladies de l'uterus .... | 18 | 406 |
| "Trances," remarks on .......... | 18 | 28 |
| Transactions of the Ed. Med.-Chir. Society ..................... | 1 | 70 |
| Transactions of the Edinb. Society.. | 6 | 61 |

| | Vol. | Page |
|---|---|---|
| Transactions of King and Queen's College, &c. | 1 | 355 |
| Transactions of the Calcutta Medical Society | 5 | 36 |
| Transactions of the Medical Society of Calcutta | 10 | 399 |
| Transactions of the Calcutta Society, | 20 | 361 |
| Transfusion, Dr. Blundell's ideas on | 2 | 410 |
| Transfusion of blood, successful cases, | 4 | 279 |
| Transfusion of blood, Mr. Doubleday's case | 4 | 283 |
| Transfusion, two cases of | 5 | 613 |
| Transfusion, another successful case, | 6 | 575 |
| Transfusion of blood, case of | 7 | 415 |
| Transplantation of a tooth, dreadful consequences from | 3 | 487 |
| Traumatic tetanus, state of the sympathetic in | 3 | 393 |
| Traumatic tetanus in the horse | 4 | 256 |
| Traumatic tetanus cured, a case | 4 | 267 |
| Traumatic erysipelas, actual cautery in, | 5 | 205 |
| Traumatic delirium, M. Dupuytren on, | 8 | 435 |
| Traumatic tetanus, treatment of | 8 | 439 |
| Traumatic tetanus, Mr. Abernethy on, | 9 | 145 |
| Traumatic delirium | 9 | 187 |
| Traumatic delirium, case of | 9 | 479 |
| Traumatic gangrene, cases of, and remarks on | 10 | 145 |
| Traumatic tetanus, pathology of | 10 | 261 |
| Traumatic tetanus, report | 14 | 157 |
| Traumatic tetanus cured by turpentine | 14 | 477 |
| Traumatic tetanus, cases of | 15 | 174 |
| Traumatic tetanus cured | 15 | 215 |
| Traumatic delirium, fatal case of | 15 | 272 |
| Traumatic emphysema, M. Dupuytren on | 18 | 303 |
| Traumatic delirium, M. Dupuytren on | 18 | 306 |
| Traumatic tetanus, case of | 20 | 555 |
| Travellers should be abstemious | 11 | 327 |
| Travelling, Dr. Johnson on | 10 | 520 |
| Travels in Italy in 1820-24, by Dr. Valentin | 6 | 88 |
| Travels in Turkey, by Mr. Madden | 11 | 323 |
| Travers, Mr. on wounds of the belly, | 4 | 548 |
| Travers, Mr. on constitutional irritation | 5 | 95 |
| Travers, Mr. on poisoned wounds | 5 | 363 |
| Travers, Mr. on constitutional irritation | 6 | 101 |
| Travers, Mr. on wounded arteries | 7 | 188 |
| Travers, Mr. on diseased arteries | 7 | 461 |
| Travers, Mr. his curious case of dislocation | 10 | 238 |
| Travers, Mr. on malignant diseases | 11 | 385 |
| Travers, Mr. on venereal affections | 13 | 162 |
| Tread-Mill, remarks on | 1 | 245 |
| Treatment of herniæ | 2 | 211 |
| Treatment of iritis | 2 | 469 |
| Treatment of dysentery pursued at Millbank | 3 | 11 |
| Treatment of tetanus | 3 | 395 |
| Treatment of indigestion | 6 | 39 |
| Trepanning of the spine, case of | 6 | 601 |
| Trephine, Mr. Wise on the use of | 2 | 536 |
| Trephine used for epileptic convulsions | 4 | 592 |
| Trephine, question of the, considered, | 6 | 171 |
| Trephine, observations on its use | 6 | 263 |
| Trephining the spine, Mr. Bell's criticisms on | 3 | 46 |
| Trephining, puncture of dura mater, | 9 | 213 |
| Trephining, case of, at the Hôtel Dieu, | 9 | 447 |
| Trephining, epilepsy cured by | 12 | 504 |
| Trial of Cooper *versus* Wakley | 10 | 268 |
| Trial of Cooper *versus* Wakley, commentary on | 10 | 297 |
| Trial of Jonathan Martin | 11 | 221 |
| Trial of Captain Moir | 14 | 150 |
| Tripe, Mr. his two cases of "dock-yard" fever | 4 | 78 |
| Trismus from cold, cure of | 10 | 468 |
| Trismus traumaticus, case of | 13 | 458 |
| Troillet, M. his paper on globus antiperistalticus | 4 | 519 |
| Troillet, M. on the healing of intestinal ulcers | 5 | 192 |
| True philosophy—opposition | 9 | 438 |
| Truth, Dr. Abercrombie on | 14 | 289 |
| Tube, respiration carried on through, | 15 | 573 |
| Tuber annulare, extravasation in the, | 12 | 458 |
| Tubercle, Dr. Armstrong on the formation of | 11 | 97 |
| Tubercle, Dr. Carswell on | 18 | 367 |
| Tubercle, Dr. Hope on | 18 | 371 |
| Tubercles in the lungs, cicatrization of, | 5 | 214 |
| Tubercles, prize essay on | 8 | 490 |
| Tubercles, Dr. Kellie on the effects of, | 8 | 562 |
| Tubercles, Dr. Baron on the origin and nature of | 9 | 35 |
| Tubercles in the liver, produced by unwholesome food | 9 | 38 |
| Tubercles in the uterus, origin and growth of | 9 | 40 |
| Tubercles, structure of | 9 | 350 |
| Tubercles, Dr. Alison on | 10 | 72 |
| Tubercles, inflammatory, origin of | 10 | 74 |
| Tubercles, hereditary | 10 | 75 |
| Tubercles, prevalence of, in particular classes | 10 | 80 |
| Tubercles of the lungs, signs of | 10 | 501 |
| Tubercles, Medicus on | 10 | 513 |
| Tubercles, origin of | 19 | 201 |
| Tubercular disease of the peritoneum, | 1 | 302 |
| Tubercular abscesses after operations, cases of | 6 | 524 |
| Tubercular affections of the abdomen, | 7 | 334 |
| Tubercular affection of the skin | 9 | 169 |
| Tubercular phthisis, eclectic article on | 12 | 97 |
| Tubercular disease of the abdomen | 15 | 466 |
| Tuberculated peritoneum, a case of | 5 | 285 |
| Tumor, remarkable of a nerve | 1 | 18 |
| Tumor of the neck, cases of | 1 | 371 |
| Tumor in the anterior mediastinum, curious case | 4 | 323 |

| | Vol. | Page |
|---|---|---|
| Tumor of great size, removed | 2 | 219 |
| Tumor growing from the uterus | 10 | 229 |
| Tumor in the orbit—abscess in brain, | 10 | 470 |
| Tumor, bloody, of bone | 11 | 200 |
| Tumor of the dura mater, curious case | 11 | 512 |
| Tumor removed from the spinal nerve | 12 | 184 |
| Tumor, milt-like, Dr. Monro on the, | 12 | 265 |
| Tumor, fungoid, of neck, fatal case of, | 16 | 525 |
| Tumor, parotid, extirpation of a | 16 | 603 |
| Tumor, the remains of a hernial sac, | 17 | 185 |
| Tumor, enormous, attached to ilium, | 18 | 236 |
| Tumor, ovarian, case of | 20 | 177 |
| Tumor, cases of, treated by iodine | 20 | 251 |
| Tumors, hæmorrhoidal | 2 | 276 |
| Tumors, hæmorrhagic, Velpeau | 3 | 226 |
| Tumors, Dr. Good's hypothesis of their growth, &c. | 4 | 389 |
| Tumors of nerves | 6 | 441 |
| Tumors in orbit, cases of | 9 | 253 |
| Tumors, Sir E. Home on | 14 | 41 |
| Tumors becoming malignant; cases, | 14 | 521 |
| Tumors of the abdomen, cases of | 15 | 246 |
| Tumors in the brain, cases of | 16 | 22 |
| Tumors, Mr. Lawrence on | 18 | 333 |
| Tumors, cellular | 18 | 335 |
| Tumors, in the vicinity of parotid | 18 | 336 |
| Tumors, diagnosis of | 18 | 339 |
| Tumors, bloody, of the head | 20 | 210 |
| Tumors, hydatid, of the wrist | 20 | 241 |
| Tumors, encysted, of the head | 20 | 242 |
| Tunica vaginalis, inflammation of | 14 | 23 |
| Tunica vaginalis, cartilaginous bodies in the | 14 | 24 |
| Tunica vaginalis, ossification of the | 14 | 25 |
| Tunica vaginalis, fungoid inflammation of the | 14 | 25 |
| Turkey, Mr. Madden's travels in | 11 | 141 |
| Turkey, the medical art in | 19 | 433 |
| Turkey, poisoning in | 19 | 438 |
| Turn of life | 1 | 496 |
| Turn of life, state at the | 15 | 198 |
| Turnbull, Dr. on veratria | 20 | 405 |
| Turner, Mr. prize hospital report | 7 | 569 |
| Turner, Mr. on sounds of the heart, | 20 | 133 |
| Turning in parturition, Dr. Davis's ideas on | 3 | 426 |
| Turning of the fœtus by the head | 20 | 237 |
| Turpentine, oil of, in sciatica | 10 | 505 |
| Turpentine, oil of, Dr. Moran on the, | 12 | 211 |
| Turpentine, injurious effects of, in gonorrhœa | 20 | 270 |
| Tweedie, Dr. on swelling of the lower extremity | 10 | 180 |
| Tweedie, Dr. on fever | 12 | 337<br>385 |
| Twin-births, instruments in | 3 | 417 |
| Twin-labours, treatment of | 17 | 307 |
| Twining, Mr. on diseases of the spleen | 11 | 314 |
| Twining, Mr. on dysentery | 14 | 332 |
| Twins, curious case of, Mr. Powell | 1 | 131 |
| Twisting of arteries employed to stop hæmorrhage | 11 | 545 |
| Tympanites intestinalis | 1 | 220 |
| Tympanitis of the uterus | 15 | 512 |
| Typhoid or adynamic fever, Dr. Stoker on | 11 | 343 |
| Typhoid fever, types of | 13 | 409 |
| Typhus fever, new treatment of | 1 | 229 |
| Typhus, sulphate of quinine in | 2 | 237 |
| Typhus, its causes | 3 | 82 |
| Typhus, Dr. Clanny's views of | 8 | 510 |
| Typhus fever, Dr. Burne on | 9 | 1 |
| Typhus fever, review of Dr. Burne on, | 9 | 121 |
| Typhus, strong instance of the contagion of | 12 | 308 |
| Typhus, Dr. Smith on | 12 | 353 |
| Typhus, Dr. Tweedie on | 12 | 395 |
| Typhus fever, blood-letting in | 13 | 414 |
| Typhus fever, Dr. O'Brien on | 13 | 414 |
| Typhus, French treatment of | 17 | 444 |
| Typhus, M. Bouillaud on | 20 | 255 |
| Typhus fever, M. Bouillaud on | 20 | 514 |
| Tyrrell, Mr. operations on the spine, | 6 | 601 |
| Tytler, Dr. doctrine of cholera | 20 | 180 |
| **U** | | |
| Ulcer on the surface of the brain, case of | 4 | 207 |
| Ulcer, an odd travelling one | 4 | 241 |
| Ulcer, peculiar, of the eyelids | 7 | 123 |
| Ulcer of the tongue, curious case of, | 7 | 531 |
| Ulcer of the rectum, operated on | 15 | 207 |
| Ulcer of rectum cured by bougie | 16 | 256 |
| Ulcerated prepuce cured by the chlorate of lime | 10 | 222 |
| Ulcerated nævus, case of | 11 | 459 |
| Ulceration of the throat, in the phagedenic disease | 3 | 467 |
| Ulceration of the nostrils, symptoms of | 3 | 468 |
| Ulceration of the throat, syphilitic | 3 | 480 |
| Ulceration of the trachea | 3 | 499 |
| Ulceration of the stomach, Dr. Elliotson's case | 4 | 326 |
| Ulceration of the stomach | 10 | 330 |
| Ulceration of the scrotum—fungus testis, case of | 10 | 465 |
| Ulceration of the auricle of the heart | 11 | 446 |
| Ulceration of the throat, fatal | 13 | 260 |
| Ulceration, carcinomatous, cases of | 15 | 536 |
| Ulceration of heart, case of | 15 | 566 |
| Ulceration in the groin, obstinate and fatal | 18 | 282 |
| Ulceration of the eyelid, Mr. Middlemore on | 20 | 461 |
| Ulcerative process in joints | 20 | 25[illegible] |
| Ulcers of the cornea, Mr. Melin on | 2 | 472 |
| Ulcers, origin of tight bandaging for, | 2 | 532 |
| Ulcers, primary venereal, their treatment | 3 | 453 |
| Ulcers and burns, chloruret of lime in | 6 | 360 |
| Ulcers, simple and varicose, M. Lisfranc on | 6 | 538 |
| Ulcers of the legs, treatment of | 9 | 307 |

N

| | Vol. | Page |
|---|---|---|
| Ulcers, treatment of | 10 | 167 |
| Ulcers of the bowels | 10 | 323 |
| Ulcers, Mr. Stafford on | 12 | 37 |
| Ulcers, Mr. Syme's treatment of | 12 | 474 |
| Ulcers, water dressing in | 12 | 551 |
| Ulcers, primary syphilitic | 19 | 374 |
| Ulcers of the os tincæ | 19 | 522 |
| Ulnar artery wounded—operation | 13 | 139 |
| Ulno-carpal artery, aneurism of, from wound | 11 | 159 |
| Umbilical hernia, remarkable case of, | 12 | 463 |
| Umbilicus, discharge of a fœtus from | 20 | 162 |
| Undercliff, Isle of Wight | 17 | 573 |
| Unguentum argent. nit. in diseases of the eye | 10 | 225 |
| Union by the first intention | 13 | 190 |
| University of London, diploma of | 13 | 204 |
| University of London and Professor Pattison | 15 | 330 |
| University of Edinburgh, causes of its decline | 20 | 315 |
| Ununited fractures, pressure in | 7 | 478 |
| Ununited fracture, cured by pressure | 8 | 555 |
| Ununited fracture—ends of bone excised | 10 | 176 |
| Ununited fracture cured by the seton | 11 | 468 |
| Ureter, obliteration of the | 18 | 192 |
| Ureter, example of a triple | 19 | 504 |
| Urethra, obliteration of | 1 | 215 |
| Urethra, sympathetic irritation of | 3 | 549 |
| Urethra, M. Dupuytren on dilatation of | 7 | 449 |
| Urethra, Mr. Stafford on strictures of the | 11 | 216 |
| Urethra, ruptured, new treatment of, | 13 | 562 |
| Urethra forceps, Mr. Brodie on the | 15 | 258 |
| Urethra, female, dilatation of the | 15 | 265 |
| Urethra, with a reference to lithotrity | 16 | 141 |
| Urethra, Dr. Ducamp on stricture of, | 17 | 49 |
| Urethra, lacerated, fatal cases of | 17 | 533 |
| Urethra, contusions of the | 18 | 443 |
| Urethra, disease of, Mr. Phillips on | 19 | 107 |
| Urethral stricture, observations on | 5 | 570 |
| Urethro-vaginal fistula cured | 4 | 549 |
| Uric acid, Mr. Rodweiss on | 17 | 486 |
| Urinary calculus extracted from the female | 5 | 597 |
| Urinary abscess, treatment of | 10 | 168 |
| Urinary calculi, Dr. England on | 12 | 481 |
| Urinary flux of children, cases of | 13 | 75 |
| Urinary abscess, case of | 18 | 444 |
| Urinary bladder, bilobed, case of | 19 | 235 |
| Urinary bladder, exstrophy of | 19 | 505 |
| Urinary calculi, teeth the nuclei of | 20 | 475 |
| Urine, suppression of | 2 | 183 |
| Urine, passage of substances into | 7 | 404 |
| Urine, chemical properties of in dropsy | 8 | 102 |
| Urine, fatal extravasation of | 12 | 275 |
| Urine, on sand in the | 15 | 131 |
| Urine in diseases of the spine | 17 | 10 |
| Urine, difficulty of passing after gonorrhœa | 17 | 44 |
| Urine, retention of | 17 | 178 |
| Urine, retention of | 19 | 190 |
| Urine, luminous | 19 | 232 |
| Urine, state of the, in children | 19 | 363 |
| Urticaria tuberosa intermittens; case, | 8 | 464 |
| Uterine tumor, Mr. Lightfoot's case of | 3 | 286 |
| Uterine hæmorrhage, plugging of the *uterus* in | 4 | 278 |
| Uterine hæmorrhage, novel treatment of | 5 | 632 |
| Uterine phlebitis, Dr. Lee on | 12 | 376 |
| Uterine irritation, Dr. Addison on | 13 | 40 |
| Uterine irritation, cold injections in, | 13 | 41 |
| Uterine irritation, case of | 13 | 203 |
| Uterine hæmorrhage, compression of the aorta in | 14 | 152 |
| Uterine hæmorrhage | 15 | 26 |
| Uterine inflammation, Dr. Lee on | 15 | 425 |
| Uterine appendages, inflammation of, | 15 | 429 |
| Uterine phlebitis | 15 | 437 |
| Uterine inflammation, causes of | 15 | 440 |
| Uterine inflammation, treatment of | 15 | 445 |
| Uterine, hæmorrhage, Dr. Rambotham on | 17 | 299 |
| Uterine hæmorrhage, cases of | 17 | 301 |
| Uterine hæmorrhage, Mr. Ingleby on, | 17 | 337 |
| Uterine hæmorrhage, internal | 17 | 349 |
| Uterine hæmorrhage, Dr. R. Lee on, | 19 | 67 |
| Uterine hæmorrhage, prevention of | 20 | 446 |
| Uterus, irritable, case of | 2 | 512 |
| Uterus extirpated, dreadful operation | 3 | 265 |
| Uterus, Dr. M'Keever on lacerations of it | 4 | 19 |
| Uterus; fungus of, extirpated by ligature | 4 | 544 |
| Uterus; inflammation of it, causing mania | 5 | 203 |
| Uterus, rupture of, in early pregnancy; a case | 5 | 274 |
| Uterus, leeches to the mouth of the, | 5 | 508 |
| Uterus, inflammatory affections of | 7 | 335 |
| Uterus, Dr. Luroth on the softening of | 9 | 282 |
| Uterus, neuralgiæ of | 9 | 530 |
| Uterus, amputation of the neck of | 9 | 562 |
| Uterus, M. Lisfranc on amputation of the neck of | 10 | 159 |
| Uterus, enlargement of, treated by iodine | 10 | 200 |
| Uterus, amputation of the | 10 | 215 |
| Uterus, tumor of the | 10 | 229 |
| Uterus, fibrous polypus of the | 10 | 373 |
| Uterus, irritable, Dr. Gooch on | 11 | 404 |
| Uterus, successful extirpation of the, | 11 | 564 |
| Uterus, hydatids in the | 12 | 47 |
| Uterus, prolapsus of, case of | 12 | 162 |
| Uterus, peculiar hæmorrhage from the | 12 | 194 |
| Uterus, Dr. Gooch on polypus of the | 12 | 321 |
| Uterus, inverted, extirpation of | 14 | 251 |
| Uterus, Dr. Montgomery on cancer of the | 14 | 266 |
| Uterus and vagina, laceration of | 14 | 357 |

| | Vol. | Page |
|---|---|---|
| Uterus, case of cancer of the | 14 | 475 |
| Uterus, inflammation of its tissue | 15 | 432 |
| Uterus, softening of the | 15 | 432 |
| Uterus, inflammation of its absorbents | 15 | 435 |
| Uterus, tympanitis of | 15 | 512 |
| Uterus to be attended to in hysteria, | 16 | 328 |
| Uterus, singular tumor from the | 17 | 152 |
| Uterus, retroversion of | 17 | 313 |
| Uterus, polypus of | 17 | 315 |
| Uterus, rupture of | 17 | 318 |
| Uterus, apoplexy of | 17 | 319 |
| Uterus, action of the ergot on the | 17 | 341 |
| Uterus, spasmodic contraction of | 17 | 353 |
| Uterus and human ovum, Dr. Seiler on | 18 | 358 |
| Uterus, M. Dugés and Mad. Boivin on diseases of | 18 | 406 |
| Uterus, anteversion of the | 18 | 407 |
| Uterus, hernia of the | 18 | 408 |
| Uterus, unnatural fixedness of the | 18 | 410 |
| Uterus, flexion of the | 18 | 412 |
| Uterus, reversion of os tincæ of | 18 | 413 |
| Uterus, adhesions, &c. of os tincæ of, | 18 | 413 |
| Uterus, incurvations of | 18 | 414 |
| Uterus, retroflexion of; cases | 18 | 417 |
| Uterus, distention of, by gas | 18 | 418 |
| Uterus, distention of, by water | 18 | 419 |
| Uterus, calculi of | 18 | 420 |
| Uterus; moles | 18 | 421 |
| Uterus, degenerations of | 18 | 425 |
| Uterus, vascular degeneration | 18 | 425 |
| Uterus, cellular excrescence of | 18 | 425 |
| Uterus, fibrous and cartilaginous degenerations | 18 | 426 |
| Uterus; tubercular degeneration | 18 | 427 |
| Uterus, irritable, Dr. Davis on the | 19 | 162 |
| Uterus, bilobed, case of | 19 | 235 |
| Uterus, dropsical, case of | 20 | 117 |
| Uterus, hæmorrhage from | 20 | 156 |
| Uterus, retroversion of | 20 | 156 |
| Uterus, respiration of fœtus in the | 20 | 163 |
| Uterus, gravid, hernia of | 20 | 164 |
| Uterus, enormous enlargement of | 20 | 200 |
| Uterus, mucous discharges from | 20 | 222 |
| Uvula, cough from elongation of | 9 | 206 |
| Uvula, prolapsus of the | 13 | 540 |
| Uwins, Dr. on aphonia | 1 | 135 |
| Uwins's, Dr. compendium of medicine | 2 | 382 |
| Uwins, Dr. David, letter to | 12 | 278 |
| Uwins, Dr. on mental disorders | 13 | 93 |
| Uwins, Dr. on mental disorders | 20 | 49 |
| Uwins, Dr. on the character of the Rev. Edw. Irving | 20 | 55 |
| Uwins, Dr. on the curability of insanity | 20 | 56 |
| Uwins, Dr. on asthenic disease of the brain | 20 | 57 |
| Uwins, Dr. on nervous irritability | 20 | 57 |

### V

| | Vol. | Page |
|---|---|---|
| Vaccination, Dr. Gregory on | 6 | 192 |
| Vaccination for nævus maternus | 9 | 170 |
| Vaccination, nævus maternus cured by | 11 | 269 |
| Vaccination of the fœtus in utero | 12 | 214 |
| Vaccination, popular summary of | 12 | 499 |
| Vaccination in France | 19 | 238 |
| Vaccination in Germany | 19 | 481 |
| Vaccination, Lobstein on | 19 | 492 |
| Vaccine establishment, abolition of | 19 | 181 |
| Vaccine, Traité de la, par M. Bousquet | 20 | 417 |
| Vade-mecum of morbid anatomy | 15 | 422 |
| Vagina, artificial anus in the | 9 | 184 |
| Vagina, artificial anus opening into the | 12 | 151 |
| Vagina, imperforate, fatal operation, | 13 | 466 |
| Vagina, imperforate, operation for | 16 | 602 |
| Vagina, abscesses opening into the | 17 | 222 |
| Vagina, cases of contraction of the | 17 | 224 |
| Vagina, imperforate, case of | 18 | 449 |
| Vaginal discharges | 13 | 417 |
| Vaginal discharges in children | 13 | 422 |
| Vaginal plug, Mr. Ingleby on the | 17 | 345 |
| Vaginal cystocele, case of | 18 | 208 |
| Vagitus uterinus | 5 | 524 |
| Valentin, Dr. his Voyage en Italie | 6 | 88 |
| Valerian, extract of, in large doses | 8 | 544 |
| Valvular diseases of the heart | 1 | 193 |
| Valvular obstruction of the rectum | 10 | 208 |
| Valvular disease of heart, sounds in | 16 | 363 |
| Van Swieten's liquor, recipe for | 3 | 101 |
| Van Dieman's Land, toad-fish of | 18 | 273 |
| Vapour-bath, a cheap & effective one, | 3 | 383 |
| Vapour-bath, expeditious | 9 | 460 |
| Vapour-bath for sloughing of penis | 13 | 436 |
| Vapour and warm-baths | 18 | 29 |
| Vapour cave at Pyrmont | 20 | 201 |
| Varicocele, ligature of the spermatic artery for | 11 | 256 |
| Varicocele, Sir A. Cooper on | 14 | 28 |
| Varicocele, M. Delpech on | 16 | 252 |
| Varicocele, veins tied for | 16 | 252 |
| Varicocele, death from operation | 16 | 253 |
| Varicocele, cures of | 16 | 254 |
| Varicocele, on | 19 | 175 |
| Varicose veins, Dr. Briquet's Memoir on | 4 | 245 |
| Varicose veins, excision of | 9 | 556 |
| Varicose ulcers, treatment of | 10 | 162 |
| Varicose veins, treatment of | 10 | 250 |
| Varicose aneurism, M. Dupuytren on | 13 | 509 |
| Varicose tumor | 17 | 184 |
| Variety in diet, Mr. Cameron on | 18 | 94 |
| Variola after vaccination | 1 | 366 |
| Variola after vaccination | 19 | 196 |
| Variola and the varioloid disease | 19 | 487 |
| Variolo-vaccination, Dr. Ferguson's proposal | 3 | 295 |
| Variolous eruption mitigated | 3 | 589 |
| Variolous pustules, cauterization of, | 5 | 285 |
| Variolous ophthalmia | 14 | 414 |
| Variolous epidemic, at Turin | 18 | 529 |
| Varix of the saphena, simulating hernia | 10 | 575 |

| | Vol. | Page |
|---|---|---|
| Varix of the abdominal veins | 19 | 213 |
| Vas deferens, excision of the | 9 | 480 |
| Vascular excitement and inflammation, contrasted | 4 | 435 |
| Vascular inflammation, general, cases of | 6 | 501 |
| Vascular engorgement of spleen, symptoms of the | 11 | 314 |
| Vascular system, Dr. Wise on diseases of the | 17 | 221 |
| Vaughan, Dr. on headaches | 16 | 83 |
| Vein, air introduced to the heart by, | 2 | 218 |
| Vein, excision of a portion of, in varicose legs | 6 | 539 |
| Veins, inflammation of | 2 | 161 |
| Veins, a case of inflammation of | 2 | 447 |
| Veins inflamed, in phlegmasia dolens, fancied cases of | 3 | 234 |
| Veins, several cases of inflammation of the | 4 | 504 |
| Veins of the round ligament, varices of the | 8 | 543 |
| Veins, varicose, excision of | 9 | 556 |
| Veins, purulent concretions in the | 10 | 468 |
| Veins, Mr. Arnott on inflammation of the | 12 | 1 |
| Veins, cases of inflammation of the | 12 | 377 |
| Veins, obstruction of the, in phthisis, | 14 | 206 |
| Veins, obliteration of | 19 | 199 |
| Veins, capillary, inflammation of | 19 | 214 |
| Veitch, Dr. ill-treated | 1 | 237 |
| Velpeau, M. his paper on certain tumors | 3 | 226 |
| Velpeau, M. on the spinal marrow | 4 | 302 |
| Velpeau, M. on pleurisy and abscesses after operations | 6 | 521 |
| Velpeau, M. his surgical anatomy | 16 | 478 |
| Velpeau, M. on fetid abscesses | 17 | 433 |
| Velpeau, M. his operative surgery | 18 | 395 |
| Vena saphena major, inflammation of its cellular coat | 11 | 465 |
| Venables, Dr. on dropsy | 3 | 88 |
| Venables, Dr. on diabetes | 13 | 68 |
| Venereal diseases, Mr. Carmichael | 3 | 435 |
| Venereal poisons, their plurality argued | 3 | 441 |
| Venereal lepra after gonorrhœa, case of | 6 | 606 |
| Venereal affections, Mr. Travers on | 13 | 162 |
| Venereal testis | 13 | 405 |
| Venereal diseases of the eye, Mr. Lawrence on | 14 | 80 |
| Venereal diseases of the eye, Mr. Lawrence on | 14 | 116 |
| Venereal inflammation of the testis | 14 | 194 |
| Venereal diseases, report on | 15 | 221 |
| Venereal disease, M. Ricord on the | 18 | 388 |
| Venereal disease, Mr. Wallace on | 19 | 38, 372 |
| Venereal condylomata, lotion for | 20 | 204 |
| Venereal disease, antiquity of the | 20 | 331 |
| Venereal ulcers, Mr. Johnson on | 20 | 529 |

| | Vol. | Page |
|---|---|---|
| Venereal ulcers, cases of | 20 | 533 |
| Venesection, dangers of | 2 | 336 |
| Venesection, in rheumatism | 2 | 429 |
| Venesection, in repeated abortion | 2 | 519 |
| Venesection, &c. in wounds of large arteries | 3 | 244 |
| Venesection in irritative fever | 4 | 70 |
| Venesection in cholera | 4 | 96 |
| Venesection misapplied, a case of | 4 | 345 |
| Venesection, accidents from | 6 | 193 |
| Venesection, wondrous virtues of | 8 | 247 |
| Venesection, aneurism after | 12 | 476 |
| Venous pulsation, strange instance of | 4 | 92 |
| Venous absorption, Dr. Alison on | 15 | 80 |
| Venus de Medicis | 14 | 456 |
| Veratria, Dr. Bardsley on | 13 | 336 |
| Veratria, Dr. Turnbull on | 20 | 405 |
| Veratria, mode of using | 20 | 406 |
| Veratria in affections of the heart | 20 | 407 |
| Veratria in neuralgic affections | 20 | 411 |
| Veratria in rheumatism | 20 | 413 |
| Veratria in paralysis | 20 | 413 |
| Veratria in dropsy | 20 | 414 |
| Veratrine | 1 | 53 |
| Vermination, Dr. Alexander on | 20 | 449 |
| Vertebræ, displacement of | 10 | 166 |
| Vertebræ, luxation of | 19 | 68 |
| Vertebræ, carious, treated with iodine | 19 | 266 |
| Vertebral column, easy mode of opening it | 4 | 18 |
| Vesicæ, catarrhus, M. Civiale on the, | 11 | 210 |
| Vesical ulceration cured, Dr. Crowther's case | 4 | 573 |
| Vesical fistula, case of, with remarks | 6 | 257 |
| Vesication, difficulty of, in cholera | 3 | 127 |
| Vesication, extemporaneous | 19 | 203 |
| Vesico-vaginal apertures, lunar caustic in | 4 | 281 |
| Vesico-vaginal fistula, M. Dupuytren on | 12 | 271 |
| Vesico-vaginal fistula, Mr. Earle on | 12 | 272 |
| Vesico-vaginal fistula, suture for | 12 | 437 |
| Vesico-vaginal fistula cured by suture | 15 | 160 |
| Vesico-vaginal fistula cured | 17 | 486 |
| Vesico-vaginal fistula, actual cautery in | 18 | 232 |
| Vesicula erythroides, discovery of the | 4 | 576 |
| Vesiculæ seminales, diseases of the | 11 | 459 |
| Vessels of the neck, varieties in their anatomy | 9 | 201 |
| Vessels, state of, in inflammation | 17 | 411 |
| Vetch, Dr. on the spleen | 1 | 460 |
| Vicarious menstruation, case of | 6 | 248 |
| Villermay, M. on inflammation of appendix cœci | 2 | 200 |
| Vincenzo Lanza's New Medical Logic | 5 | 470 |
| Vino-mania, periodical | 10 | 253 |
| Vinum colchici, formulæ for | 9 | 270 |
| Viper, experiments on the bite of the | 5 | 327 |
| Viper-bite, M. Piorry's case of | 6 | 468 |
| Viper-bite, severe effects from | 12 | 175 |
| Virey, M. on the position of the fœtus | 19 | 515 |

| | Vol. | Page |
|---|---|---|
| Viscera, affections of, after injuries.. | 12 | 24 |
| Viscera, affections of, after injuries of the head | 12 | 28 |
| Viscera, affections of, after partu-rition | 12 | 31 |
| Visceral derangement leading to hydrencephalus | 4 | 423 |
| Visceral neuralgia, M. Jolly on | 9 | 529 |
| Visceral depôts, after wounds, &c. | 10 | 169 |
| Visceral neuralgia in a medical gentleman | 10 | 487 |
| Visceral deposites, independent of phlebitis | 12 | 26 |
| Visible signs of pregnancy | 20 | 96 |
| Vision, restoration of | 19 | 156 |
| Vision, double, M. Prevost on | 19 | 211 |
| Vision, effects of jaundice upon | 19 | 444 |
| Vision, yellow, in jaundice | 19 | 447 |
| Vital power, M. Martinet on | 2 | 433 |
| Vital principle, Dr. Prichard on the, | 12 | 316 |
| Vitiated secretions, denied by Broussais | 3 | 331 |
| Vitreous humour regenerated | 17 | 486 |
| Vivisections, remarks on | 3 | 198 |
| Vivisections and experiments, defence of | 5 | 314 |
| Voice, M. Bennati on the | 18 | 79 |
| Voice affected by the velum palati.. | 18 | 80 |
| Voice in many eminent singers | 18 | 82 |
| Voice, influence of the larynx | 18 | 83 |
| Voice, Sir C. Bell on the | 18 | 552 |
| Voice; influence of the trachea and larynx | 18 | 553 |
| Voison, Dr. on the causes of mental diseases | 7 | 1 |
| Volcano of pestilence under London, | 17 | 326 |
| Volta's experiments on malaria | 16 | 490 |
| Vomica opening externally | 13 | 529 |
| Vomica opening over the clavicle | 15 | 566 |
| Vomiting, obstinate, cured by acetate of morphine | 5 | 252 |
| Vomiting in pregnancy | 17 | 337 |
| **W** | | |
| Wadd, Mr. his mems. maxims, &c.. | 7 | 393 |
| Wade, Mr. on fever | 2 | 1 |
| Wagstaff, Mr. on extra-uterine fœtation | 15 | 187 |
| Wake's, Dr. evidence on Martin's trial, | 11 | 223 |
| Wakley on diseases of the heart! .. | 13 | 471 |
| Walcheren fever, Monfalcon's remarks on | 3 | 323 |
| Walker, Dr. his life, by Epps | 15 | 26 |
| Walker, Dr. on cholera in Moscow.. | 16 | 175 |
| Walker, Dr. on diseases of infants.. | 19 | 361 |
| Walking the hospitals | 9 | 196 |
| Walking—Mr. Sheldrake's discoveries | 10 | 554 |
| Wall, Mr. his secret nostrum | 15 | 537 |
| Wallace, Mr. on "fungous eruption," | 8 | 401 |
| Wallace, Mr. on the venereal disease, | 19 | 38, 372 |
| Walther, Dr. his amputation at hip, | 3 | 551 |
| Want of exercise predisposes to disease | 3 | 192 |
| Warburton, Mr. his bill for anatomy, | 11 | 218 |
| Warburton, Mr. his bill; remarks on its loss | 11 | 285 |
| Wardrop, Mr. his edition of Dr. Baillie's works | 4 | 115 |
| Wardrop, Mr. his case of ligature *beyond* the aneurism | 4 | 528 |
| Wardrop, Mr. on the exanthematous ophthalmia | 5 | 393 |
| Wardrop, Mr. case of congenital blindness | 5 | 622 |
| Wardrop, Mr. his case of carotid aneurism | 7 | 209 |
| Wardrop's, Mr. *last* case of aneurism, | 8 | 468 |
| Wardrop, Mr. his case of disease of the temporal artery | 8 | 499 |
| Wardrop, Mr. on aneurism and the new operation | 11 | 46 |
| Wardrop's, Mr. introductory lecture, | 20 | 153 |
| Wardrop, Mr. on effects of bleeding, | 20 | 476 |
| Warm-bath in tinea | 1 | 360 |
| Warm climates, rarity of distortion in, | 3 | 371 |
| Warm climates, Mr. Annesley on the diseases of | 10 | 309 |
| Warren, Dr. on angina pectoris | 9 | 288 |
| Wars and revolutions, their influence on mortality | 9 | 265 |
| Warts, venereal, acetic acid for | 3 | 455 |
| Warts, venereal, lotion for | 20 | 204 |
| Wasting of the testis | 13 | 391 |
| Water, its influence in causing goitre, | 3 | 228 |
| Water, filthy state of, in metropolis.. | 4 | 307 |
| Water, as an external agent. | 18 | 476 |
| Water-dressing, Mr. M'Fadzen on.. | 12 | 551 |
| Watery state of the blood in delirium tremens | 4 | 40 |
| Watson, Dr. on hæmaturia | 17 | 491 |
| Watson, Dr. his introductory lecture, | 20 | 152 |
| Wear and tear, Dr. Johnson on | 14 | 449 |
| Weatherill, Dr. his wonderful case.. | 9 | 518 |
| Weatherill, Dr. his *successful* case.. | 15 | 278 |
| Weatherill, Dr. on syphilis and mercury | 19 | 449 |
| Webster, Dr. on acupuncturation .. | 3 | 562 |
| Webster, Dr. on cholera | 17 | 383 |
| Wedeymeyer, Dr. on paracentesis thoracis | 2 | 213 |
| Weekes, Mr. his case of ruptured stomach | 9 | 505 |
| Weight of new-born children | 1 | 343 |
| Weight of lungs, a test of infanticide, | 1 | 348, 349 |
| Weir, Dr. on delirium tremens | 12 | 568 |
| Weir, Dr. on periostitis | 14 | 481 |
| Weiss, Mr. his reply to Dr. Davis .. | 4 | 295 |
| Weiss' dilator, calculi removed by.. | 18 | 224 |
| Weiss' forceps, calculus extracted by, | 18 | 547 |
| Wellesley Institution, report of the.. | 15 | 21 |
| West India fever, Dr. Wilson on .. | 8 | 74 |
| West Indies, climate of the | 13 | 79 |

| | Vol. | Page |
|---|---|---|
| Wallace, Mr. his work on moxa | 6 | 448 |
| Webster, Dr. his case of cherry-stone in the bronchia | 6 | 228 |
| Westminster Med. Society, its report, | 2 | 558 |
| Westminster Ophthalmic Hospital | 17 | 265 |
| Westminster Ophthalmic Hospital, half-yearly report from | 17 | 284 |
| Westminster Medical Society | 20 | 187 |
| Whatton, Mr. on spinal irritation | 15 | 458 |
| Whey-establishments in Germany | 20 | 197 |
| Whiskey, an injection instead of salt, | 3 | 300 |
| White, Mr. his case of sympathetic irritation of the urethra | 3 | 549 |
| White, Dr. on cholera at Gateshead, | 16 | 541 |
| White-mustard seed, letter on its employment | 6 | 512 |
| White-swelling, employment of iodine in | 4 | 17 |
| White-swelling, M. Lisfranc on | 5 | 546 |
| White-swelling, iodine in | 16 | 124 |
| White-swellings of joints, Dr. Nicolai, | 9 | 42 |
| Whitish stools, or fluxus cœliacus | 7 | 114 |
| Whitlow, Dr. Craigie on | 9 | 170 |
| Whytt, Dr. his anticipation of Dr. Philip | 6 | 303 |
| Wickham's, Mr. prize hospital report, | 8 | 249 |
| Williams, Dr. on auscultation | 10 | 450 |
| Williams, Mr. his evidence on Martin's trial | 11 | 222 |
| Williams, Dr. on the auscultation of the heart | 12 | 155 |
| Williams, Dr. on melanosis | 19 | 263 |
| Williams, Dr. on auscultation | 20 | 132 |
| Williams, Dr. on plessimeters | 20 | 132 |
| Williams, Dr. on sounds of the heart, | 20 | 133 |
| Willis, Dr. on insanity | 2 | 360 |
| Winchester hospital report | 7 | 249 |
| Wine, its too frequent use | 3 | 194 |
| Wines, Dr. Henderson's history of | 4 | 305 |
| Wines, their comparative virtues and strength | 4 | 314 |
| Wise, Mr. on the trephine | 2 | 536 |
| Witt, Dr. his osteology | 20 | 149 |
| Wollaston, Dr. on the optic nerves | 3 | 201 |
| Women, diseases of, Dr. R. Lee on | 19 | 58 |
| Women, fecundity of, in different climates | 20 | 160 |
| Wood, Mr. on purpura hæmorrhagica | 2 | 196 |
| Wood, Mr. on tartar emetic in pneumonia | 3 | 573 |
| Wood, Mr. on wounds of the throat | 18 | 247 |
| Wood, Mr. on inflammation of larynx, | 18 | 343 |
| Wood, Dr. on the skin | 18 | 350 |
| Woodville's experiments on vaccination | 3 | 296 |
| Worm fever, parallel between it and the cerebral | 5 | 219 |
| Worms, intestinal, cause of paralysis, | 11 | 563 |
| Worms in the large bowels | 13 | 213 |
| Worms, production of, in the body | 13 | 221 |
| Worms, discharge of | 19 | 168 |
| Worms, intestinal, on the | 19 | 214 |
| Wound of the heart, a case of | 6 | 245 |
| Wound of the "brachial vein" | 7 | 527 |
| Wound of the knee-joint | 7 | 528 |
| Wound of the larynx and œsophagus, | 7 | 531 |
| Wound of the knee—amputation | 8 | 549 |
| Wound of the anterior tibial artery | 9 | 198 |
| Wound of the heart—pericarditis | 10 | 463 |
| Wound of the femoral artery and vein | 10 | 589 |
| Wound of the femoral vessels in the sheath of the triceps, Mr. Johnson's case | 10 | 594 |
| Wound of the chest and liver, case of, | 15 | 211 |
| Wound of the trachea—aerial fistula, | 15 | 238 |
| Wound of heart, case of | 18 | 453 |
| Wound of the gluteal artery, case of, | 20 | 165 |
| Wound of the intestine, case of | 20 | 182 |
| Wound of the orbit, case of | 20 | 253 |
| Wounded nerves, Mr. G. Bell's cases, | 5 | 245 |
| Wounded arteries, cases of | 7 | 188 |
| Wounded arteries, Mr. Guthrie on | 13 | 133 |
| Wounds of the spinal marrow | 1 | 8 |
| Wounds of nerves | 6 | 426 |
| Wounds of the brain, Mr. Brodie on, | 9 | 417 |
| Wounds of the abdomen | 9 | 527 |
| Wounds in dissection, Mr. Lawrence on | 12 | 529 |
| Wounds of the scalp and their consequences | 14 | 178, 235 |
| Wounds of the throat, M. Larrey on, | 14 | 473 |
| Wounds, M. Larrey's treatment of | 16 | 51 |
| Wounds of the head, M. Larrey on | 16 | 52 |
| Wounds of the face | 16 | 54 |
| Wounds of the eyes | 16 | 55 |
| Wounds of the neck | 16 | 56 |
| Wounds of the chest | 16 | 57 |
| Wounds of the abdomen | 16 | 58 |
| Wounds of the extremities | 16 | 60 |
| Wounds of the thigh | 16 | 64 |
| Wounds of intestines, Dr. Wise on | 18 | 250 |
| Wounds of the bladder, Dr. Macfarlane on | 18 | 443 |
| Wounds of heart | 19 | 84 |
| Wounds of abdomen, B. Cooper on | 20 | 391 |
| Wright, Dr. on arthrosia atonica | 11 | 170 |
| Wright, Dr. his medical report | 13 | 173 |
| Wright, Dr. and Bethlem Hospital | 14 | 471 |
| Wrist, hydatid tumors of the | 20 | 241 |
| Writ, 'de ventre inspiciendo,' | 20 | 123 |
| Wutzer on restored vision | 19 | 158 |

Y

| | Vol. | Page |
|---|---|---|
| Yates, Dr. his letter on cholera | 16 | 606 |
| Yaws, remarks on | 3 | 439 |
| Yeats, Dr. on hydrencephalus | 4 | 406 |
| Yellow fever, its distinctions | 2 | 12 |
| Yellow fever, Dr. Smith's views of | 3 | 78 |
| Yellow fever, Dr. Belcher on | 3 | 568 |
| Yellow fever, very interesting case | 4 | 526 |
| Yellow fever, bleeding and abstinence in | 7 | 54 |

| | Vol. | Page |
|---|---|---|
| Yellow fever, Dr. Wilson on the cause of | 8 | 79 |
| Yellow fever at Plymouth | 9 | 516 |
| Yellow fever, cause of, in the blood, | 13 | 217 |
| Yellow fever, malignant, in the Sybille | 14 | 38 |
| Yellow colour of confectionary analyzed | 15 | 202 |
| Yellow fever, Dr. Gilkrest on | 18 | 362 |
| Yellow fever, treatment of | 18 | 363 |
| Youatt, Mr. on hydrophobia | 13 | 433 |
| Young, Dr. on cholera in India | 16 | 218 |

Z

| | Vol. | Page |
|---|---|---|
| Zink, Dr. on iodine | 2 | 229 |
| Zinn, his case of phlegmatia dolens, | 1 | 379 |
| Zoology, medical, Dr. Stephenson's, | 15 | 126 |

